# Inference of single-cell phylogenies from lineage tracing data using Cassiopeia

**DOI:** 10.1186/s13059-020-02000-8

**Published:** 2020-04-14

**Authors:** Matthew G Jones, Alex Khodaverdian, Jeffrey J Quinn, Michelle M Chan, Jeffrey A Hussmann, Robert Wang, Chenling Xu, Jonathan S Weissman, Nir Yosef

**Affiliations:** 1 Biological and Medical Informatics Graduate Program, University of California San Francisco, San Francisco, CA USA; 2 Center for Computational Biology, University of California Berkeley, Berkeley, CA USA; 3 Department of Electrical Engineering and Computer Science and Center for Computational Biology, University of California Berkeley, Berkeley, CA USA; 4 Howard Hughes Medical Institute, University of California San Francisco, San Francisco, CA USA; 5 Department of Cellular and Molecular Pharmacology, University of California San Francisco, San Francisco, CA USA; 6 Center for RNA Systems Biology, University of California San Francisco, San Francisco, CA USA; 7 University of California, San Francisco, Department of Microbiology and Immunology, San Francisco, California USA; 8 Ragon Institute of Massachusetts General Hospital - MIT and Harvard, Cambridge, MA USA; 9 Chan Zuckerberg Biohub Investigator, San Francisco, CA USA

**Keywords:** scRNA-seq, Single cell, Lineage tracing, CRISPR

## Abstract

The pairing of CRISPR/Cas9-based gene editing with massively parallel single-cell readouts now enables large-scale lineage tracing. However, the rapid growth in complexity of data from these assays has outpaced our ability to accurately infer phylogenetic relationships. First, we introduce Cassiopeia—a suite of scalable maximum parsimony approaches for tree reconstruction. Second, we provide a simulation framework for evaluating algorithms and exploring lineage tracer design principles. Finally, we generate the most complex experimental lineage tracing dataset to date, 34,557 human cells continuously traced over 15 generations, and use it for benchmarking phylogenetic inference approaches. We show that Cassiopeia outperforms traditional methods by several metrics and under a wide variety of parameter regimes, and provide insight into the principles for the design of improved Cas9-enabled recorders. Together, these should broadly enable large-scale mammalian lineage tracing efforts. Cassiopeia and its benchmarking resources are publicly available at www.github.com/YosefLab/Cassiopeia.

The ability to track fates of individual cells during the course of biological processes such as development is of fundamental biological importance, as exemplified by the ground-breaking work creating cell fate maps in *Caenorhabditis elegans* through meticulous visual observation [1, 2]. More recently, CRISPR/Cas9 genome engineering has been coupled with high-throughput single-cell sequencing to enable lineage tracing technologies that can track the relationships between a large number of cells over many generations (Fig. 1a, [3, 4]). Generally, these approaches begin with cells engineered with one or more recording “target sites” where Cas9-induced heritable insertions or deletions (“indels") accumulate and are subsequently read out by sequencing. A phylogenetic reconstruction algorithm is then used to infer cellular relationships from the pattern of indels. These technologies have enabled the unprecedented exploration of zebrafish [5–8] and mouse development [9, 10].

**Fig. 1:**
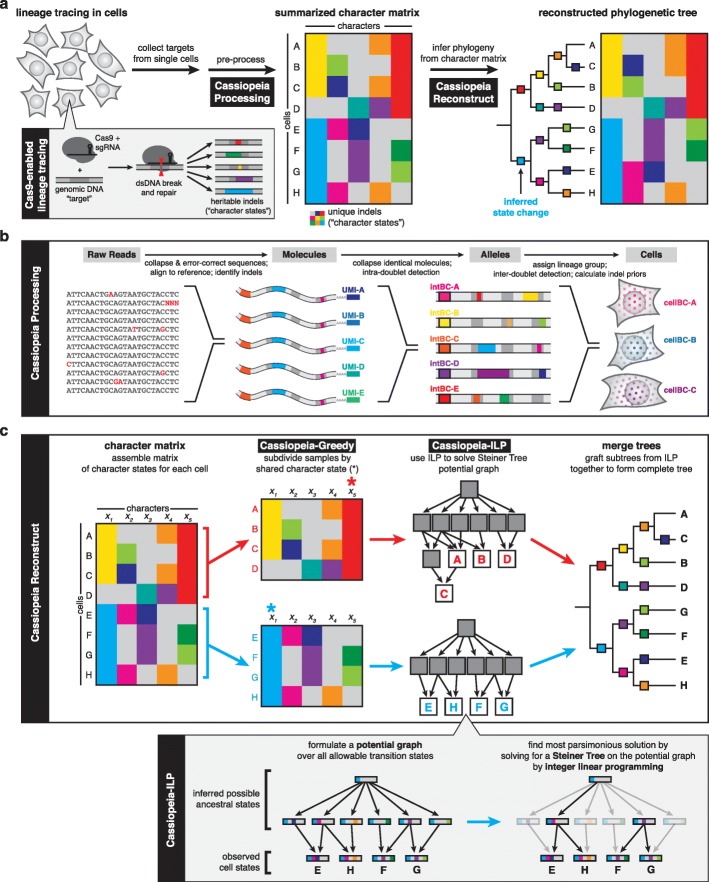
A generalized approach to lineage tracing and lineage reconstruction. **a** The workflow of a lineage tracing experiment. First, cells are engineered with lineage tracing machinery, namely Cas9 that cuts a genomic target site; the target site accrues heritable, Cas9-induced indels (“character states”). Next, the indels are read off from single cells (e.g., by scRNA-seq) and summarized in a “character matrix,” where rows represent cells, columns represent individual target sites (or “characters”), and values represent the observed indel (or “character state”). Finally, the character matrix is used to infer phylogenies by one of various methods. **b** The Cassiopeia processing pipeline. The Cassiopeia software includes modules for the processing of target-site sequencing data: first, identical reads are collapsed together and similar reads are error corrected; second, these reads are locally aligned to a reference sequence and indels are called from this alignment; third, unique molecules are aggregated per cell and intra-doublets are called from this information; finally, the cell population is segmented into clones (or lineage groups) and inter-doublets are called. These clones are then passed to Cassiopeia’s reconstruction module for phylogenetic inference. **c** The Cassiopeia reconstruction framework. Cassiopeia takes as input a “character matrix,” summarizing the mutations seen at heritable target sites across cells. Cassiopeia-Hybrid merges two novel algorithms: the “greedy” (Cassiopeia-Greedy) and “Steiner tree/integer linear programming” (Cassiopeia-ILP) approaches. First, the greedy phase identifies mutations that likely occurred early in the lineage and splits cells recursively into groups based on the presence or absence of these mutations. Next, when these groups reach a predefined threshold, we infer Steiner trees, finding the tree of minimum weight connecting all observed cell states across all possible evolutionary histories in a “potential graph,” using integer linear programming (ILP). Finally, these trees (corresponding to the maximum parsimony solutions for each group) are returned and merged into a complete phylogeny

However, the scale and complexity of the data produced by these methods are rapidly becoming a bottleneck for the accurate inference of phylogenies. Specifically, traditional algorithms for reconstructing phylogenies (such as neighbor joining [11] or Camin-Sokal [12]) have not been fully assessed with respect to lineage tracing data and may not be well suited for analyzing large-scale lineage tracing experiments for several reasons. First, traditional algorithms were developed for the cases of few samples (in this case cells), and thus, scalability is a major limitation (Additional file 1: Fig S1). Second, these algorithms are not well suited to handle the amount of missing data that is typical of lineage tracing experiments, which can be “heritable” (resulting from either large Cas9-induced resections that remove target sites or transcriptional silencing) or “stochastic” (caused by incomplete capture of target sites). Third, these approaches do not explicitly take into consideration the design principles of lineage tracers, such as the irreversibility of mutations or the unedited state of the founder cell. Together, these reasons necessitate the development of an adaptable approach for reconstructing single-cell phylogenies and an appropriate benchmarking resource that can aid in the development of such algorithms.

Ideally, an algorithm for phylogeny inference from lineage tracing data would be robust to experimental parameters (e.g., rate of mutagenesis, the number of Cas9 target sites), scalable to at least tens of thousands of cells, and resilient to missing data. In this study, we introduce Cassiopeia: a novel suite of three algorithms specifically aimed at reconstructing large phylogenies from lineage tracing experiments with special consideration for the Cas9-mutagenesis process and missing data. Cassiopeia’s framework consists of three modules: (1) a greedy algorithm (Cassiopeia-Greedy), which attempts to construct trees efficiently based on mutations that likely occurred earliest in the experiment; (2) a near-optimal algorithm that attempts to find the most parsimonious solution using a Steiner tree approach (Cassiopeia-ILP); and (3) a hybrid algorithm (Cassiopeia-Hybrid) that blends the scalability of the greedy algorithm and the exactness of the Steiner tree approach to support massive single-cell lineage tracing phylogeny reconstruction. To demonstrate the utility of these algorithms, we compare Cassiopeia to existing methods using two resources: first, we benchmark the algorithms using a custom simulation framework for generating synthetic lineage tracing datasets across varying experimental parameters. Second, enabled by a customizable target-site processing pipeline (Fig. 1b), we assess these algorithms using a new reference in vitro lineage tracing dataset consisting of 34,557 cells over 11 clonal populations. Finally, we use Cassiopeia to explore experimental design principles that could improve the next generation of Cas9-enabled lineage tracing systems.

## Results

### Cassiopeia: a scalable framework for single-cell lineage tracing phylogeny inference

Typically, phylogenetic trees are constructed by attempting to optimize a predefined objective over characters (i.e., target sites) and their states (i.e., indels) [13]. Distance-based methods (such as neighbor joining [11, 14, 15] or phylogenetic least-squares [16, 17]) aim to infer a weighted tree that best approximates the dissimilarity between nodes (i.e., the number of characters differentiating two cells should be similar to their distance in the tree). Alternatively, character-based methods aim to infer a tree of maximum parsimony [18, 19]. Conventionally, in this approach, the returned object is a rooted tree (consisting of observed “leaves” and unobserved “ancestral” internal nodes) in which all nodes are associated with a set of character states such that the overall number of changes in character states (between ancestor and child nodes) is minimized. Finally, a third class of methods closely related to character-based ones takes a probabilistic approach over the characters using maximum likelihood [20, 21] or posterior probability [22] as an objective.

We chose to focus our attention on maximum parsimony-based methods due to the early success of applying these methods to lineage tracing data [5, 6] as well as the wealth of theory and applications of these approaches in domains outside of lineage tracing [23]. Our framework, Cassiopeia, consists of three algorithms for solving phylogenies. In smaller datasets, we propose the use of a Steiner tree approach (Cassiopeia-ILP) [24] for finding the maximum parsimony tree over observed cells. Steiner trees have been extensively used as a way of abstracting network connectivity problems in various settings, such as routing in circuit design [25], and have previously been proposed as a general approach for finding maximum parsimony phylogenies [26, 27]. To adapt Steiner trees to single-cell lineage tracing, we devised a method for inferring a large underlying “potential graph” where vertices represent unique cells (both observed and plausible ancestors) and edges represent possible evolutionary paths between cells. Importantly, we tailor this inference specifically to single-cell lineage tracing assays: we model the irreversibility of Cas9 mutations and impute missing data using an exhaustive approach, considering all possible indels in the respective target sites (see the “Methods” section). After formulating the potential graph, we use integer linear programming (ILP) as a technique for finding near-optimal solutions to the Steiner tree problem. Because of the NP-Hard complexity of Steiner trees and the difficult approximation of the potential graph (whose effect on solution stability is assessed in Additional file 1: Fig S2), the main limitation of this approach is that it cannot in practice scale to very large numbers of cells.

To enable Cassiopeia to scale to tens of thousands of cells, we apply a heuristic-based greedy algorithm (Cassiopeia-Greedy) to group cells using mutations that likely occurred early in the lineage experiment. Our heuristic is inspired by the idea of “perfect phylogeny” [28, 29]—a phylogenetic regime in which every mutation (here, Cas9-derived indels) is unique and occurred at most once. For the case of binary characters (i.e., mutated yes/no without accounting for the specific indel), there exists an efficient algorithm [30] for deciding whether a perfect phylogeny exists and if so, to also reconstruct this phylogeny. However, two facets of the lineage tracing problem complicate the deduction of whether or not a perfect phylogeny exists: first, the “multi-state” nature of characters (i.e., each character is not binary, but rather can take on several different states, which makes the problem NP-Hard) [31, 32]; and second, the existence of missing data [33]. To address these issues, we first take a theoretical approach and prove that since the founder cell (root of the phylogeny) is unedited (i.e., includes only uncut target sites) and that the mutational process is irreversible (i.e., edited sites cannot be recut by Cas9), we are able to reduce the multi-state instance to a binary one so that it can be resolved using a perfect phylogeny-based greedy algorithm. Though Cassiopeia-Greedy does not require a perfect phylogeny, we also prove that if one does exist in the dataset, our proposed algorithm is guaranteed to find it (Theorem 1). Secondly, Cassiopeia-Greedy takes a data-driven approach to handle cells with missing data (see the “Methods” section). Unlike Cassiopeia-ILP, Cassiopeia-Greedy is not by design robust to parallel evolution (i.e., “homoplasy,” where a given state independently arises more than once in a phylogeny in different parts of the tree). However, we demonstrate theoretically that in expectation, mutations observed in more cells are more likely to have occurred fewer times in the experiment for sufficiently small, but realistic, ranges of mutation rates (see the “Methods” section; Additional file 1: Fig S3), thus supporting the heuristic. Moreover, using simulations, we quantify the precision of this greedy heuristic for varying numbers of states and mutation rates, finding in general these splits are precise (especially in these regimes of realistic parameterizations; see the “Methods” section and Additional file 1: Fig S4). Below, we further discuss simulation-based analyses that illustrate Cassiopeia-Greedy’s effectiveness with varying amounts of parallel evolution (Additional file 1: Fig S5).

While Cassiopeia-ILP and Cassiopeia-Greedy are suitable strategies depending on the dataset, we can combine these two methods into a hybrid approach (Cassiopeia-Hybrid) that covers a far broader scale of dataset sizes (Fig. 1c). In this use case, Cassiopeia-Hybrid balances the simplicity and scalability of the multi-state greedy algorithm with the exactness and generality of the Steiner tree approach. The method begins by splitting the cells into several major clades using Cassiopeia-Greedy and then separately reconstructing phylogenies for each clade with Cassiopeia-ILP. This parallel approach on reasonably sized sub-problems (∼ 300 cells in each clade) ensures practical run-times on large numbers of cells (Additional file 1: Fig S1). After solving all sub-problems with the Steiner tree approach, we merge all clades together to form a complete phylogeny (Fig. 1c).

### A simulation engine enables a comprehensive benchmark of lineage reconstruction algorithms

**Fig. 2:**
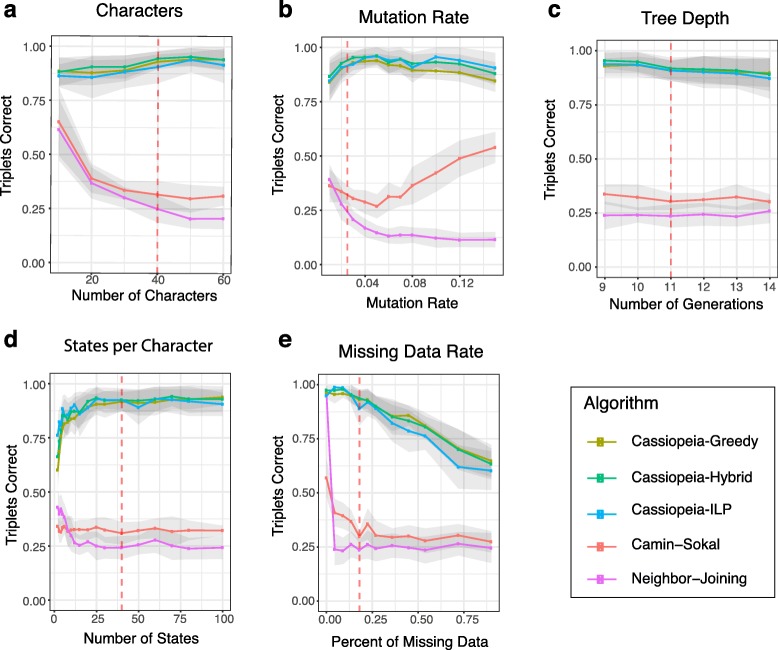
Cassiopeia algorithms outperform other phylogenetic reconstruction methods on simulated lineages. Accuracy is compared between five algorithms (Cassiopeia-Greedy, Cassiopeia-ILP, and Cassiopeia-Hybrid algorithms as well as neighbor joining and Camin-Sokal) on 400 cells. Phylogeny reconstruction accuracy is assessed with the triplets correct statistic across several experimental regimes: **a** the number of characters, **b** the mutation rate (i.e., Cas9 cutting rate), **c** the depth of the tree (or length of the experiment), **d** the number of states per character (i.e., number of possible indel outcomes), and **e** the dropout rate. Dashed lines represent the default value for each stress test. Between 10 and 50 replicate trees were reconstructed, depending on the stability of triplets correct statistic and overall runtime. Standard error over replicates is represented by the shaded area

To provide a comprehensive benchmark for phylogeny reconstruction, we developed a framework for simulating lineage tracing experiments across a range of experimental parameters. In particular, the simulated lineages can vary in the number of characters (e.g., Cas9 target sites), the number of states (e.g., possible Cas9-induced indels), the probability distribution over these states, the mutation rate per character, the number of cell generations, and the amount of missing data. We started by estimating plausible “default” values for each simulation parameter using experimental data (discussed below and indicated in Fig. 2). In each simulation run, we varied one of the parameters while keeping the rest fixed to their default value. The probability of mutating to each state was found by interpolating the empirical distribution of indel outcomes (Additional file 1: Fig S6, see the “Methods” section). Each parameter combination was tested using a maximum of 50 replicates or until convergence, each time sampling a set of 400 cells from the total 2^*D*^ cells (where *D* is the depth of the simulated tree).

We compare the performance of our Cassiopeia algorithms (Cassiopeia-ILP, Cassiopeia-Greedy, and Cassiopeia-Hybrid) as well as an alternative maximum parsimony algorithm, Camin-Sokal (previously used in lineage tracing applications [5, 6]), and the distance-based algorithm neighbor joining. We assess performance using a combinatoric metric, “triplets correct” (Additional file 1: Fig S7, see the “Methods” section), which compares the proportion of cell triplets that are ordered correctly in the tree. Importantly, this statistic is a weighted average of the triplets, stratified by the depth of the triplet (measured by the distance from the root to the latest common ancestor (LCA); see the “Methods” section). As opposed to other tree comparison metrics, such as Robinson-Foulds [34], we reason that combinatoric metrics [35] more explicitly address the needs of fundamental downstream analyses, namely determining evolutionary relationships between cells (though the triplets correct statistic largely agrees with distance-based metrics; see Additional file 1: Fig S7b).

Overall, our simulations demonstrate the strong performance and efficiency of Cassiopeia. Specifically, we see that the Cassiopeia suite of algorithms consistently finds more accurate trees as compared to both Camin-Sokal and neighbor joining (Fig. 2a–e, Additional file 1: Fig S8a-e). Furthermore, not only are trees produced with Cassiopeia more accurate than existing methods, but also more parsimonious across all parameter ranges—serving as an indication that the trees reach a more optimal objective solution (Additional file 1: Fig S9). Importantly, we observe that Cassiopeia-Hybrid and Cassiopeia-Greedy are more effective than neighbor joining in moderately large sample regimes (Additional file 1: Fig S10). Notably, Cassiopeia-Greedy and Cassiopeia-Hybrid both scale to especially large regimes (of up to 50,000 cells, a scale that includes the approximate upper limit of most current single-cell sequencing experiments) without substantial compromise in accuracy (Additional file 1: Fig S11). In contrast, Camin-Sokal and Cassiopeia-ILP could not scale to such input sizes (Additional file 1: Fig S1). Finally, we observe that under a bootstrapping analysis, Cassiopeia’s modules are robust to lineage tracing data (Additional file 1: Fig S12a,b) as compared to neighbor joining for reference (Additional file 1: Fig S12c, though neighbor joining’s stability may be improved with more sophisticated distance functions and feature selection).

These simulations additionally grant insight into critical design parameters for lineage recording technology. Firstly, we observe that the “information capacity” (i.e., number of characters and possible indels, or states) of a recorder confers an increase in accuracy for Cassiopeia’s modules but not necessarily Camin-Sokal and neighbor joining (though they do perform moderately well in low information capacity simulations; Fig. 2a, d). This is likely because the greater size of the search space negatively affects the performance of these two algorithms (in other contexts referred to as the “curse of dimensionality” [36]). In addition to the information capacity, we find that indel distributions that tend towards a uniform distribution (and thus higher entropy) allow for more accurate reconstructions especially when the number of states is small or the number of samples is large (Additional file 1: Fig S13). Unsurprisingly, the proportion of missing data causes a precipitous decrease in performance (Fig. 2e). Furthermore, in longer experiments where the observed cell population is sampled from a larger pool of cells, we find that the problem tends to become more difficult (Fig. 2c).

Furthermore, these results grant further insight into how Cassiopeia-Greedy is affected in regimes where parallel evolution is likely: such as in low information capacity regimes (e.g., where the number of possible indels is less than 10, Fig. 2d) or with high mutation rates (Fig. 2b). In both of these regimes, the proportion of parallel evolution mutations of all mutations increases (Additional file 1: Fig S14). While Cassiopeia-ILP outperforms Cassiopeia-Greedy in these simulations, highlighting its utility to solve small, yet complex, datasets, we further explored Cassiopeia-Greedy’s effectiveness in these regimes. To strengthen our previous theoretical results suggesting that indels observed in more cells are more likely to occur fewer times and earlier in the phylogeny (Additional file 1: Fig S3), we explored how parallel evolution affects Cassiopeia-Greedy empirically with simulation. Specifically, we simulated trees with varying numbers of parallel evolution events at various depths and find overall that while performance decreases with the number of these events, the closer these events occur to the leaves, the smaller thes effect (Additional file 1: Fig S5). Furthermore, we find that under the “default” simulation parameters (as determined by the experimental data; Additional file 1: Fig S6 and 3), Cassiopeia-Greedy consistently makes accurate choices of the first indel event by which cells are divided into clades (Additional file 1: Fig S4b). Of course in regimes where possible, Cassiopeia-ILP outperforms Cassiopeia-Greedy when there are few states (i.e., fewer than 10; Fig. 2d) or high mutation rates (i.e., greater than 10%; Fig. 2b).

**Fig. 3:**
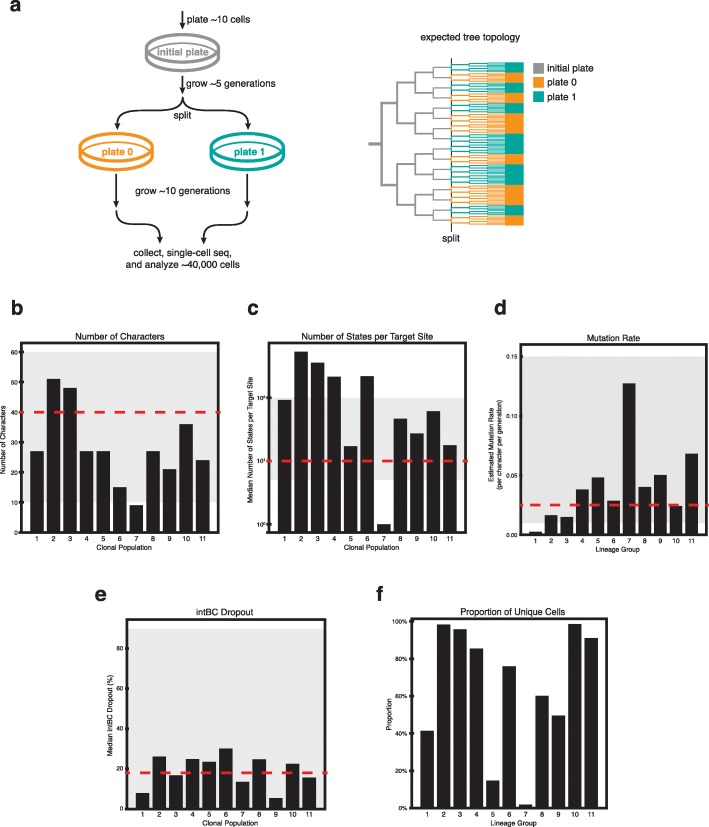
An in vitro reference experiment. **a** A reference lineage tracing dataset was generated using the technology proposed in Chan et al. [10] to human cells cultured in vitro for ∼15 generations. A total of 34,557 cells were analyzed after filtering and error correction. Only the initial split (into two plates) is shown. Analysis of the subsequent split (into four plates) is provided in Additional file 1: Fig S22. **b**–**f** Summary of relevant lineage tracing parameters for each clonal population in the experiment: **b** the number of characters per clone, **c** the number of states per target site, **d** the estimated mutation rate per target site, **e** the median dropout per target site, and **f** the proportion of uniquely marked cells. Gray shading denotes parameter regimes tested in simulations, and red-dashed lines denote the default values for each synthetic benchmarks

Practically, the issue of parallel evolution can be addressed to some extent by incorporating state priors (i.e., probabilities of Cas9-induced indel formation). Ideally, Cassiopeia-Greedy would use these priors to select mutations that are of low probability, but observed at high frequency. Theoretically, this would be advantageous as low-probability indels are expected to occur fewer times in the tree (1); thus, if they appear at high frequency at the leaves, it is especially likely that these occurred earlier in the phylogeny. Furthermore, our precision analysis indicates that Cassiopeia-Greedy’s decisions are especially precise if it chooses an indel with a low prior (Additional file 1: Fig S4). To incorporate these priors in practice, we selected a link function (i.e., one translating observed frequency and prior probability to priority) that maximized performance for Cassiopeia-Greedy (Additional file 1: Fig S15; see the “Methods” section). After finding an effective approach for integrating prior probabilities, we performed the same benchmarks and found that in cases of likely parallel evolution the priors confer an increase in accuracy (e.g., with high mutation rates; Additional file 1: Fig S16), especially in larger regimes (Additional file 1: Fig S11).

Here, we have introduced a flexible simulator that is capable of fitting real data and thus can be used for future benchmarking of algorithms. Using this simulator and a wide range of parameters, we have demonstrated that Cassiopeia performs substantially better than traditional methods. Furthermore, these simulations grant insight into how Cassiopeia’s performance is modulated by various experimental parameters, suggesting design principles that can be optimized to bolster reconstruction accuracy. Specifically, these simulations suggest that these technologies would benefit most from increases in information capacity, via more target sites or more diverse indel outcomes, and mutation rates tuned appropriately as to ensure low rates of parallel evolution. We anticipate that this resource will continue to be of use in exploring design principles of recorders and the effectiveness of novel algorithms.

### An in vitro reference experiment allows evaluation of approaches on empirical data

Existing experimental lineage tracing datasets lack a defined ground truth to test against, thus making it difficult to assess phylogenetic accuracy in practice. To address this, we performed an in vitro experiment tracking the clonal expansion of human cells (A549 lung adenocarcinoma cell line) engineered with a previously described lineage tracing technology [10]. Here, we tracked the growth of 11 clones (each with non-overlapping target site sets for deconvolving clonal populations) over the course of 21 days (approx. 15 generations on average), randomly splitting the pool of cells into two plates every 7 days (Fig. 3a; see the “Methods” section). At the end of the experiment, we sampled approximately 10,000 cells from each of the four final plates. This randomized plate splitting strategy establishes a course-grained ground truth of how cells are related to each other. Here, cells within the same plate can be arbitrarily distant in their lineage; however, there is only a lower bound on lineage dissimilarity between cells in different plates (since they are by definition at least separated by the number of mutations that have occurred since the last split). Thus, overall, on average, we expect cells within the same plate to be closer to each other in the phylogeny than cells from different plates. However, due to the considerations discussed above, we also expect to see some cells more closely related across plates than within (Fig. 3a, right), and indels relating these cells across plates are likely to have occurred before the split.

Our lineage recorder is based on a constitutively expressed target sequence consisting of three evenly spaced cut sites (each cut site corresponding to a character) and a unique integration barcode (“intBC”) which we use to distinguish between target sites and thus more accurately relate character states across cells (Fig. 1b). The target sites are randomly integrated into the genomes of founder cells at high copy number (on average 10 targets per cell or a total of 30 independently evolving characters; Fig. 3b, S18c). We built upon the processing pipeline in our previous work [10] to obtain confident indel information from scRNA-seq reads (Fig. 1b, Additional file 1: Fig S17 & Fig S18, see the “Methods” section for pre-processing procedures and guidelines, especially the “Guidelines for final quality control” section). In addition, we have added modules for the detection of cell doublets using the sets of intBCs in each clone and the indels detected within cells and have determined an effective detection strategy using simulations (see the “Methods” section, Additional file 1: Fig S19). Importantly, though not directly applicable here, this doublet detection can be supplemented by other approaches when transcriptional data [37, 38] or multiplexing barcodes [39] are available. Additionally, we rely on a data-driven approach for estimating the likelihoods of each indel (see the “Methods” section; Additional file 1: Fig S20) because other approaches for indel-likelihood prediction [40–42] may be biased by cell type or cell state.

After quality control, error correction, and filtering, we proceeded with analyzing a total of 34,557 cells across 11 clones. This diverse set of clonal populations represent various levels of indel diversity (i.e., number of possible states, Fig. 3c), size of intBC sets (i.e., number of characters, Fig. 3b and Additional file 1: Fig S18c), character mutation rates (Fig. 3d, see the “Methods” section), and proportion of missing data (Fig. 3e, see the “Methods” section). Most importantly, this dataset represents a significant improvement in lineage tracing experiments: it is the longest and most complex dataset to date in which the large majority of cells, over the entire cell population, have unique mutation states (71% after all quality control and filtering; percentages of unique cells per clone is presented in Fig. 3f), indicating a rich character state complexity for tree building.

**Fig. 4:**
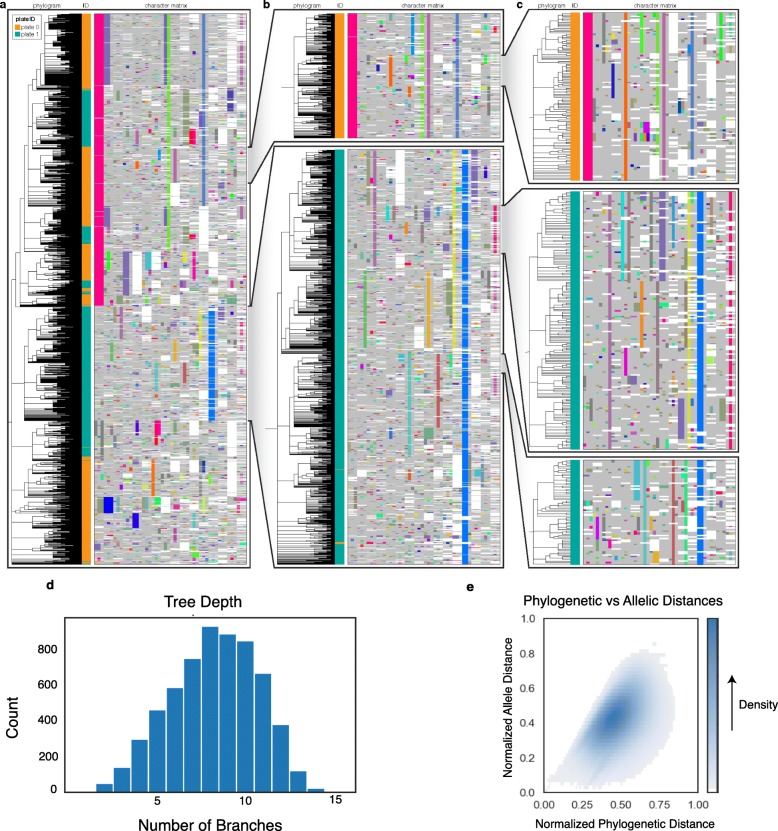
Cassiopeia can reconstruct high-resolution phylogenetic trees from empirical lineage tracing data. The full phylogenetic tree for Clone 3 (**a**), consisting of 7289 cells, was reconstructed using Cassiopeia-Hybrid (with priors) and is displayed. The phylogram represents cell-cell relationships, and each cell is colored by sample ID at the first split (plate 0 or 1). The character matrix is displayed with each unique character state (or “indel”) represented by distinct colors (light gray represents uncut sites; white represents missing values). Of these 7289 cells, 96% were uniquely tagged by their character states. **b**, **c** Nested, expanded views of the phylogram and character matrices. As expected, Cassiopeia correctly relates cells with similar character states, and closely related cells are found within the same culture plate. **d** A histogram of the tree depth of each leaf from the root (mean = 8.22, max = 15). **e** Concordance between normalized allelic distance and normalized phylogenetic distance (see the “Methods” section; Pearson’s correlation = 0.53)

We next reconstructed trees for each clone (excluding two which were removed through quality-control filters; see “Methods” section) with our suite of algorithms, as well as neighbor joining and Camin-Sokal (when computationally feasible). For both Cassiopeia-Greedy and Cassiopeia-Hybrid methods, we also compared tree reconstruction accuracy with or without prior probabilities. The tree for Clone 3, consisting of 7289 cells, along with its character matrix and first split annotations (i.e., whether cells were initially split into plate 0 or plate 1, denoted as the plate ID), is presented in Fig. 4. Interestingly, we find that certain indels indeed span the different plates, thus suggesting that Cassiopeia-Greedy chooses as early splits indels which likely occurred prior to the first separation of plates (though this could also be due to parallel events that occurred independently at each plate). Moreover, the character matrix and the nested dissection of the tree illustrate the abundant lineage information encoded in this clone (96% of the 7289 observed cells have unique mutation states) which allows Cassiopeia to infer a relatively deep tree (Fig. 4d). Despite this complexity, Cassiopeia infers a tree that largely agrees with the observed mutations: cells close to one another in the tree tend to have similar mutations (Fig. 4e).

By keeping track of which plate each cell came from, we are able to evaluate how well the distances in a computationally reconstructed tree reflect the distances in the experimental tree. Thus, we test the reconstruction ability of an algorithm using two metrics for measuring the association between plate ID and substructure: “meta purity” and “mean majority vote” (see the “Methods” section). Both are predicated on the assumption that, just as in the real experiment, as one descends the reconstructed tree, one would expect to find cells more closely related to one another. In this sense, we utilize these two metrics for testing homogeneous cell labels below a certain internal node in a tree, which we refer to as a “clade.”

**Fig. 5:**
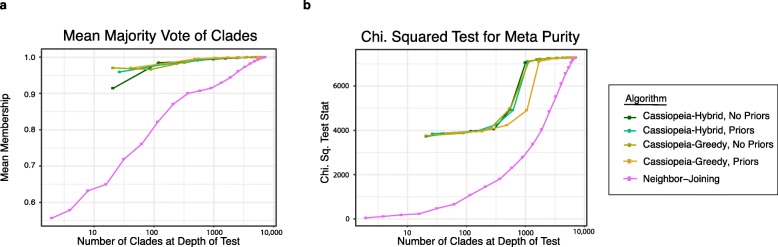
Cassiopeia builds highly accurate trees from large empirical datasets. The consistency between tree reconstructions is evaluated with respect to the first split. The mean majority vote (**a**) and the meta purity test (**b**) were used for Cassiopeia-Hybrid and Cassiopeia-Greedy (both with or without priors) and neighbor joining. The statistics are plotted as a function of the number of clades at the depth of the test (i.e., the number of clades created by a horizontal cut at a given depth). All Cassiopeia approaches consistently outperform neighbor joining by both metrics

We use these statistics to evaluate reconstruction accuracy for Clone 3 with respect to the first split labels (i.e., plate 0 or 1, Fig. 5). In doing so, we find that Cassiopeia-Greedy and Cassiopeia-Hybrid consistently outperform neighbor joining. We find overall consistent results for the remainder of clones reconstructed (Additional file 1: Fig S21, and additionally when considering the subsequent split into four plates—Additional file 1: Fig S21), although Cassiopeia’s modules have the greatest advantage in larger reconstructions. Specifically, Camin-Sokal and neighbor joining perform similarly to Cassiopeia’s modules on clones with few cells (e.g., Clone 11) or with low cell diversity (e.g., Clone 5, where target sites are “exhausted,” possibly due to too-fast cutting, (Fig. 3f, Additional file 1: Fig S23). Both cases indicate that in smaller and less complex clones traditional algorithms may be sufficient for reconstruction. Additionally, many of the issues described previously—parallel evolution, missing data, and information content—contribute to inferential errors in this empirical dataset (for example, Additional file 1: Fig S24).

Overall, we anticipate that this in vitro dataset will serve as a valuable empirical benchmark for future algorithm development. Specifically, we have demonstrated how this dataset can be used to evaluate the accuracy of inferred phylogenies and illustrate that Cassiopeia consistently outperforms neighbor joining for the purposes of reconstructing trees from single-cell lineage tracing technologies. Moreover, we demonstrate Cassiopeia’s scalability for reconstructing trees that are beyond the abilities of other maximum parsimony-based methods like Camin-Sokal as they currently have been implemented.

### Generalizing Cassiopeia to alternative and future technologies

**Fig. 6:**
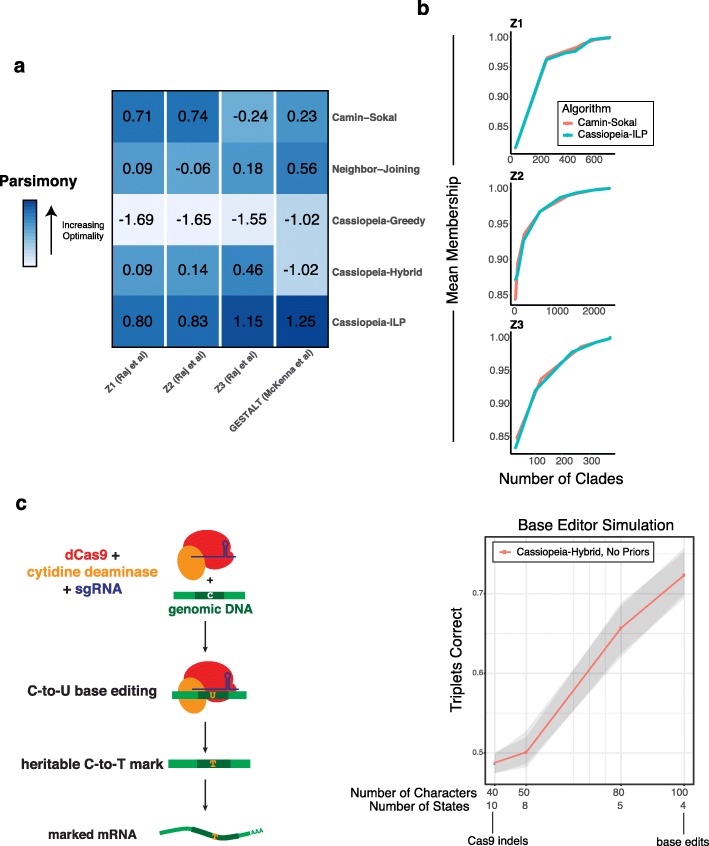
Generalizing Cassiopeia and future design principles of CRISPR-enabled lineage tracers. **a** Cassiopeia generalizes to alternative lineage tracing methods, as illustrated with the analysis of data from GESTALT technology [5, 6]). In a comparison of parsimony across Camin-Sokal, neighbor joining, and Cassiopeia’s methods, the Steiner tree approach consistently finds more parsimonious (i.e., more optimal) solutions. *Z*-scores for each dataset are annotated over each tile. **b** Biological integrity of trees for each zebrafish from Raj et al. [6], inferred with Cassiopeia-ILP, was assessed using the mean membership statistic (the ”Methods” section) with respect to tissue-type annotations from the original study. **c** Exploring information capacity of recorders with base editors. A theoretical base editor was simulated for 400 cells and reconstructions with Cassiopeia-Hybrid, with and without priors. We compared the accuracy of the reconstructions to the simulated tree using the triplets correct statistic. We describe the performance of Cassiopeia-Hybrid as the number of characters was increased (and consequently number of states was decreased)

While previous single-cell lineage tracing applications have proposed methods for phylogenetic reconstruction, they have been custom-tailored to the experimental system, requiring one to filter out common indels [7] or provide indel likelihoods [10]. We thus investigated how well Cassiopeia generalizes to other technologies with reconstructions of data generated with the GESTALT technology applied to zebrafish development [5, 6] (Fig. 6a, Additional file 1: Fig S25). Comparing Cassiopeia’s algorithms to neighbor joining and Camin-Sokal (as applied in these previous studies [5, 6]), we find that Cassiopeia-ILP consistently finds the most parsimonious solution. Furthermore, the mean majority vote statistic also indicates that there is strong tissue-type enrichment as a function of tree depth, agreeing with Camin-Sokal’s reconstruction which was used in the original study [6] (Fig. 6b). Together, these results clearly demonstrates Cassiopeia’s effectiveness for existing alternative lineage tracing technologies.

After establishing Cassiopeia’s generalizability, we turned to investigating plausible next-generation lineage tracers. Recently, base-editing systems (Fig. 6c) have been proposed to precisely edit *A*>*G* [43], *C*>*T* [44, 45], or possibly *C*>*N* (*N* being any base as in [46]). The promise of base-editing lineage recorders is threefold: first, a base editor would increase the number of editable sites (as compared to the ones that rely on Cas9-induced double-strand breaks [5, 7, 10]) although at the expense of the number of states (at best 4, corresponding to A, C, T, and G). Second, a base-editing system would theoretically result in less dropout, since target site resection via Cas9-induced double-strand breaks is far less likely [44]. Third, it is hypothesized that base editors would be less cytotoxic as it does not depend on inducing double-strand breaks on DNA (although this relies on effective strategies for limiting off-target base editing of DNA and RNA [47]). To evaluate the application of base editors for lineage tracing, we tested the performance of Cassiopeia in high-character, low-state regimes as would be the case in base editing (Fig. 6c, see the “Methods” section). Using simulations with parameters deduced by a recent base editor application [46], we demonstrate that there appears to be an advantage of having more characters than states (Fig. 6c). Of note, we did not observe any substantial deviation in these simulations from our initial scalability benchmarks in Additional file 1: Fig S1. This suggests that base editors may be a promising future direction for lineage tracing from a theoretical perspective.

Another potentially promising design consideration concerns the range of character mutation rates and their variability across different target sites—a parameter that can be precisely engineered [48]. In this design, one would expect the variability to help distinguish between early and late branching points and consequently achieve better resolution of the underlying phylogeny [9, 49, 50]. We simulated “Phased Recorders” (Additional file 1: Fig S26) with varying levels of target-site cutting variability and observe that this design allows for better inference when the distributions of mutation probabilities are more dispersed (Additional file 1: Fig S26b). This becomes particularly useful when one can integrate accurate indel priors into Cassiopeia.

Overall, these results serve to illustrate how Cassiopeia and the simulation framework can be used to explore experimental designs. While there inevitably will be challenges in new implementations, these analyses demonstrate theoretically how design parameters can be optimized for downstream tree inference. In this way, the combination of our algorithms and simulations enables others to explore not only new algorithmic approaches to phylogenetic reconstruction but also new experimental approaches for recording lineage information.

## Conclusions

In this study, we have presented three resources supporting future single-cell lineage tracing technology development and applications. Firstly, we described Cassiopeia, a scalable and accurate maximum parsimony framework for inferring high-resolution phylogenies in single-cell lineage tracing experiments. Next, we introduced a simulation approach for benchmarking reconstruction methods and investigating novel experimental designs. Finally, we generated the largest and most diverse empirical lineage tracing experiment to date, which we present as a reference for the systematic evaluation of phylogeny inference on real lineage tracing data. With the combination of these three resources, we have demonstrated the improved scalability and accuracy of Cassiopeia over traditional approaches for single-cell lineage tracing data and have explored design principles for more accurate tracing. To ensure broad use, we have made a complete software package, including the algorithms, the simulation framework, and a processing pipeline for raw data, all publicly available at www.github.com/YosefLab/Cassiopeia.

The results highlighted in this manuscript demonstrate the variability in reconstruction accuracy for each of Cassiopeia’s modules depending on the parameters. As introduced here, we suggest using Cassiopeia-ILP for small regimes (fewer than 200 cells) especially where there is low information capacity, Cassiopeia-Greedy for extremely large regimes (10,000 cells and larger), and Cassiopeia-Hybrid for intermediate regimes. Ideally, Cassiopeia-Hybrid could be run in all situations and transition appropriately between Cassiopeia-Greedy and Cassiopeia-ILP depending on the complexity of the data. While here we use the number of cells as the criterion for transitioning, we anticipate there is a more consistent statistic (e.g., the entropy of a group of cells) for controlling the Cassiopeia-Hybrid transition that will make Cassiopeia more intuitive and effective with handling real data.

Though we illustrate that Cassiopeia provides the computational foundation necessary for future large-scale lineage tracing experiments, there are several opportunities for future improvement. First, the inclusion of prior probabilities increases Cassiopeia’s performance only when parallel evolution is likely (e.g., with a high per-character mutation rate or in low character state regimes). While maximum parsimony methods are attractive due to their non-parametric nature, future studies may build on our work here by developing more powerful approaches for integrating prior mutation rates into maximum likelihood [20, 21] or Bayesian inference [51] frameworks, perhaps relying on recent literature that seeks to predict indel formation probabilities [40–42]. Future work in this space may also focus on using maximum parsimony solutions to further refine solutions in an effort to resolve branch length as with GAPML [52] or with paired transcriptomic observations [53]. Second, there exists a promising opportunity in developing new approaches for better handling of missing data. Determining a model which explicitly distinguishes between stochastic and heritable missing data may increase tree accuracy. Alternatively, adapting supertree methods (such as the Triple MaxCut algorithm [54]) for lineage tracing data may be an interesting direction as they have been effective for dealing with missing data (but only when this missing data is randomly distributed [55]). Aside from computational approaches for dealing with missing data, it is still unclear how much missing data is due to silencing, Cas9-resections, or stochastic dropout and experiments to elucidate the contributions of each will be helpful to the future design of lineage tracers. Third, while we provide theoretical and empirical evidence for our greedy heuristic, we note that there are opportunities for developing other heuristics—for example, by considering mutations in many characters rather than a single mutation as we do or using a distance-based heuristic.

The ultimate goal of using single-cell lineage tracers to create precise and quantitative cell fate maps will require sampling tens of thousands of cells (or more), possibly tracing over several months, and effectively inferring the resulting phylogenies. While recent studies [56] have highlighted the challenges in creating accurate CRISPR-recorders, our results suggest that with adequate technological components and computational approaches complex biological phenomena can be dissected with single-cell lineage tracing methods. Specifically, we show that Cassiopeia and the benchmarking resources presented here meet many of these challenges. Not only does Cassiopeia provide a scalable and accurate inference approach, but also our benchmarking resources enable the systematic exploration of more accurate algorithms as well as more robust single-cell lineage tracing technologies. Taken together, this work forms the foundation for future efforts in building detailed cell fate maps in a variety of biological applications.

## Methods

### In vitro lineage tracing experiment

#### Plasmid design and cloning

The Cas9-mCherry lentivector, pHR-UCOE-SFFV-Cas9-mCherry (to be added to Addgene), was designed for stable, constitutive expression of enzymatically active Cas9, driven by the viral SFFV promoter, insulated with a minimal universal chromatin opening element (minUCOE), and tagged with C-terminal, self-cleaving P2A-mCherry. pHR-UCOE-SFFV-Cas9-mCherry is derived from pMH0001 (Addgene Cat#85969, active Cas9) with the BFP tag exchanged with mCherry. The P2A-mCherry tag was PCR amplified from pHR-SFFV-KRAB-dCas9-P2A-mCherry (Addgene Cat #60954; forward: GAGCAACGGCAGCAGCGGATCCGGAG-CTACTAACTTCAG; reverse: ATATCAAGCTTGCATGCCTGCAGGTCGACTTACTACTTGTACAGCTCGTC-CATGC) and inserted using Gibson Assembly (NEB) into SbfI/BamHI-digested pMH0001 (active Cas9). Resulting plasmid was used for lentiviral production as described below.

The Target Site lentivector, PCT48 (to be added to Addgene), was derived from the reverse lentivector PCT5 (to be added to Addgene) containing GFP driven by the EF1a promoter. The sequence of the 10X amplicon with most common polyA location is the following: AATCCAGCTAGCTGTGCAGCNNNNNNNNNNNNNNATTCAACTGCAGTAATGCTACCTCGTACTCACGCTTTCCAAGTGCTTGGCGTCGCATCTCGGTCCTTTGTACGCCGAAAAATGGCCTGACAACTAAGCTACGGCACGCTGCCATGTTGGGTCATAACGATATCTCTGGTTCATCCGTGACCGAACATGTCATGGAGTAGCAGGAGCTATTAATTCGCGGAGGACAATGCGGTTCGTAGTCACTGTCTTCCGCAATCGTCCATCGCTCCTGCAGGTGGCCTAGAGGGCCCGTTTAAACCCGCTGATCAGCCTCGACTGTGCCTTCTAGTTGCCAGCCATCTGTTGTTTGCCCCTCCCCCGTGCCTTCCTTGACCCTGGAAGGTGCCACTCCCACTGTCCTTTCCTAATAAAAAAAAAAAAAAAAAAAAAAA

where *N* denotes our 14-bp random integration barcode. PCT5 was digested with SfiI and EcoRI within the 3 ^′^UTR of GFP. The Target Site sequence was ordered as a DNA fragment (gBlock, IDT DNA) containing three Cas9 cut sites and a high diversity, 14-bp randomer (integration barcode, or intBC). The fragment was PCR amplified with primers containing Gibson assembly arms compatible with SfiI/EcoRI-digested PCT5 (forward: GATGAGCTCTACAAATAATTAATTAAGAATTCGTCACGAATCCAGCTAGCTGT;reverse:GGTTTAAACGGGCCCTCTAGGC CACCTGCAGGAGCGATGG). The amplified Target Site fragment was inserted into the digested PCT5 backbone using Gibson Assembly. The assembled lentivector library was transformed into MegaX competent bacterial cells (Thermo Fisher) and grown in 1L of LB with carbenicillin at 100 *μ*g/mL. Lentivector plasmid was recovered and purified by GigaPrep (Qiagen) and used for high-diversity lentiviral production as described below.

The triple-sgRNA-BFP-PuroR lentivector, PCT61 (to be added to Addgene), is derived from pBA392 (to be added to Addgene) as previously described [57, 58] containing three sgRNA cassettes driven by distinct U6 promoters and constitutive BFP and puromycin-resistance markers for selection. Importantly, the three PCT61 sgRNAs are complementary to the three cut sites in the PCT48 Target Site. To slow the cutting kinetics of the sgRNAs to best match the timescale involved in the in vitro lineage tracing experiments [10], the sgRNAs contain precise single-basepair mismatches that decrease their avidity for the cognate cut sites [59]. The triple-sgRNA lentivector was cloned using four-way Gibson assembly as described in [58]. Resulting plasmid was used for lentiviral production as described below.

#### Cell culture, DNA transfections, viral preparation, and cell line engineering

A549 cells (human lung adenocarcinoma line, ATCC CCL-185) and HEK293T were maintained in Dulbecco’s modified Eagle medium (DMEM, Gibco) supplemented with 10% FBS (VWR Life Science Seradigm), 2 mM glutamine, 100 units/mL penicillin, and 100 *μ*g/mL streptomycin. Lentivirus was produced by transfecting HEK293T cells with standard packaging vectors and TransIT-LTI transfection reagent (Mirus) as described in ([57]). Target Site (PCT48) lentiviral preparations were concentrated tenfold using Lenti-X Concentrator (Takara Bio). Viral preparations were frozen prior to infection. Triple-sgRNA lentiviral preparations were titered and diluted to a concentration to yield approximately 50% infection rate. To construct the lineage tracing-competent cell line, A549 cells were transduced by serial lentiviral infection with the three lineage tracing components: (1) Cas9, (2) Target Site, and (3) triple-sgRNAs. First, A549 cells were transduced by Cas9 (mCherry) lentivirus and mCherry+ cells were selected to purity by fluorescence-activated cell sorting on the BD FACS Aria II. Second, A549-Cas9 cells were transduced by concentrated Target Site (GFP) lentivirus and GFP+ cells were selected by FACS; after sorting, Target Site infection and sorting were repeated two more times for a total of three serial lentiviral transfections, sorting for cells with progressively higher GFP signal after each infection. This strategy of serial transfection with concentrated lentivirus yielded cells with high copy numbers of the Target Site, which were confirmed by quantitative PCR. Third, A549 cells with Cas9 and Target Site were transduced by titered triple-sgRNA (BFP-PuroR) lentivirus and selected as described below.

#### In vitro lineage tracing experiment, single-cell RNA-seq library preparation, and sequencing

One day following triple-sgRNA infection, cells were trypsinized to a single-cell suspension and counted using an Accuri cytometer (BD Biosciences). Approximately 25 cells were plated in a single well of a 96-well plate. Seven days post-infection, cells were trypsinized and split evenly into two wells of a 96-well plate. Cells stably transduced by triple-sgRNA lentivirus were selected by adding puromycin at 1.5 *μ*g/mL on days 9 and 11 post-infection; puromycin-killed cells were removed by washing the plate with a fresh medium. After 14 days, cells were trypsinized and split evenly for a second time into four wells of a 6-well plate. Finally, after 21 days in total, cells from the four wells were trypsinized to a single-cell suspension and collected.

Cells were washed with PBS with 0.04% w/v bovine serum albumin (BSA, New England Biolabs), filtered through 40 *μ*m FlowMi filter tips filter tips (Bel-Art), and counted according to the 10x Genomics protocol. Approximately 14,000 cells per sample were loaded (expected yield: approximately 10,000 cells per sample) into the 10x Genomics Chromium Single Cell 3 ^′^ Library and Gel Bead Kit v2, and cDNA was reverse-transcribed, amplified, and purified according to the manufacturer’s protocol. Resulting cDNA libraries were quantified by BioAnalyzer, yielding the expected size distribution described in the manufacturer’s protocol.

To prepare the Target Site amplicon sequencing library, resulting amplified cDNA libraries were further amplified with custom, Target Site-specific primers containing P5/P7 Illumina adapters and sample indices (forward:CAAGCAGAAGACGGCATACGAGATXXXXXXXXGTCTCGTGGGCTCGGAGATGTGTATAAGAGACAGAATCCAGCTAGCTGTGCAGC;reverse:CAAGCAGAAGACGGCATACGAGATXXXXXXXXGTCTCGTGGGCTCGGAGATGTGTATAAGAGACAGGCATGGACGAGCTGTACAAGT; “X” denotes sample indices). PCR amplification was performed using Kapa HiFi HotStart ReadyMix, as in [57], according to the following program: melting at 95^∘^C for 3 min, then 14 cycles at 98 ^∘^C for 15 s and 70 ^∘^C for 20 s. Approximately 12 fmol of template cDNA were used per reaction; amplification was performed in quadruplicate to avoid PCR-induced library biases, such as jack-potting. PCR products were re-pooled and purified by SPRI bead selection at 0.9x ratio and quantified by BioAnalyzer.

Target Site amplicon libraries were sequenced on the Illumina NovaSeq S2 platform. Due to the low sequence complexity for the Target Site library, a phiX genomic DNA library was spiked in at approximately 50% for increased sequence diversity. The 10x cell barcode and unique molecular identifier (UMI) sequences were read first (R1: 26 cycles) and the Target Site sequence was read second (R2: 300 cycles); sample identities were read as indices (I1 and I2: 8 cycles, each). Over 550M sequencing clusters passed filter and were processed as described below. All raw and processed data are available through GEO Series accession GSE146712 [60].

### Processing pipeline

#### Read processing

Each target site was sequenced using the Illumina Nova-seq platform, producing 300-bp long-read sequences. The Fastq’s obtained were quantitated using 10x’s cellranger suite, which simultaneously corrects cell barcodes by comparing against a whitelist of 10x’s approved cell barcodes. For each cell, a consensus sequence for each unique molecule identifier (UMI) was produced by collapsing similar sequences, defined by those sequences differing by at most 1 Levenshtein distance. A directed graph is constructed, where sequences with identical UMIs are connected to one another if the sequences themselves differ by at most one Levenshtein distance. Then, UMIs in this network are collapsed onto UMIs that have greater than or equal number of reads. This produces a collection of sequences indexed by the cell barcode and UMI information (i.e., there is a unique sequence associated with each UMI).

Before aligning all sequences to the reference, preliminary quality control is performed. Specifically, in cases where UMIs in a given cell still have not been assigned a consensus sequence, the sequence with the greatest number of reads is chosen. UMIs with fewer than 2 reads are filtered out, and cells with fewer than 10 UMIs are filtered out as well. Finally, a filtered file in Fastq format is returned.

#### Allele calling

Alignment is performed with Emboss’s Water local alignment algorithm. Optimal parameters were found by performing a grid search of gap open and gap extend parameters on a set of 1000 simulated sequences, comparing a global and local alignment strategy. We found a gap open penalty of 20.0 and a gap extension penalty of 1.0 produced optimal alignments. The “indels” (insertions and deletions resulting from the Cas9-induced double-strand break) at each cut site in the sequences are obtained by parsing the cigar string from the alignments. To resolve possible redundancies in indels resulting from Cas9 cutting, the 5 ^′^ and 3 ^′^ flanking 5-nucleotide context is reported for each indel.

#### UMI error correction

To correct errors in the UMI sequence either introduced during sequencing, PCR preparation, or data processing, we leverage the allele information. UMIs are corrected within groups of identical cell barcode-integration barcode pairs (i.e., we assume that only UMIs encoding for the same intBC in a given cell can be corrected). We reason that ideally, for a given integration barcodes, a cell will only report one sequence, or allele. Within these “equivalence classes,” UMIs that differ by at most 1 Levenshtein distance (although this number can be user-defined) are corrected towards the UMI with a greater number of reads.

#### Cell-based filtering

With the UMI corrected and indels calculated, the new “molecule table” is subjected to further quality control. Specifically, UMIs are filtered based on the number of reads (dynamically set to be the 99th percentile of the reads divided by 10), integration barcodes (denoting a particular integration site) can be error corrected based on a minimum hamming distance and identical indels (referred to as alleles), and in the case where multiple alleles are associated with a given integration barcode a single allele is chosen based on the number of UMIs associated with it.

#### Calling independent clones

Collections of cells that are part of the same clonal population are identified by the set of integration barcodes each cell contains. Because all cells in the same clone are clonal, we reasoned that cells in the same clone should all share the same set of integration barcodes that the progenitor cell contained. Because of both technical artifacts (e.g., sequencing errors, PCR amplification errors) and biological artifacts (e.g., bursty expression, silenced regions) however, rather than looking for sets of non-overlapping sets, we perform an iterative clustering procedure. We begin by selecting the intBC that is shared amongst the most cells and assign any cell that contains this barcode to a cluster and remove these cells from the pool of unassigned cells. We perform this iteratively until at most *k* percent (in our case defined as.5*%* of cells are unassigned, which we assign to a “junk” clone.

Using the set of integration barcodes for each clone, we are able to identify doublets that consist of cells from different clones. Finally, after identifying doublets, to further filter out low-quality integration barcodes, for each clone integration barcodes that are not shared by at least 10% of cells in a given clone are filtered out, producing the final allele table.

#### Guidelines for final quality control

The thresholds discussed above are heuristic choices determined based on our hands-on experience with this type of target-site library processing. However, these thresholds will undoubtedly change depending on the sequencer used, the sequencing depth of the library, and the biological use case. For these reasons, we suggest that it is more effective to ensure that the final quality control numbers indicate that the library was processed sufficiently.

We present distributions for the metrics we find to be the most useful in Additional file 1: Fig S17: the UMIs per cellBC as a measure for how well sampled a cell is in (a), the reads per UMI as a measure for how confident one is of the UMI sequence in (b), UMIs per intBC as a measure for how confident one is of the called allele and intBC in (c), and a comparison of the number of UMIs versus the number of reads in (d), as a way of quickly assessing if there are any outlier UMIs.

Because this library was sequenced quite deeply, we do not expect typical applications to afford this degree of certainty. Instead, we suggest that cells should have at least 10 target-site UMIs, the reads/UMI distribution should have a mean at around 100–200 reads, and each intBC should have at least 5–10 UMIs associated with it. Cassiopeia’s processing pipeline creates figures for each of these statistics after filtering and close attention should be paid to these figures during the processing of the target-site sequencing data.

#### Filtering of clones for reconstruction

We filtered out clones upon two criteria: firstly, we removed clone 1 as we deduced that it had two defective guides; secondly, we removed lineages that reported fewer than 10% unique cells (thus removing clone 7). The remainder of clones were reconstructed.

#### Estimation of per-character mutation rates

To estimate mutation rates per clone, we assume that every target site was mutated at the same rate and independently of one another across 15 generations. Assuming some mutation rate, *p*, per character, we know that the probability of not observing a mutation in *d* generations is (1−*p*)^*d*^ in a given character and that the probability of observing at least 1 mutation in that character is 1−(1−*p*)^*d*^. Then, giving this probability 1−(1−*p*)^*d*^=*m* can be used as a probability of observing a mutated character in a cell and model the number of times a character appears mutated in a cell as a binomial distribution where the expectation is simply *nm* where *n* is the number of characters. Said simply, given this model, one would expect to see *nm* characters mutated in a cell. In this case, the empirical expectation is the mean number of times a given character appeared mutated in a cell (averaged across all cells), which we denote as *K* and propose that

$$K = nm = n * (1 - (1 - p)^{d}) $$

and thus *p*, the mutation rate, is

$$p = 1 - (1 - K/n)^{d} $$

### Bulk cutting experiment to determine prior probabilities of indel formation

Two and 4 days following triple-sgRNA (PCT61) infection, infected cells were selected by adding puromycin at 1.5 *μ*g/mL; puromycin-killed cells were removed by washing the plate with fresh medium. Cells were split every other day, and 500k cells were collected on days 7, 14, and 28. Frozen cell pellets were lysed, and the genomic DNA was extracted and purified by ethanol precipitation. The PCT48 Target Site locus was PCR amplified from genomic DNA samples (forward: TCGTCGGCAGCGTCAGATGTGTATAAGAGACAGAATCCAGCTAGCTGTGCAGC; reverse:GTCTCGTGGGCTCGGAGATGTGTATAAGAGACAGTCGAGGCTGATCAGCG) and further amplified to incorporate Illumina adapters and sample indices(forward:AATGATACGGCGACCACCGAGATCTACACXXXXXXXXTCGTCGGCAGCGTCAG;reverse:CAAGCAGAAGACGGCATACGAGATXXXXXXXXGTCTCGTGGGCTCGGAG; “X” denotes sample indices). The subsequent amplicon libraries were sequenced on an Illumina MiSeq (paired end, 300 cycles each). Sequencing data was analyzed as described below.

### Determining prior probabilities of indel formation

To determine the prior probabilities of edits, we leverage the fact that we have access to a large set of target sites (or intBCs) with a similar sequence (apart from the random barcode at the 5 ^′^ end); namely, a total of 117 intBC across the 11 clones. To compute the prior probability for a given indel, we compute the empirical frequency of observing this mutation out of all unique edits observed. Specifically, we compute the prior probability of a given indel *s*, *q*_*s*_ as the following:

$$q_{s} = \frac{f(s)}{|I|} $$

where *f*(*s*) is the number of intBCs that had *s* in at least one cell and |*I*| is the number of intBCs that are present in the dataset.

As further support for this method, we used the bulk experiment consisting of many separately engineered A549 cells, as described in the previous section. The advantage of the bulk experiment is that we have access to substantially more intBCs (> 10k), thus providing a more robust estimation of *q*_*s*_. We therefore employed the same approach to estimate indel formation rates from the bulk data and find that the resulting rates correlate well with the indel rates estimated from the single-cell lineage tracing experiment (Additional file 1: Fig S20).

### Doublet detection

#### Methods to detect doublets

We hypothesized that doublets could come in two forms and that we could use various components of the intBC data structure to identify them. Namely, doublets could be of cells from the identical clone, here dubbed “intra-doublets,” or doublets could be of cells from separate clones, here dubbed “inter-doublets.” In the case of “intra-doublets,” we can utilize the fact that these cells will have a large overlap in their set of intBCs but will report “conflicting” alleles for each of these intBCs. Thus, to identify these doublets, we calculate the percentage of UMIs that are conflicting in each cell. Explicitly, for each cell, we iterate over all intBCs and sum up the number of UMIs that correspond to an allele that conflicts with the more abundant allele for a given intBC; we then use the percentage of these UMIs to identify doublets. We perform this after all UMI and intBC correction in hopes of calling legitimate conflicts.

To deal with “inter-doublets,” we developed a classifier that leverages the fact that cells from different clones should have non-overlapping intBC sets. While this is the ideal scenario, often times intBCs are shared between clones for one of two reasons: (1) the clustering assignments are noisy or (2) the transfections of intBCs resulted in two cells receiving the same intBC, even though cells are supposed to be progenitors of separate clones. Our strategy is thus as follows: for each cell *c*_*i*_∈*C* calculate a “membership statistic,” *m*_*i*,*k*_ for each clone *l*_*k*_∈*L*. The membership statistic is defined as so:

$$m_{i,k} = \frac{\sum_{j \in I_{k}}\delta(i, j)p(j, k)}{\sum_{j \in I_{k}} (p(j, k))} $$

where *I*_*k*_ is the set of intBCs for the clone *l*_*k*_ and *p*(*j*,*k*) is the prevalence rate of the intBC *j* in *l*_*k*_. We use *δ*(*i*,*j*) as an indicator function for whether or not we observed the intBC *j* in the cell *c*_*i*_. Intuitively, this membership statistic is a weighted similarity for how well the cell fits into each clone, where we are weighting by how much we are able to trust the intBC that is observed in the cell. To put all on the same scale, we normalize by total membership per cell, resulting in our final statistic, $m^{\prime }_{i,k} = \frac {m_{i, k}}{\sum _{k'=0}^{k} m_{i, k'}}$. We then filter out doublets whose *m*^′^ for their classified clone falls below a certain threshold.

#### Simulation of doublets

We simulated two datasets to test our methods for identifying doublets and to find the optimal criterion on which to filter out doublets. To test this strategy, we took a single clone from our final Allele Table (the table relating all cells and their UMIs to clones) and formed 200 doublets by combining the UMIs from two cells. We generated 20 of these datasets and noted which cells were artificially introduced doublets.

Contrary to the strategy for simulating doublets from the same clone, we created artificial “inter” doublets from the final Allele Table by combining doublets from two different clones. Similarly, we generated 20 synthetic datasets each with 200 of these artificial doublets.

#### Identification of decision rule

To identify the optimal decision rule for calling both types of doublets, we tested decision rules ranging from 0 to 1.0 at 0.05 intervals and calculated the precision and recall at each of these rules. Taking these results altogether, we provide an optimal decision rule where the F-measure (or the weighted harmonic mean of the precision and recall) of these tests is maximal.

### Algorithmic approaches for phylogenetic reconstruction

One way to approach the phylogenetic inference problem is to view each target site as a “character” that can take on many different possible “states” (each state corresponding to an indel pattern induced by a CRISPR/Cas9 edit at the target site). Formally, these observations can be summarized in a “character matrix,” *M*∈*R*^*n*,*m*^, which relates the *n* cells by a set of characters *χ*={ *χ*_1_,..., *χ*_*m*_} where each character *χ*_*i*_ can take on some *k*_*i*_ possible states. Here, each sample, or cell, can be described as a concatenation of all of their states over characters in a “character string.” From this character matrix, the goal is to infer a tree (or phylogeny), where leaf nodes represent the observed cells, internal nodes represent ancestral cells, and edges represent a mutation event.

We first propose an adaption of a slow, but accurate, Steiner tree algorithm via integer lineage programming (ILP) to the lineage tracing phylogeny problem. Then, we propose a fast, heuristic-based greedy algorithm which simultaneously draws motivation from classical perfect phylogeny algorithms, and the fact that mutations can only occur unidirectionaly from the unmutated, or *s*_0_ state. Lastly, we combine these two methods and present a hybrid method, which presents better results than our greedy approach, yet remains feasible to run over tens of thousands of cells.

#### Adaptation to Steiner tree problem

Steiner trees are a general problem for solving for the minimum weight tree connecting a set of target nodes. For example, if given a graph *G*=(*V*,*E*) over some *V* vertices and *E* edges, finding the Steiner tree over all *v*∈*V* would amount to solving for the minimum spanning tree (MST) of *G*. While there exist polynomial time algorithms for the minimum spanning tree, the general Steiner tree problem, where the set of targets *T*⊆*V* is designated, is NP-hard.

Previously, Steiner trees have been suggested to solve for the maximum parsimony solution to the phylogeny problem. Here, the graph would consist of all possible cells (both observed and unobserved) and each edge would consist of a possible evolutionary event connecting two states (e.g., a mutation). Generally, given a set of length-*l* binary “character-strings” (recall that these are the concatenation of all character states for a given sample), we can solve for the maximum parsimony solution by finding the optimal Steiner tree over the 2^*l*^ hypercube (i.e., graph). As a result, by converting our multi-state characters to binary characters via one hot encoding, theoretically, we should be able to compute the most parsimonious tree which best explains the observed data. However, in practice, this method turns out to be infeasible, as we deal with hypercubes of size *O*(2^*mn*^), where *m* is the number of characters and *n* is the number of states. In the following, we will propose a method for estimating the underlying search space, providing us with a feasible solvable instance and a formulation of an integer linear programming (ILP) problem to solve for the optimal Steiner tree.

**Approximation of potential graph** We first begin by constructing a directed acyclic graph (DAG) *G*, where nodes represent cells. We then take the source nodes, or nodes with in-degree 0, of *G*, and for each pair of source nodes, consider the latest common ancestor (LCA) they could have had. This LCA has an unmutated state for character *χ*_*i*_ if they disagree across two source nodes, and the same state as the two source nodes if they agree in value. If the edit distance between these two cells is below a certain threshold *d*, we add the LCA to *G*, along with directed edges to the two source nodes, weighted by the edit distance between the parent and the source. We repeat this process until only one node remains as a source: the root.

One may think that this step explodes with *O*(*n*^2^) complexity at each stage, where *n* is the number of source nodes in each prior stage, as we consider all pairs of source nodes. However, we note that the number of mutations per latest common ancestor is always less than both children, and therefore, we eventually converge to the root. Therefore, when dealing with several hundred cells, the potential graph is feasible to calculate.

Furthermore, to add scalability to the approximation of the Potential Graph, we allow the user to provide a “maximum neighborhood size” which will be used to dynamically solve for the optimal LCA distance threshold *d* to use. One may think of this as the maximum memory or time allowed for optimizing a particular problem. Since the size of the Potential Graph can grow quite large in regard to the number of nodes, we iteratively create potential graphs for various threshold *d* and at each step ensure that the number of nodes in the network does not exceed the maximum neighborhood size provided. If at any point the number of nodes does exceed this maximum size, we return the potential graph inferred for an LCA threshold of *d*−1.

**Formulation of integer linear programming problem** Given our initial cells, *S*, the underlying potential graph drawn from such cells, *G*, and the final source node, or root, *r* from *G*, we are interested in solving for $\mathcal {T} = SteinerTree(r, S, G)$. We apply an integer linear programming (ILP) formulation of Steiner tree, formulated in terms of network flows, with each demand being met by a flow from source to target. Below, we present the integer linear programming formulation for Steiner tree. We use Gurobi [61], a standard ILP solver package

$${\begin{aligned} & \text{minimize} & \sum_{(u,v) \in E} d_{uv}^{b} \cdot w(u,v) \\ & \text{subject to} & \sum_{(u,v) \in E} d_{uv} - \sum_{(v,w) \in E} d_{vw} &= 0 && \forall v \!\notin\! S \cup \{r\} \\ & & \sum_{(r, w) \in E} d_{rw} &= -|S| && \\ \end{aligned}} $$

$${\begin{aligned} & & \sum_{(u,s) \in E} d_{us} &= 1 && \forall s \in S \\ & & d_{uv}^{b} &\geq \frac{d_{uv}}{|S|} && \forall (u,v) \in E\\ & & d_{uv} &\in \{0,..,|S|\} && \forall (u,v) \in E\\ & & d_{uv}^{b} &\in \{0,..,1\} && \forall (u,v) \in E \end{aligned}} $$

Each variable *d*_*uv*_ denotes the flow through edge (*u*,*v*), if it exists; each variable $d_{uv}^{b}$ denotes whether (*u*,*v*) is ultimately in the chosen solution sub-graph. The first constraint enforces flow conservation, and hence that the demands are satisfied, at all nodes and all conditions. The second constraint requires |*S*| units of flow come out from the *root*. The third constraint requires that each target absorb exactly one unit of flow. The fourth constraint ensures that if an edge is used at any condition, it is chosen as part of the solution.

Below, we explicitly define the algorithm in pseudocode.


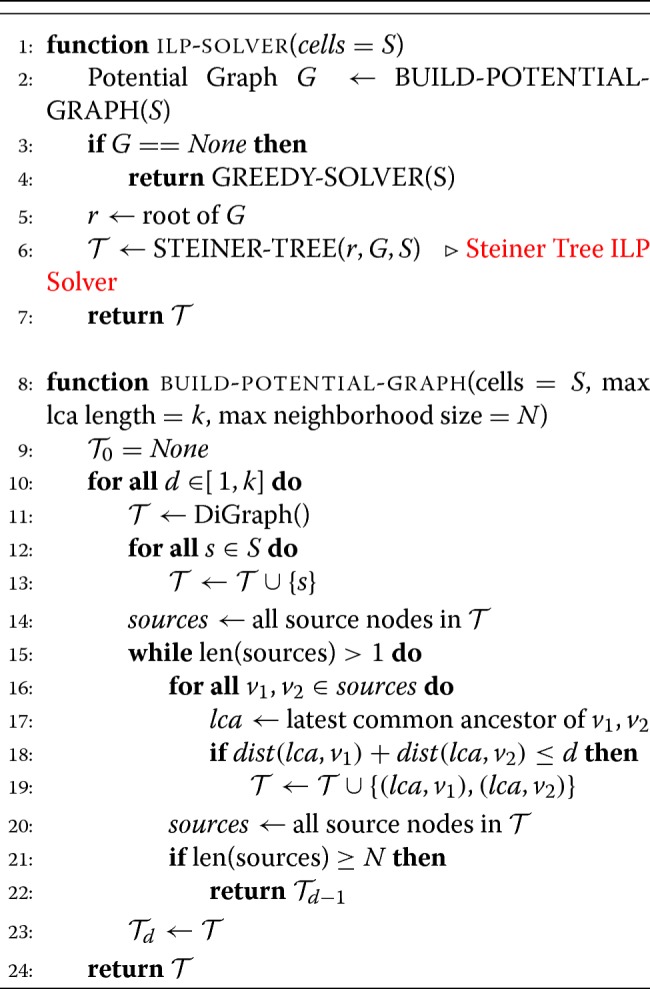


#### Stability analysis of the maximum neighborhood size parameter

To evaluate the stability of the user-defined maximum neighborhood size parameter, we assessed the accuracy of the reconstructions for parameters varying from 800 to 14,000. We used trees simulated under default conditions (400 samples, 40 characters, 40 states per character, 11 generations, 2.5*%* mutation rate per character, and a mean dropout rate of 17%). The accuracy of trees were compared to the tree generated with a parameter of 14,000 using the triplets correct statistic. We used 10 replicates to provide a sense for how stable a given accuracy is.

In addition to providing measures of accuracy, we also provide the optimal LCA threshold *d* found for a given maximum neighborhood size during the inference of these potential graphs. Using these analysis, we found that a maximum neighborhood size of 10,000 nodes seemed to be an ideal tradeoff between scalability and accuracy (as it is in the regime where accuracy saturates) for our default simulations. This corresponded to a mean LCA threshold, *d*, of approximately 5.

#### Heuristic-based greedy method

**On perfect phylogeny and single-cell lineage tracing** In the simplest case of phylogenetics, each character is binary (i.e., *k*_*i*_=2,∀*i*∈*m*) and can mutate at most once. This case is known as “perfect phylogeny” and there exist algorithms (e.g., a greedy algorithm by Dan Gusfield [30]) for identifying if a perfect phylogeny exists over such cells, and if so find one efficiently in time *O*(*mn*), where *m* is the number of characters and *n* are the number of cells. However, several limitations exist with methods such as Gusfield’s algorithm. One potential problem in using existing greedy perfect phylogeny algorithms for lineage tracing is that they require the characters to be binary. Indeed, if the characters are allowed to take any arbitrary number of states, the perfect phylogeny problem becomes NP-hard. However, while the number of states (CRISPR/Cas9-induced indels at a certain target site) in lineage tracing data can be large, these data benefit from an additional restriction that makes it more amenable for analysis with a greedy algorithm. Below, we show that because the founder cell (root of the phylogeny) is unedited (i.e., includes only uncut target sites) and that the mutational process is irreversible, we are able to theoretically reduce the multi-state instance (as observed in lineage tracing) to a binary one so that it can be resolved using a greedy algorithm.

A second remaining problem in using these perfect phylogeny approaches is that we cannot necessarily expect every mutation to occur exactly once. In theory, it may happen that the same indel pattern is induced in exactly the same target site on two separate occasions throughout a lineage tracing experiment, especially if a large number of cell cycles takes place. A final complicating factor is that these existing greedy algorithms often assume that all character states are known, whereas lineage tracing data is generated by single-cell sequencing, which often suffers from limited sensitivity and an abundance of “dropout” (stochastic missing data) events.

**The greedy algorithm** We suggest a simple heuristic for a greedy method to solve the maximum parsimony phylogeny problem, motivated by the classical solution to the perfect phylogeny problem and irreversibility of mutation. Namely, we consider the following method for building the phylogeny: Given a set of cells, build a tree top-down by splitting the cells into two subsets over the most frequent mutation. Repeat this process recursively on both subsets until only one sample remains.

Formally, we choose to split the dataset into two subsets, *O*_*i*,*j*_ and $\overline {O}_{i,j}$, such that *O*_*i*,*j*_ contains cells carrying mutation *s*_*j*_ in *χ*_*i*_, and $\overline {O}_{i,j}$ contains cells without *s*_*j*_ in *χ*_*i*_. We choose *i*,*j* based on the following criteria:

$$i,j = \underset{i,j}{\text{argmax}}\, n_{i,j} $$

where *n*_*i*,*j*_ is the number of cells that carry mutation *s*_*j*_ in character *χ*_*i*_. We continue this process recursively until only one sample exists in each subset. We note that this method operates over cells with non-binary states, solving the first of problems addressed earlier.

A major caveat exists with methods such as the greedy method proposed by Gusfield, as well as the one proposed by us thus far: namely, they assume all character states are known (i.e., no dropout). However, in our practice, we often encounter dropout as a consequence of Cas9 cutting or stochastic, technical dropout due to the droplet-based scRNA-seq platform. To address this problem in our greedy approach, during the split stage, these cells are not initially assigned to either of the two subsets, *O*_*i*,*j*_ or $\overline {O}_{i,j}$. Instead, for each individual sample which contains a dropped out value for chosen split character *χ*_*i*_, we calculate the average percentage of mutated states shared with all other cells in *O*_*i*,*j*_ and $\overline {O}_{i,j}$ respectively, and assign the sample to the subset with greater average value.

Appending the dropout resolution stage with the initial split stage, we present our greedy algorithm below in its entirety.


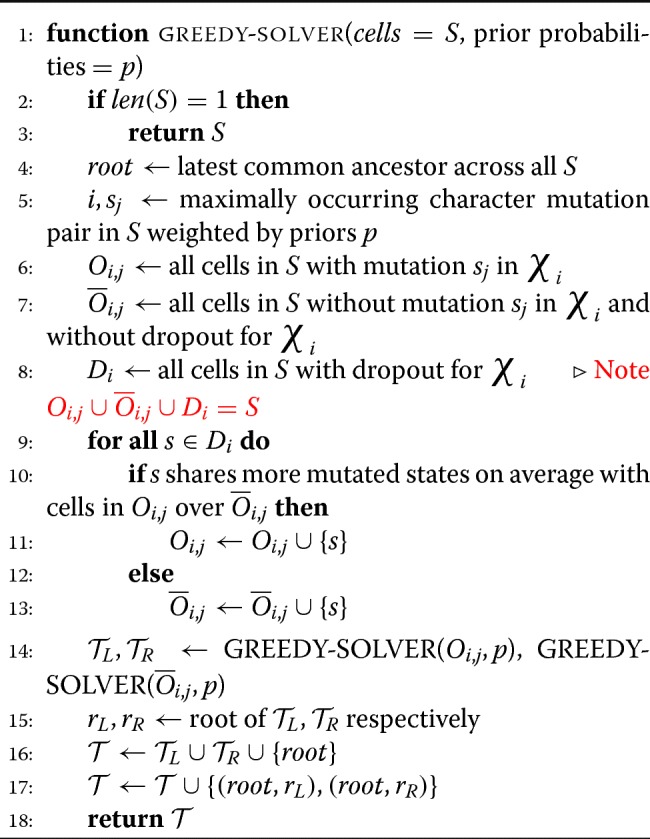


Overall, this method is very efficient and scales well into tens of thousands of cells. Below, we show via proof that this algorithm can find perfect phylogeny if one exists.

**Cassiopeia-Greedy algorithm can solve multi-state perfect phylogeny** Here we show that while not required, Cassiopeia can solve the multi-state perfect phylogeny problem optimally. Importantly, however, Cassiopeia’s effectiveness makes no assumption about perfect phylogeny existing in the dataset but rather leverages this concept to provide a heuristic for scaling into larger datasets.

To show how Cassiopeia’s greedy method can solve perfect phylogeny optimally, we begin by introducing a few clarifying definitions prior to the main theorem. We define *M* as the original *n* cells by *n* character *k*-state matrix (i.e., entries $\in \{s_{0},\dots,s_{k-1}\}$). We say *M* has a zero root perfect phylogeny if there exists a tree $\mathcal {T}$ over its elements and character extensions such that the state of the root is all zeros and every character state is mutated into at most once. In addition, we assume that all non-leaf nodes of $\mathcal {T}$ have at least two children (i.e., if they only have one child, collapse two nodes into one node). Finally, we offer a definition for *character compatibility*:

##### **Definition 1**

(*Character Compatibility*). For a pair of binary characters, (*χ*_1_, *χ*_2_), where the sets (*O*_1_,*O*_2_) contain the sets of cells mutated for *χ*_1_ and *χ*_2_, respectively, we say that they are compatible if one of the following is true:

- *O*_1_⊆*O*_2_
- *O*_2_⊆*O*_1_
- *O*_1_∩*O*_2_=*∅*

This definition extends to multi-state characters as well, assuming they can be binarized.

Before proving the main theorem, we first prove the following lemma:

##### **Lemma 1**

If *M* has a perfect phylogeny, then the most frequent character, mutation pair appears on an edge from the root to a direct child node.

##### *Proof*

WLOG let $\text {\Large \(\chi \)}_{i}: s_{0} \rightarrow s_{j}$ denote the maximally occurring character, mutation pair within *M*. Suppose by contradiction that this mutation does not appear on an edge directly from root to a child, but rather on some edge (*u*,*v*) that is part of a sub-tree whose root *r*^∗^, is a direct child of the root. As *r*^∗^ has at least two children, this implies that the mutation captured from the root to *r*^∗^ must be shared by strictly more cells than $\text {\Large \(\chi \)}_{i}: s_{0} \rightarrow s_{j}$, thereby reaching a contradiction on $\text {\Large \(\chi \)}_{i}: s_{0} \rightarrow s_{j}$ being the maximally occurring mutation. □

##### **Theorem 1**

The greedy algorithm accurately constructs a perfect phylogeny over *M* if one exists.

##### *Proof*

We approach via proof by induction. As a base case, a single is trivially a perfect phylogeny over itself.

Now suppose by induction that for up to *n*−1 cells, if there exists a perfect phylogeny $\mathcal {T}$ over such cells, then the greedy algorithm correctly returns the perfect phylogeny. Consider the case of *n* cells. By the above lemma, we know we can separate these *n* cells into two subsets based on the most frequent character, mutation pair $\text {\Large \(\chi \)}_{i}: s_{0} \rightarrow s_{j}$, *O*_*i*,*j*_ and $\overline {O}_{i,j}$, where *O*_*i*,*j*_ contains cells with mutation *s*_*j*_ over *χ*_*i*_, and $\overline {O}_{i,j}$ = *M*−*O*_*i*,*j*_. By induction, the greedy algorithm correctly returns two perfect phylogenies over *O*_*i*,*j*_ and $\overline {O}_{i,j}$, which we can merge at the root, giving us a perfect phylogeny over *n* cells. □

#### Accounting for prior probability of mutations

In most situations, the probability of mutation to each distinct state may not be uniform (i.e., character *χ*_1_ mutating from the unmutated state *s*_0_ to state *s*_4_ may be twice as likely as mutating to state *s*_6_). Therefore, we incorporate this information into choosing which character and mutation to split over based on the following criteria:

$$i,j = \underset{i,j}{\text{argmin}}\, p_{i}(s_{0}, s_{j})^{f(n_{i,j})}$$

where *p*_*i*_(*s*_0_,*s*_*j*_) is the probability that character *χ*_*i*_ mutates from the unmutated state *s*_0_ to *s*_*j*_ and *f*(*n*_*i*,*j*_) is some transformation of the number of cells that report mutation *j* in character *i* that is supposed to reflect the future penalty (number of independent mutations of character *i* to state *j*) we will have to include in the tree if we do not pick *i*,*j* as our next split. After a comparison of 5 different transformations (Additional file 1: Fig S15), we find that *f*(*n*_*i*,*j*_)=*n*_*i*,*j*_ gives the best performance, leaving us with the following criteria for splittings:

$$i,j = \underset{i,j}{\text{argmin}}\, p_{i}(s_{0}, s_{j})^{n_{i,j}}$$

#### A hybrid method for solving single-cell lineage tracing phylogenies

Due to the runtime constraints of the Steiner tree method, it is infeasible for such method to scale to tens of thousand of cells. Therefore, we build a simple hybrid method which takes advantage of the heuristic proposed in the greedy algorithm and the theoretical optimality of the Steiner tree method. Recall that in the greedy method, we continued to choose splits recursively until only one sample was left per subset. In this method, rather than follow the same process, we choose a cutoff for each subset (e.g., 200 cells). Once a subset has reached a size lower than the said cutoff, we feed each individual subset into the Potential Graph Builder and Steiner tree solver, which compute an optimal phylogeny for the subset of cells. After an optimal sub-tree is found, we merge it back into the greedy tree. Therefore, we build a graph whose initial mutations are chosen from the greedy method and whose latter mutations are chosen more precisely via the Steiner tree approach.

Below, we present a pseudocode algorithm for the hybrid method. We note the slight difference in greedy from before. Namely, greedy additionally accepts a cutoff parameter and, in addition to returning a network built up to that cutoff, returns all subsets that are still needed to be solved.


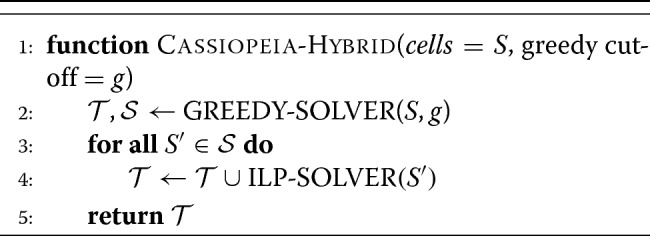


This approach scales well when each instance of Steiner tree is ran on an individual thread, and thus often takes only a few hours to run on several thousand cells.

### Theoretical analysis of parallel evolution

#### Estimating first and second moments of double mutations

**Expected number of double mutations** Under the framework of our simulation, we assume that each at each generation, every cell divides, and then each character of each cell undergoes random mutation independently. Let *p* be the probability that a particular character mutates and *q* be the probability the character took on a particular mutated state given that it mutated. Let *T* be the true phylogenetic tree over the samples. According to our model, *T* must be a full binary tree, and the samples are leaves of *T*. Let *X* be the total number of times a particular mutation occurred in the *T*. Let *X*_*u*,*v*_ be an indicator variable for edge (*u*,*v*) such that:

$$X_{u,v} = \left\{ \begin{array}{ll} 1 & \text{if a mutation occurs on edge }(u,v)\\ 0 & \text{otherwise} \end{array}\right. $$

Let *h* be the height of the *T*, which is equalled to the number of generations. If *v* is at depth *d* in *T*, then the probability that a mutation occurs at (*u*,*v*) is *pq*(1−*p*)^*d*−1^. Since there are 2^*d*^ nodes at depth *d*, we have:

1 $$ \begin{aligned} E(X) &= \sum_{(u,v)\in T}E(X_{u,v})\\ &= \sum_{d = 1}^{h}2^{d}pq(1-p)^{d-1} \\ &= \frac{2pq((2-2p)^{h}-1)}{1-2p} \end{aligned}  $$

Let *n*=2^*h*^ be the number of cells in our sample. If *p*>0.5, *E*(*X*)≤2*pq*/(2*p*−1), if *p*=0.5, *E*(*X*)=2*pqh*=*O*(log*n*), and if *p*<0.5, $E(X) = O(n^{\frac {1}{\log _{2}{2-2p}}})$. Moreover, for fixed *h*, *E*(*X*) has a single peak for *p*∈[0,1], meaning that it increases with *p* for sufficiently small values of *p*, and always increases with *q*. Intuitively, this is because *E*(*X*) is small if (1) *p* is small enough that the character never mutates much throughout the experiment or (2) *p* is large enough that most mutations occur near the top of the tree, resulting in the extinction of unmutated cells early in the experiment. While *E*(*X*) peaks for values of *p* in between, it is always directly proportional to *q* because *X* is simply equalled to *q* time the number of times the character mutated.

**Variance of double mutations** We can compute the variance as:

$$\begin{array}{*{20}l} Var(X) &= E(X^{2}) - E(X)^{2}\\ &= 2\sum_{(u,v)\neq(u',v')}E(X_{u,v}X_{u',v'}) + E(X) - E(X)^{2} \end{array} $$

To compute $\phantom {\dot {i}\!}E(X_{u,v}X_{u',v'})$, we note that for a given pair of edges (*u*,*v*) and (*u*^′^,*v*^′^), such that *LCA*(*u*,*u*^′^) is at depth *d*, *u* is at depth *d*+*l*, and *u*^′^ is at depth *l*+*k*, the probability that a mutation occurred on both edges is *p*^2^*q*^2^(1−*p*)^*d*+*l*+*k*^. Thus, we have:

$${\begin{aligned} \sum_{(u,v)\neq(u',v')}E(X_{u,v}X_{u',v'}) &= \sum_{d=0}^{h-1}2^{d}\sum_{k=0}^{h-d-1}\sum_{l=0}^{h-d-1}2^{l+k}p^{2}q^{2}(1-p)^{d+l+k}\\ &= p^{2}q^{2}\sum_{d=0}^{h-1}(2-2p)^{d}(\sum_{k=0}^{h-d-1}(2-2p)^{k})^{2}\\ &= \frac{p^{2}q^{2}}{(2p-1)^{2}}\sum_{d=0}^{h-1}(2p-2)^{d}((2p-2)^{(h-d)}-1)^{2}\\ &\leq \frac{p^{2}q^{2}}{(2p-1)^{2}}\sum_{d=0}^{h-1}(2p-2)^{2h-d}\\ &= (2p-2)^{h+1}\frac{p^{2}q^{2}}{(2p-1)^{2}}\sum_{d=0}^{h-1}(2p-2)^{d}\\ &\leq \frac{p^{2}q^{2}(2p-2)^{2h+1}}{(2p-1)^{3}} \end{aligned}} $$

Thus, we can bound the variance as follows:

2 $$ {\begin{aligned} Var(X) &\leq \frac{2p^{2}q^{2}(2p-2)^{2h+1}}{(2p-1)^{3}} + \frac{2pq\left(1-(2-2p)^{h}\right)}{2p-1} \\&\quad- \frac{4p^{2}q^{2}(1-(2-2p)^{h})^{2}}{(2p-1)^{2}} \end{aligned}}  $$

This means that in the case that *p*>0.5:

$$Var(X) \leq \frac{2p^{2}q^{2}}{(2p-1)^{3}} + \frac{2pq}{2p-1} - \frac{4p^{2}q^{2}}{(2p-1)^{2}}$$

In the case that *p*=0.5:

$$Var(X) = O(h^{3}) = O(\log^{3}(n))$$

In the case that *p*<0.5:

$$Var(X) = O(n^{\frac{2}{\log_{2}{2-2p}}})$$

#### Least squares linear estimate and negative correlation between frequency and the number of double mutations

To justify the greedy, we must show that if a mutation occurs frequently, then it is likely to have occurred less times throughout the experiment. Let *Y* be the frequency of a particular mutation in the samples. We estimate *X* given *Y* using the least squares linear estimate (LLSE) as follows:

3 $$ L(X|Y) = E(X) + \frac{CoV(X,Y)}{Var(Y)}(Y-E(Y))  $$

Since *CoV*(*X*,*Y*)=*E*(*XY*)−*E*(*X*)*E*(*Y*), we need only to compute *E*(*XY*), which we do by expressing *X* and *Y* in terms of the same indicators:

$$Y = \frac{1}{2^{h}}\sum_{(u,v) \in T} 2^{\text{depth}(v)}X_{u,v}$$

As a sanity check, it can easily be verified that *E*(*Y*)=*q*(1−(1−*p*)^*h*^) by computing *E*(*Y*) using these indicators:

$$\begin{array}{*{20}l} E(Y)&=2^{-h}\sum_{d=1}^{h}2^{d}(1-p)^{d-1}pq*2^{h-d}\\ &=pq\sum_{d=1}^{h}(1-p)^{d-1}\\ &=q(1-(1-p)^{h})\\ \end{array} $$

Thus, we can compute *E*(*XY*) similar to how we computed *E*(*X*^2^) for variance.

4 $$ {\begin{aligned} E(XY) &= 2^{-h}E((\sum_{(u,v)\in T}X_{u,v})(\sum_{(u,v)\in T}2^{\text{depth}(v)}X_{u,v}))\\ &=2^{-h}\Big(2\sum_{(u,v)\neq (u',v')}2^{\text{depth}(v)}E(X_{u,v}X_{u',v'})+\sum_{(u,v)\in T}2^{\text{depth}(v)}E(X_{u,v}^{2})\Big)\\ &= 2*2^{-h}\sum_{d=0}^{h-1}2^{d}\sum_{k=0}^{h-1}\sum_{l=0}^{h-1}2^{l+k}p^{2}q^{2}(1-p)^{d+l+k}*2^{h-d-l-1} + E(Y)\\ &= p^{2}q^{2}\sum_{d=0}^{h-1}\sum_{k=1}^{h-d-1}\sum_{l=0}^{h-d-1}(1-p)^{d}(2-2p)^{k}(1-p)^{l} + E(Y)\\ &=\frac{pq^{2}}{1-2p}\sum_{d=0}^{h-1}(2-2p)^{h-d}-1)(1-(1-p)^{h-d})(1-p)^{d} + E(Y)\\ &=\frac{pq^{2}}{1-2p}\Big(2(2-2p)^{h}(1-2^{-h})-\frac{(2-2p)(1-p)^{h}((2-2p)^{h}-1)}{1-2p} \\ & \quad \quad \quad \quad - \frac{1-(1-p)^{h}}{p}+h(1-p)^{h}\Big) + E(Y)\\ \end{aligned}}  $$

Assuming that is $p < 1-1/\sqrt {2} \approx 0.29$ (based on our estimation of Cas9-cutting rates, this seems to be a biologically relevant probability), we have:

$$\begin{array}{*{20}l} {\lim}_{h\rightarrow \infty}CoV(X,Y) &= \Big(2-\frac{2-2p}{1-2p}\Big)\frac{pq^{2}(2(1-p)^{2})^{h}}{1-2p}\\ &= -\infty \end{array} $$

since 2<(2−2*p*)/(1−2*p*) when *p*<0.5. *Var*(*Y*) can be computed using the same indicators:

5 $$ {\begin{aligned} Var(Y) &= 2\sum_{i,j}E(Y_{i}Y_{j}) + \sum_{i}E(Y_{i}^{2}) - E(Y)^{2}\\ \sum_{i,j}E(Y_{i}Y_{j}) &= 2^{-2h}\sum_{d=0}^{h-1}2^{d}(1-p)^{d}(\sum_{k=0}^{h-d-1}2^{k}(1-p)^{k}pq*2^{h-d-k-1})^{2}\\ &=\frac{q^{2}}{4}\sum_{d=0}^{h-1}(\frac{1-p}{2})^{d}(\frac{1-(1-p)^{h-d}}{p})^{2}\\ &=\frac{q^{2}}{4}\sum_{d=0}^{h-1}(\frac{1-p}{2})^{d} - \frac{2(1-p)^{h}}{2^{d}}+ \frac{(1-p)^{2h}}{(2-2p)^{d}}\\ &= \frac{q^{2}}{4}\Big(\frac{2(1-(\frac{1-p}{2})^{h})}{1+p}-4(1-p)^{h}(1-2^{-h}) + \\ & \quad \quad \quad \quad \quad \frac{(2-2p)(1-p)^{2h}(1-(\frac{1}{2-2p})^{h})}{1-2p}\Big) \end{aligned}}  $$

$$\begin{array}{*{20}l} \sum_{i}E(Y_{i}^{2}) &= 2^{-2h}\sum_{d=1}^{h}2^{d}(1-p)^{d-1}pq*2^{2(h-d)}\\ &=\frac{pq}{2}\sum_{d=0}^{h-1}(\frac{(1-p)}{2})^{d}\\ &=\frac{pq(1-(\frac{1-p}{2})^{h})}{1+p} \end{array} $$

Note that if *p*<0.5, every term in *Var*(*Y*) converges to a constant as *h*→*∞*. Thus, if (1−*p*)^2^>0.5, then as the depth increases, *X* and *Y* become exponentially more negatively correlated. This means that for biologically relevant values of *p*, the frequency of a mutation in the samples is negatively correlated with the number of times the mutation occurred, thus justifying the rationale of splitting the sample on more frequently occurring mutations.

#### Simulation for tracking the evolution of a particular mutation

To more efficiently simulate the number of occurrences of a particular mutation, we define {*N*_1_,*N*_2_,...*N*_*h*_} as a Markov chain, where *N*_*t*_ is the number of unmutated cells at generation *t*, and *N*_1_=1. Let *A*_*t*_∼*Bin*(2*N*_*t*_,*p*) be the number of cells that mutates at generation *t* and *B*_*t*_∼*Bin*(*A*_*t*_,*a*) be the number of mutated cell that took on the particular state in question. The Markov chain evolves as *N*_*t*+1_=2*N*_*t*_−*A*_*t*_. Note that we assume, in this model, that mutation can only occur after cell division. Thus, we have $X = \sum _{t=1}^{h}B_{t}$ and $Y = \sum _{t=1}^{h}2^{t-h}B_{t}$.

### Assessing the precision of greedy splits

To assess the precision of greedy splits, we first simulated 100 true phylogenies of 400 cells (without dropout) for all pairs of parameters in *num*_*states*={2,10,40} and *p*_*c*_*ut*={0.025,0.1,0.4}. For each network, we assessed the precision of the greedy split as follows:

1. We used the criteria *i*,*j*= arg max_*i*,*j*_*n*_*i*,*j*_ to select the character *χ*_*i*_ and state *j* to split on (as Cassiopeia-Greedy would do). This group of cells that have a mutation *j* in character *χ*_*i*_ is called *G*.
2. For defining the a set of *n* subsets corresponding to cells that inherited the (character, state) pair (*i*,*j*) independently using the true phylogenies, and call this set *S*=(*s*_1_,*s*_2_,...,*s*_*n*_) (this corresponds to there being *n* parallel evolution events for the (character, state) pair (*i*,*j*).
3. We presume that the largest group of cells in *S* is the “true positive” set (let this be defined as *s*^′^= arg max_*s*_|*s*_*i*_|). We then define the precision *P* as the proportion of true positives in the set *G*—i.e., $P = \frac {|s'|}{|G|}$.

### Statistics for IVLT analysis

#### Meta purity statistic

To calculate the agreement between clades (i.e., the leaves below a certain internal node of the tree) and some meta value, such as the experimental plate from which a sample came from, we can employ a chi-squared test. Specifically, we can compute the following statistic: considering some *M* clades at an arbitrary depth *d*, we find the count of meta values associated with each leaf in each clade, resulting in a vector of values *m*_*i*_ comprised of these meta-counts for each clade *i*. We can form a contingency table summarizing these results, *T*, where each internal value is exactly *m*_*i*,*j*_—the counts of the meta item *j* in clade *i*. A chi-squared test statistic can be computed from this table.

To compare across different trees solved with different methods, we report the chi-squared test statistic as a function of the number of clades, or degrees of freedom of the test.

#### Mean majority vote statistic

The mean majority vote statistic seeks to quantify how coherent each clade is with respect to its majority vote sample at a give depth. For a given clade with leaves *L*_*i*_ where |*L*_*i*_|=*n*, where every leaf *l*_*i*,*j*_ corresponds to cell *j* in clade *i* has some meta label *m*_*j*_, the majority vote of the clade is $v = argmax_{m' \in M} \sum _{j \in n} \delta (j, m')$. Here, *M* is the full set of possible meta values and *δ*(*m*_*j*_,*m*^′^) is an indicator function evaluating to 1 iff *m*_*j*_=*m*^′^. The membership of this clade is then simply $\frac {\sum _{j \in n} \delta (m_{j}, v)}{n}$. Then, the mean membership is the mean of these membership statistics for all clades at a certain depth (i.e., if the tree was cut at a depth of *d*, the clades considered here are all the internal nodes at depth *d* from the root). By definition, this value ranges from $\frac {1}{|M|}$ to 1.0.

As above, to compare across different trees solved with various methods, we report this mean membership statistic as a function of the number of clades.

### Triplets correct statistic

To compare the similarity of simulated trees to reconstructed trees, we take an approach which compares the sub-trees formed between triplets of the terminal states across the two trees. To do this, we sample ∼10,000 triplets from our simulated tree and compare the relative orderings of each triplet to the reconstructed tree. We say a triplet is “correct” if the orderings of the three terminal states are conserved across both trees. This approach is different from other tree comparison statistics, such as Robinson-Foulds [34], which measures the number of edges that are similar between two trees.

To mitigate the effect of disproportionately sampling triplets relatively close to the root of the tree, we calculate the percentage of triplets correct across each depth within the tree independently (depth measured by the distance from the root to the latest common ancestor (LCA) of the triplet). We then take the *average* of the percentage triplets correct across all depths. To further reduce the bias towards the few triplets that are sampled at levels of the tree with very few cells (i.e., few possible triplets), we modify this statistic to only take into account depths where there at least 20 cells. We report these statistics without this depth threshold in Additional file 1: Fig S8.

### Allelic and phylogenetic distances

For the analysis in Fig. 4, we define two metrics to capture cell-to-cell similarity: a normalized allelic distance and normalized phylogenetic distance. The normalized allelic distance is calculated as follows: for all target sites *χ*_*m*_∈{ *χ*_1_,..., *χ*_*M*_} in a pair of cells *c*_*i*_ and *c*_*j*_:

1. if state in *χ*_*m*_ is the same in *c*_*i*_ and *c*_*j*_, continue
2. else if state in *χ*_*m*_ is 0 or missing in either *c*_*i*_ or *c*_*j*_ increment the allelic distance by 1
3. else increment the allelic distance by 2

Finally, the allelic distance for a pair of cells is normalized by 2∗*M*, where *M* is the number of target sites.

The phylogenetic distance is defined as simply the number of mutations separating the two cells *c*_*i*_ and *c*_*j*_ as implied by the tree (i.e., the number of mutations along the branches for the shortest path separating *c*_*i*_ and *c*_*j*_). The normalized phylogenetic distances is this distance, divided by the diameter (defined as the maximum phylogenetic distance between all pairs of cells) of the tree.

### Bootstrapping analysis

Bootstrapping was done using a custom function for sampling *M* target sites (i.e., characters) from an *N*×*M* character matrix with replacement and reconstructing trees from these bootstrapped samples. After performing tree inference, we collapsed “singles” using the collapse.singles function in the R package “ape.” For the purposes of our robustness analysis, we sampled *B*=100 trees from *N*=10 simulated trees and used the Transfer Bootstrap Expectation (TBE) [62] statistic for assessing branch support for each clade as implemented in Booster (available at https://github.com/evolbioinfo/booster/).

### Application of Camin-Sokal

We applied Camin-Sokal using the “mix” program in PHYLIP [63] as done for reconstructions for McKenna et al. [5] and Raj et al. [6]. To use “mix,” we first factorized the characters into binary ones (thus ending up with $\sum _{i} s_{i}$ binary characters total, where *s*_*i*_ is the number of states that character *i* presented). Then, we one-hot encoded the states into this binary representation where every position in the binary string represented a unique state at that character. We thus encoded every cell as having a 1 in the position of each binary factorization corresponding to the state observed at that character. If the cell was missing a value for character *i*, the binary factorization of the character was a series of “?” values (which represent missing values in PHYLIP “mix”) of length *s*_*i*_. Before performing tree inference, we weighted every character based on the frequency of non-zero (and non-missing values) observed in the character matrix. After PHYLIP “mix” found a series of candidate trees, we applied PHYLIP “consense” to calculate a consensus tree to then use downstream.

### Application of neighbor joining

We used Biopython’s neighbor-joining procedure to perform all neighbor joining in this manuscript. We begun similarly to the Camin-Sokal workflow, first factorizing all of the characters into a binary representation. Then, we applied the neighbor-joining procedure using the “identity” option as our similarity map.

### Application of Cassiopeia

#### Reconstruction of simulated data

We used Cassiopeia-ILP with a maximum neighborhood size of 10,000 and time to converge of 12,600s. Cassiopeia-Hybrid used a greedy cutoff of 200, a maximum neighborhood size of 6000 and 5000 s to converge. Cassiopeia-Greedy required no additional hyperparameters. Simulations with priors applied the exact prior probabilities used to generate the simulated trees.

#### Reconstruction of in vitro clones

For both Cassiopeia-Hybrid with and without priors, we used a cutoff of 200 cells and each instance of Cassiopeia-ILP was allowed 12,600 s to converge on a maximum neighborhood size of 10,000. Cassiopeia-ILP was applied with a maximum neighborhood size of 10,000 and a time to converge of 12,600 s.

#### Simulation of target-site sequences for alignment benchmarking

To determine an optimal alignment strategy and parameters for our target-site sequence processing pipeline, we simulated sequences and performed a grid search using Emboss’s Water algorithm (a local alignment strategy). We simulated 5000 sequences. For each sequence, we begun with the reference sequence and subjected it to multiple rounds of mutagenesis determined by a Poisson distribution with *λ*=3, and a maximum of 5 cuts. During each “cutting” event, we determined the outcomes as follows:

1. Determine the number of Cas9 proteins localizing to the target site in this iteration, where *n*_*cas*9_∼min(3,*Pois*(*λ*=0.4)).
2. Determine the site(s) to be cut by choosing available sites randomly, where the probability of being chosen is $p = \frac {1}{n_{\text {uncut}}}$ and *n*_uncut_ is the number of sites uncut on that sequence.
3. If *n*_*cas*9_=1, we determined the type of the indel by drawing from a Bernoulli distribution with a probability of success of 0.75 (in our case, a “success” meant a deletion and a “failure” meant an insertion). We then determined by drawing from a negative binomial distribution as so: *s*∼min(30,max(1, *NB*(0.5,0.1))). In the case of an insertion, we added random nucleotides of size *s* to the cut site, else we removed *s* nucleotides.
4. In the case of *n*_*cas*9_≥2, we performed a resection event where all nucleotides between the two cut sites selected were removed.
5. After a cut event, we appended the result of the Cas9 interaction to a corresponding CIGAR string

Our Water simulations were exactly 300 bp, possibly extending past the Poly-A signal, as would be the case reading off a Nova-seq sequencer.

Upon simulating our ground truth dataset, we performed our grid search by constructing alignments with Water with a combination of gap open and gap extension penalties. We varied the gap open penalties between 5 and 50 and gap extension penalties between 0.02 and 2.02.

To score resulting alignments, we compared the resulting CIGAR string to our ground truth CIGAR string for each simulated sequence. To do so, we first split each cigar string into “chunks,” corresponding to the individual deletions or insertions called. For example, for some CIGAR string 40*M*2*I*3*D*10*M*, the chunks would be 40*M*, 2*I*, 3*D*, and 10*M*. Then, beginning with a max score of 1, we first deducted the difference between the number of chunks in the ground truth and the alignment. Then, in the case where the number of chunks were equal between ground truth and alignment, we deducted the percent nucleotides that differed between CIGARs. For example, if the ground truth was 100*M* and the alignment gave 95*M*, the penalty would be 0.05.

To find the optimal set of parameters, we selected a parameter pair that not only scored very well, but also located in the parameter space where small perturbations in gap open and gap extension had little effect.

#### Simulation of lineages for algorithm benchmarking

We simulated lineages using the following parameters:

1. The number of characters to consider, *C*
2. The number of states per character, *S*
3. The dropout per characters, *d*_*c*_ ∀*c*∈*C*
4. The depth of the tree (i.e., the number of binary cell division), *D*
5. The probability that a site can be mutated, *p*. This is a general probability of cutting
6. The rate at which to subsample the data at the end of the experiment, *M*

To simulate the tree, we begin by first generating the probability of each character mutating to a state, here represented as *p*_*c*_(0,*s*),∀*s*∈*S*. In order to do this, we fit a spline function to the inferred prior probabilities from the lineage tracing experiment (refer to the section entitled “Determining prior probabilities of indel formation” for information on how we infer prior probabilities). We then draw *S* values from this interpolated distribution. We then normalize these mutation rates to sum to *p*, therefore allowing in general a *p* probability of mutating a character and 1−*p* probability of remaining uncut. In the case of the “state distribution” simulations (Additional file 1: Fig S13), we say that *p*_*c*_ is distributed as:

$$p_{c} = \theta * Unif(0,1) + (1 - \theta) * F'(x) $$

where *F*^′^(*x*) is the interpolated empirical distribution and *θ* is the mixture component.

Then, we simulate *D* cell divisions, where each cell division consists of allowing a mutation to take place at each character with probability *p*. In the case a mutation takes place, we choose a state to mutate to according to their respective probabilities. Importantly, once a character has been mutated in a cell, that character cannot mutate again.

At the end of the experiment, we sample *M* percent of the cells resulting in 2^*D*^∗*M* cells in the final lineage.

We find that this method for simulating lineages (in particular the method for generating a set of priors on how likely a given state is to form) is able to closely recapitulate observed lineages (Additional file 1: Fig S6).

**Metrics for comparing simulations to empirical data** We used three metrics of complexity to compare simulated clones to real clones:

- *Minimum compatibility distance*: For every pair of character, we define the minimum compatibility distance as the minimum number of cells to be removed to obtain compatibility (Def. 1).
- *Number of observable states per cell*: The number of non-zero or non-missing values for each cell, across all characters (i.e., the amount of data that can be used for a reconstruction, per cell).
- *Number of observable states per character*: The number of non-zero or non-missing values across for each character, across all cells.

#### Parallel evolution simulations for greedy benchmarking

As shown above, our greedy approach should accurately reconstruct a lineage if a perfect phylogeny exists. In order to better quantify how much our greedy algorithm’s performance is affected by parallel mutations, we decided to simulate “near perfect phylogenies,” whereby we first began by simulating a perfect phylogeny, and afterwards introduced double mutated characters.

Specifically, we begin by simulating perfect phylogenies with 40−*k* characters. We then fix a depth, *d*, and sample a node from said depth. We choose two grandchildren randomly from this node (one from each child) and introduce the same mutation on each of the edges from each child to grandchild, thereby violating the perfect phylogeny. We repeat this process *k* times. This thus creates an analysis, as presented in Additional file 1: Fig S5, whereby accuracy can be evaluated as a function of both depth of parallel evolution, *d*, and the number of events that occurred, *k*.

#### Simulation of “base editor” technologies

We used the simulation framework described above to simulate base editor technologies. To explore the trade off between the number of states and the number of characters, we simulated trees with 40,50,80, and 100 characters while maintaining the product of characters and states equal at 400 (thus, we had trees of 10,8,5, and 4 states per character, respectively). The dropout per character was set to 10%, the mutation rate per character was set to 1.04% (a previously observed mutation rate [46]), and 400 cells were sampled from a tree of depth 10. For each character/state regime, we generated 10 trees for assessing the consistency of results. We use a negative binomial model (∼*NB*(5,0.5)) as the editing outcome distribution (i.e., state distribution).

#### Simulation of “phased recorder” technologies

To simulate the phased recorder, we used 5 different experiments varying mutation rates across 50 characters and 10 states per character. In each experiment, we chose a mutation rate for each character from one of 10 regimes, each differing in their relationship to the base mutation rate *p*_0_. To systematically implement this, mutation rate for *χ*_*i*_ is described as such:

$$m_{i} = p_{0} * (1 + e_{j} * \lfloor\frac{i}{5}\rfloor) $$

where *p*_0_=0.025 and *e*_*j*_ is an experiment scalar in **e**={0,0.05,0.1,0.25,0.5}. This means that for characters 1−5, *m*_*i*_=*p*_0_; for characters 6−10, *m*_*i*_=*e*_*j*_*p*_0_; for characters 11−15, *m*_*i*_=2*e*_*j*_*p*_0_; etc. To summarize each experiment, we provide the ratio between the maximum and minimum mutation rates, which is by definition 1+10*r*_*j*_ (because we had 50 characters). We compare two models of indel formation rates—the first being a negative binomial model (∼ *NB*(5,0.5)) and the second being the spline distribution fit from empirical data.

We simulated 10 trees per regime and reconstructed trees with Cassiopeia with and without priors.

### Reconstructions of GESTALT datasets

We downloaded data corresponding to the original GESTALT study [5] and the more recent scGESTALT study from https://datadryad.org/resource/doi:10.5061/dryad.478t9 and GSE105010, respectively. We created character matrices for input into Cassiopeia by creating pivot tables relating each cell the observed indel observed at each one of the 10 tandem sites on the GESTALT recorder. We then reconstructed trees from these character matrices using one of five algorithms: Camin-Sokal (used in the original studies), neighbor joining, Cassiopeia’s greedy method, Cassiopeia’s Steiner tree method, and Cassiopeia’s hybrid method.

For each reconstruction, we record the parsimony of the tree, corresponding to the number of mutations that are inferred along the reconstructed tree. We display these findings in Fig. 6a, where we have *Z*-normalized the parsimonies across the methods for each dataset to enable easier visualization of relative performances.

### Visualization of trees

To visualize trees, we use the iTOL interface [64]. Colors in the heatmap denote a unique mutation, gray denotes an uncut site, and white denotes dropout.

## Supplementary information

**Additional file 1** Fig S1. Time complexity of lineage reconstruction approaches. Fig S2. Evaluation of the stability of the maximum neighborhood size parameter. Fig S3. Observed Frequency of Mutation is a Measure of True Mutation Count. Fig S4. Precision of Cassiopeia-Greedy First Split. Fig S5. Benchmarking of parallel evolution on the greedy heuristic. Fig S6. Determination of mutation rates used in simulation. Fig S7. Triplets Correct Statistic. Fig S8. Unthresholded Triplets Correct. Fig S9. Parsimony of reconstructed trees of 400 cell simulated datasets. Fig S10. Benchmarking of lineage tracing algorithms on 1000 cell synthetic datasets. Fig S11. Benchmarking of greedy and hybrid algorithms on large experiments. Fig S12. Bootstrapping analysis of Cassiopeia and Neighbor-Joining with the Transfer Bootstrap Expectation statistic. Fig S13. Reconstruction accuracy under over-dispersed state distributions. Fig S14. Observed Proportion of Parallel Evolution in Simulations. Fig S15. Determination of the indel prior transformation function. Fig S16. Incorporation of priors into Cassiopeia. Figure S17. Quality control metrics for the target-site sequencing library processing pipeline. Fig S18. Processing pipeline for the in vitro dataset. Fig S19. Identification of doublets using intBCs. Fig S20. Estimation of Prior Probabilities for Tree Reconstruction. Fig S21. Evaluation of algorithms on in vitro lineage tracing clones, First Split. Fig S22. Evaluation of algorithms on in vitro lineage tracing clones, Second Split. Fig S23. Exhaustion of Target Sites across Clones. Fig S24. Vignette of Inferential Mistakes for Clone 3. Fig S25. Parsimony scores from reconstructions of the GESTALT datasets. Fig S26. “Phased Recorder” leverages variability across target sites.

**Additional file 2** Review history.
