# Supplementary material for: Inference of single-cell phylogenies from lineage tracing data using Cassiopeia
Source: Genome Biol. 2020 Apr 14;21:92. doi: 10.1186/s13059-020-02000-8 (PMC7155257; doi:10.1186/s13059-020-02000-8)
Supplement: Supplementary file 1 — Additional file 1 Fig S1. Time complexity of lineage reconstruction approaches. Fig S2. Evaluation of the stability of the maximum neighborhood size parameter. Fig S3. Observed Frequency of Mutation is a Measure of True Mutation Count. Fig S4. Precision of Cassiopeia-Greedy First Split. Fig S5. Benchmarking of parallel evolution on the greedy heuristic. Fig S6. Determination of mutation rates used in simulation. Fig S7. Triplets Correct Statistic. Fig S8. Unthresholded Triplets Correct. Fig S9. Parsimony of reconstructed trees of 400 cell simulated datasets. Fig S10. Benchmarking of lineage tracing algorithms on 1000 cell synthetic datasets. Fig S11. Benchmarking of greedy and hybrid algorithms on large experiments. Fig S12. Bootstrapping analysis of Cassiopeia and Neighbor-Joining with the Transfer Bootstrap Expectation statistic. Fig S13. Reconstruction accuracy under over-dispersed state distributions. Fig S14. Observed Proportion of Parallel Evolution in Simulations. Fig S15. Determination of the indel prior transformation function. Fig S16. Incorporation of priors into Cassiopeia. Figure S17. Quality control metrics for the target-site sequencing library processing pipeline. Fig S18. Processing pipeline for the in vitro dataset. Fig S19. Identification of doublets using intBCs. Fig S20. Estimation of Prior Probabilities for Tree Reconstruction. Fig S21. Evaluation of algorithms on in vitro lineage tracing clones, First Split. Fig S22. Evaluation of algorithms on in vitro lineage tracing clones, Second Split. Fig S23. Exhaustion of Target Sites across Clones. Fig S24. Vignette of Inferential Mistakes for Clone 3. Fig S25. Parsimony scores from reconstructions of the GESTALT datasets. Fig S26. “Phased Recorder” leverages variability across target sites. [file 13059_2020_2000_MOESM1_ESM.pdf]

Supplementary Figures for "Inference of single-cell phylogenies from lineage tracing data using Cassiopeia"

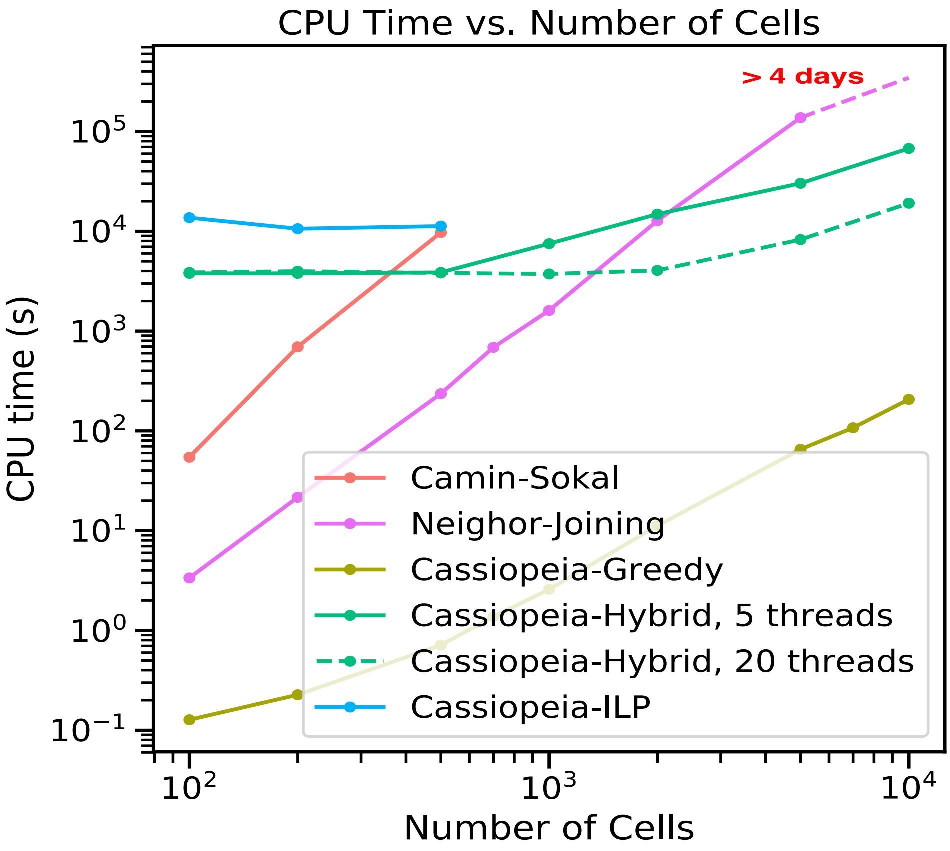

Fig. S1. Time complexity of lineage reconstruction approaches. Time complexity, as measured in seconds, of each algorithm tested in this manuscript is compared using simulated datasets ranging from 100 cells to 10,000 cells. Default settings for the simulations were used (0.025 mutation rate, 40 characters, 10 states, and 0.18 median dropout rate). Cassiopeia was tested using default parameters of a maximum neighborhood size of 3000, time to converge of one hour, and a greedy cutoff of 200 cells. Cassiopeia was tested using 5 threads and 20 threads, illustrating the advantage of parallelizing the reconstruction algorithm. ILP, which was only run until 500 cells due to the infeasibility of running on larger datasets, was allowed 10000s to converge on a maximum neighborhood size of 20,000 (the default settings). Neighbor-Joining could not reconstruct a tree for 10,000 cells within 4 days when the reconstruction was terminated.

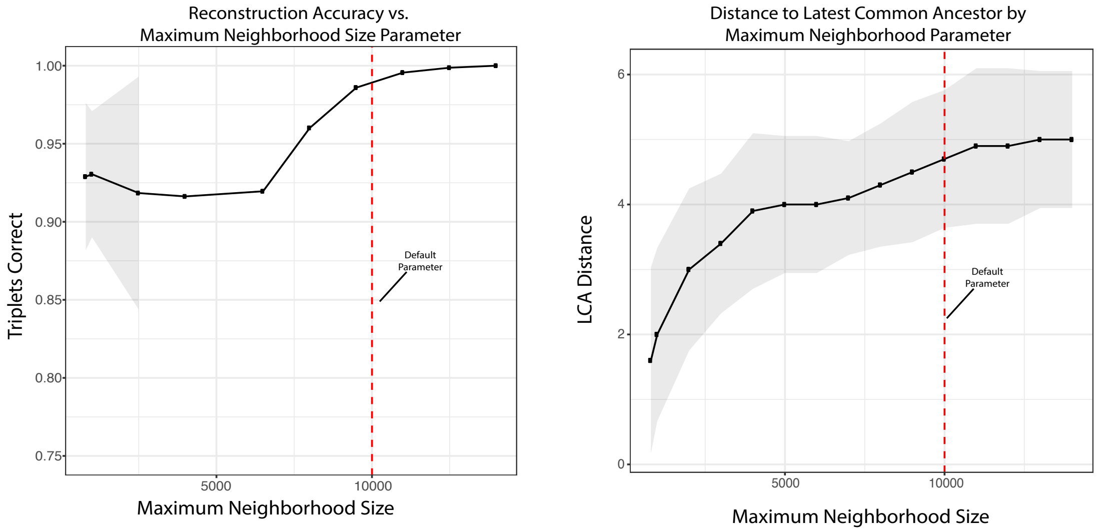

Fig. S2. Evaluation of the stability of the maximum neighborhood size parameter. The maximum neighborhood size is a central parameter provided by the user when inferring the potential graph necessary as input to the Steiner-Tree solver (see methods). Here, we benchmark the stability of solutions with respect to several maximum neighborhood sizes using 10 trees with default parameters (40 characters, 40 states, 2.5% per-character mutation rate, depth of 11, and an average dropout rate of 17% per character). We quantify both the reconstruction accuracy with respect to the reconstructions found with the largest maximum neighborhood size ($14,000$ nodes) which displays a saturation at around $9,000$ nodes. To provide intuition for the accuracy of the potential graph (represented as the maximum distance to the 'latest common ancestor' (LCA) which is dynamically solved for, given a maximum neighborhood size) we display the LCA allowed for each maximum neighborhood size parameter. In both figures, we display lines connecting the mean values; shaded regions are the standard deviation of the measurements across the 10 replicates.

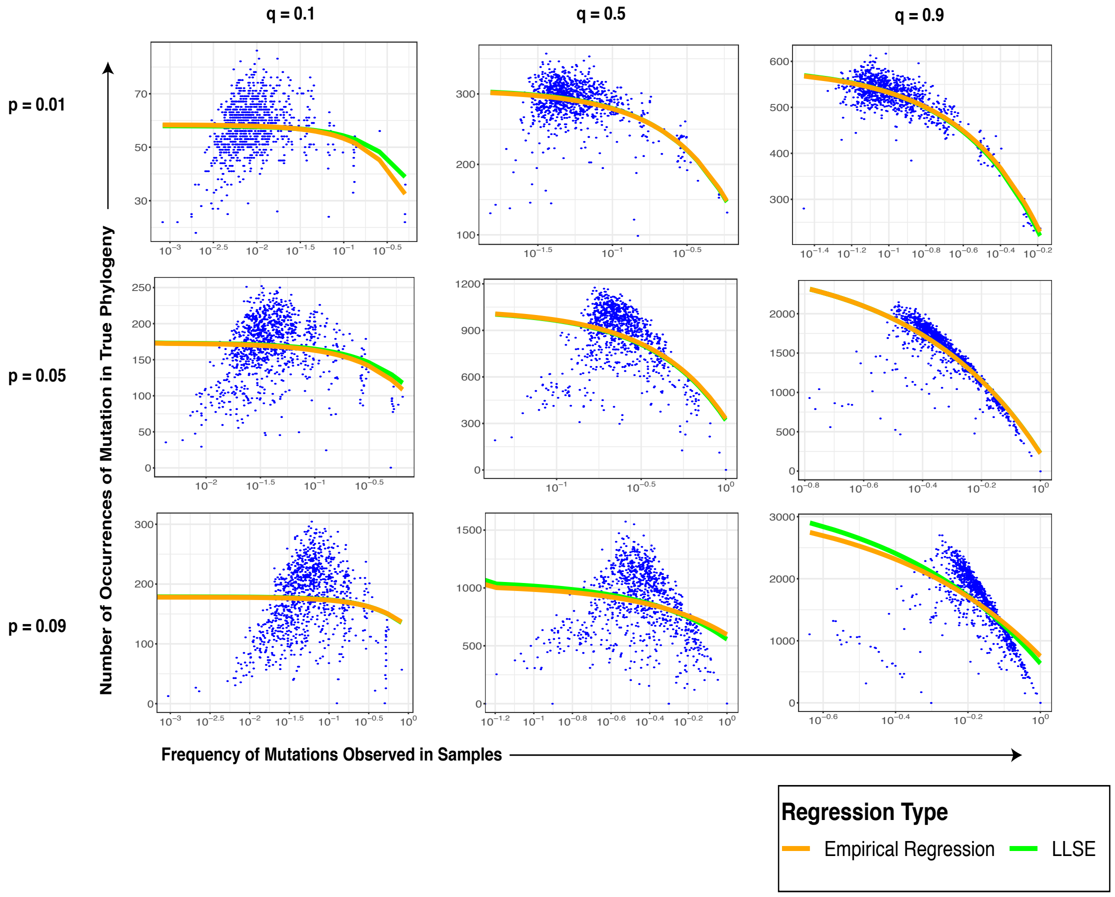

Fig. S3. Observed Frequency of Mutations is Measure of True Mutation Count. The true number of occurrences of a mutation is estimated well by the observed frequency at leaves. We use a Linear Least Squares Estimate to quantify the relationship between the expected number of times a mutation occurred given the observed frequency at the leaves (Eq. 1). Using various rates for character and indel mutation rates (p and q, respectively) we show that this relationship is negative (i.e. greater observed frequencies tend to correspond to mutations that occurred few times near the top of the phylogeny) for a range of biologically-relevant values.

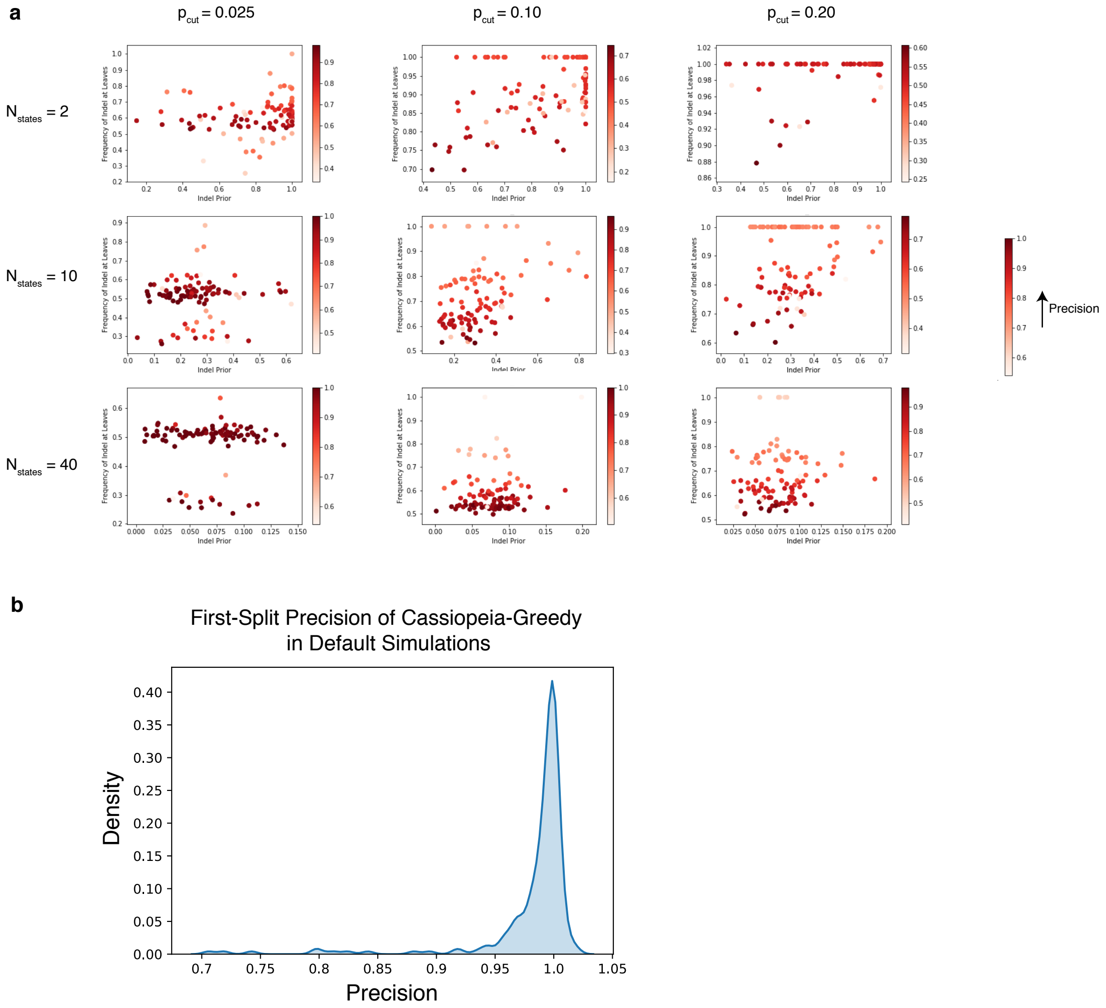

Fig. S4. Precision of Cassiopeia-Greedy First Split. (a) The precision of greedy splits of 400 cells was measured with varying mutation rates and states per character, wihtout dropout. For each pair of parameters (number of states and mutation rate), we measure precision as a function of the conditional probability of the selected (character, state) pair and the frequency of that mutation observed in the 400 cells. (The conditional probability for state j, $q(j)$ is defined as $Pr(\chi \rightarrow j|\chi \;\; mutates)$). Precision was defined as the proportion of true positives in the greedy split (see Methods). Each point indicates a replicate (100 per plot) and the heat represents the precision. (b) The density histogram (smoothed using a kernel density estimation procedure) of all first-split precision statistics from Cassiopeia-Greedy on default simulations (i.e. 40 characters, 40 states, 2.5% mutation rate, 11 generations, 400 cells, and 18% dropout rate). We measured a median precision of 0.99 across all default simulations.

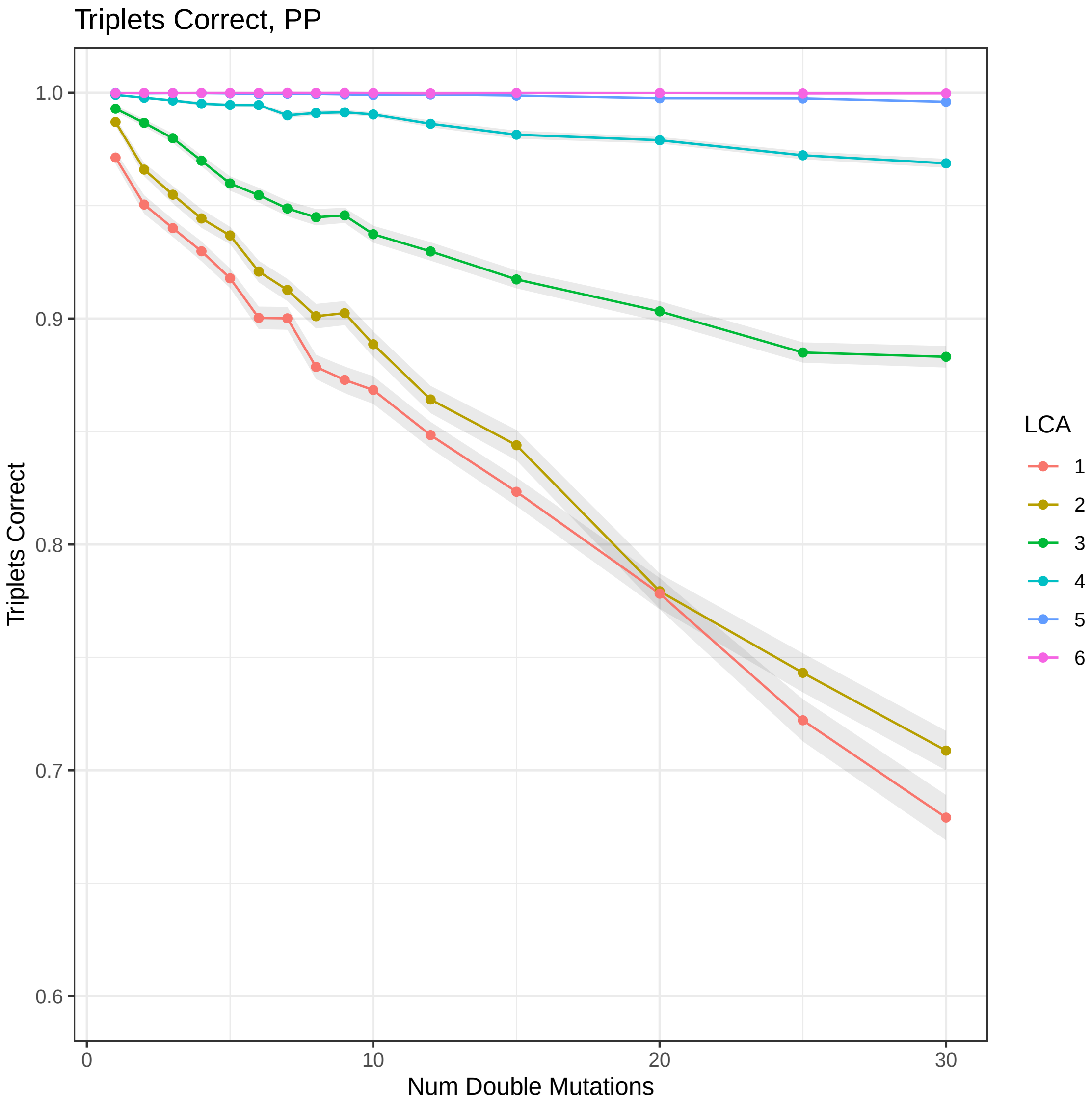

Fig. S5. Benchmarking of parallel evolution on the greedy heuristic. The greedy heuristic, inspired by algorithms to solve the case of perfect phylogeny (see methods), is impacted by two factors: (1) the number of parallel evolution events (i.e. the same mutation occurs more than once in the experiment) and (2) the depth from the root these mutations occur at. Here, each line represents a series of experiments increasing the number of 'double mutations' (i.e. the simplest case of parallel evolution where a mutation occurs exactly twice) where the 'latest common ancestor' (LCA) is a set depth from the root.

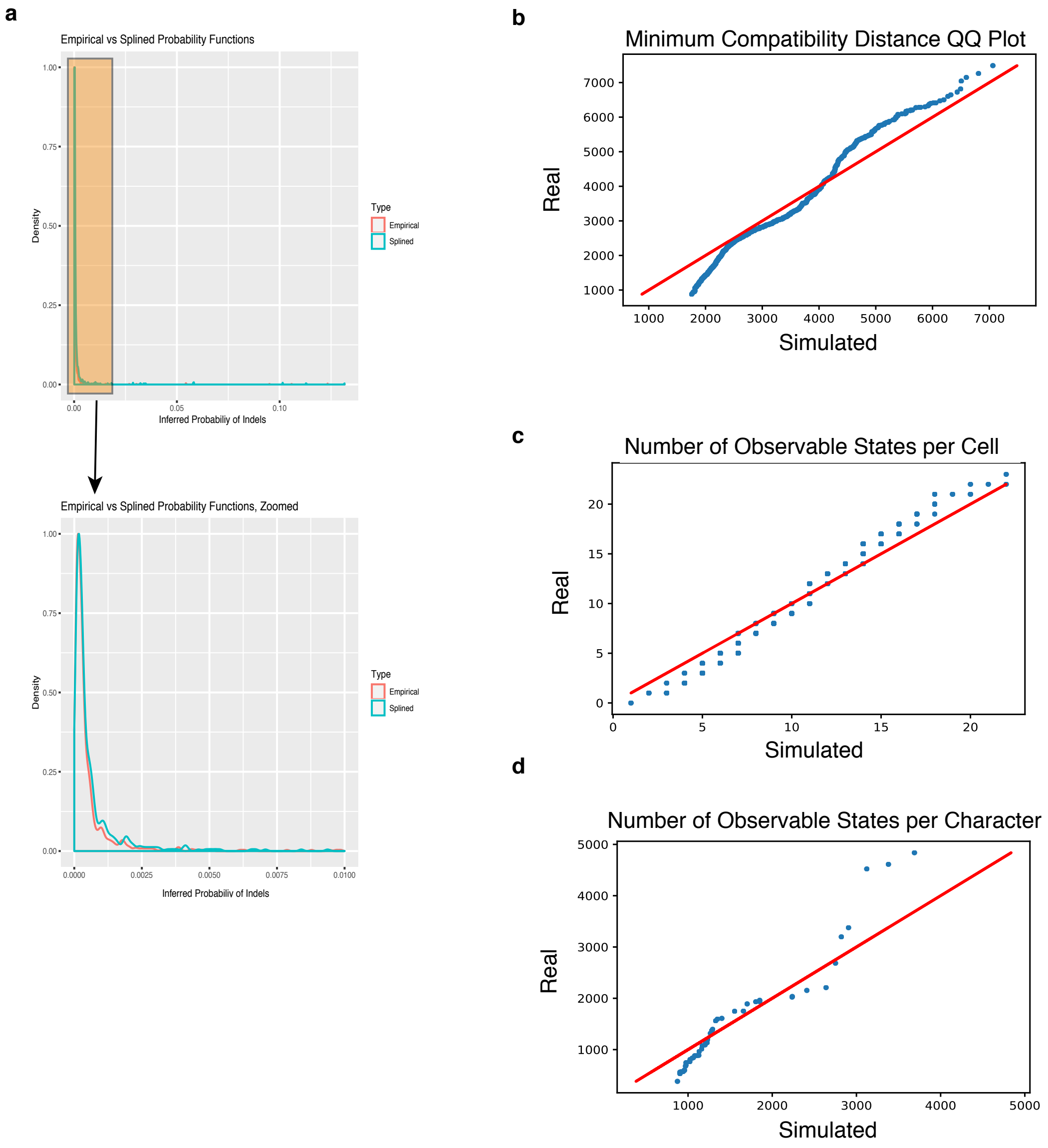

Fig. S6. Determination of mutation rates used in simulation. We use an interpolation of the empirical indel distribution as input for the conditional probability of a state arising given a mutation. (a) A comparison of the empirical and 'splined' indel distributions; a zoomed in version is provided for comparison at low probabilities. (b-c) A comparison of three metrics between an observed clone (clone 3) and a simulated clone using inferred parameters. We used the number of character, states, per-character mutation rate, and dropout probabilities inferred from the empirical data; the indel formation rates were calculated using a polynomial spline function. (b) measures the 'minimum compatibility distance' for all pair-wise character combinations (see methods). (c) compares the number of observable states per cell. (d) compares the number of observable states per character.

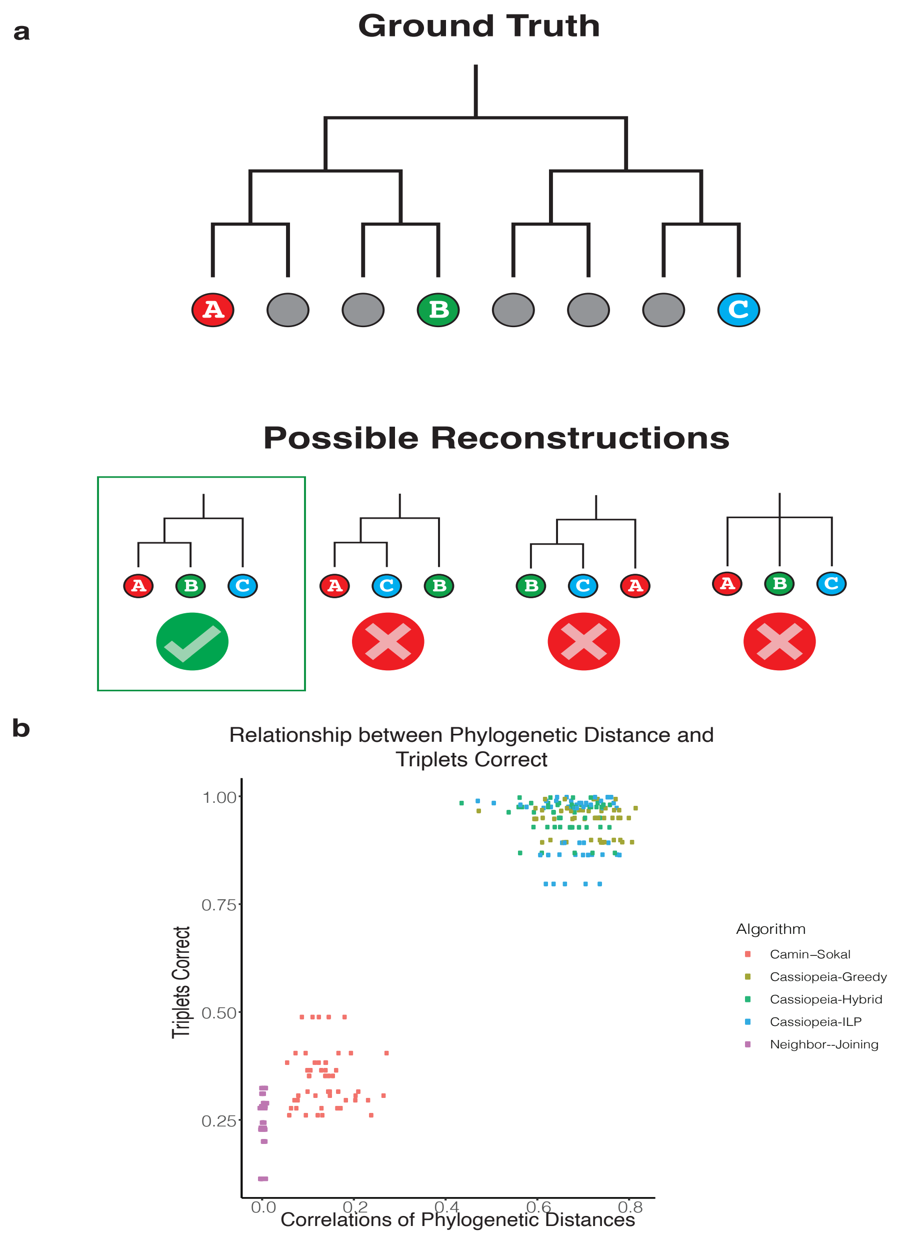

Fig. S7. Triplets Correct Statistic. (a) Schematic for the Triplets Correct statistic, the combinatorial metric used to compare between trees. In this metric, we compare the relative orderings of three leaves between two trees (e.g. the "Ground Truth" and a reconstruction). There are four possible ways that a triplet could be ordered here, based on the relationship between each leaf and the Latest Common Ancestor (LCA) of the triplet. The statistic tallies the number of correct triplets and reports this value weighted by the depth of the LCA from the root. Importantly, this statistic is designed to avoid concerns of inappropriately weighting early splits as these might dominate the statistic. Specifically, the triplets are stratified in accordance to the depth of the LCA and the triplets correct is reported as an average across all LCA depths. This way, LCAs near the root will not dominate the score. (b) A comparison between the triplets correct statistic and the phylogenetic distance correlation (defined as the correlation of node-node distances between a simulated and reconstructed tree; see Methods) where we observe a Pearson correlation of 0.96.

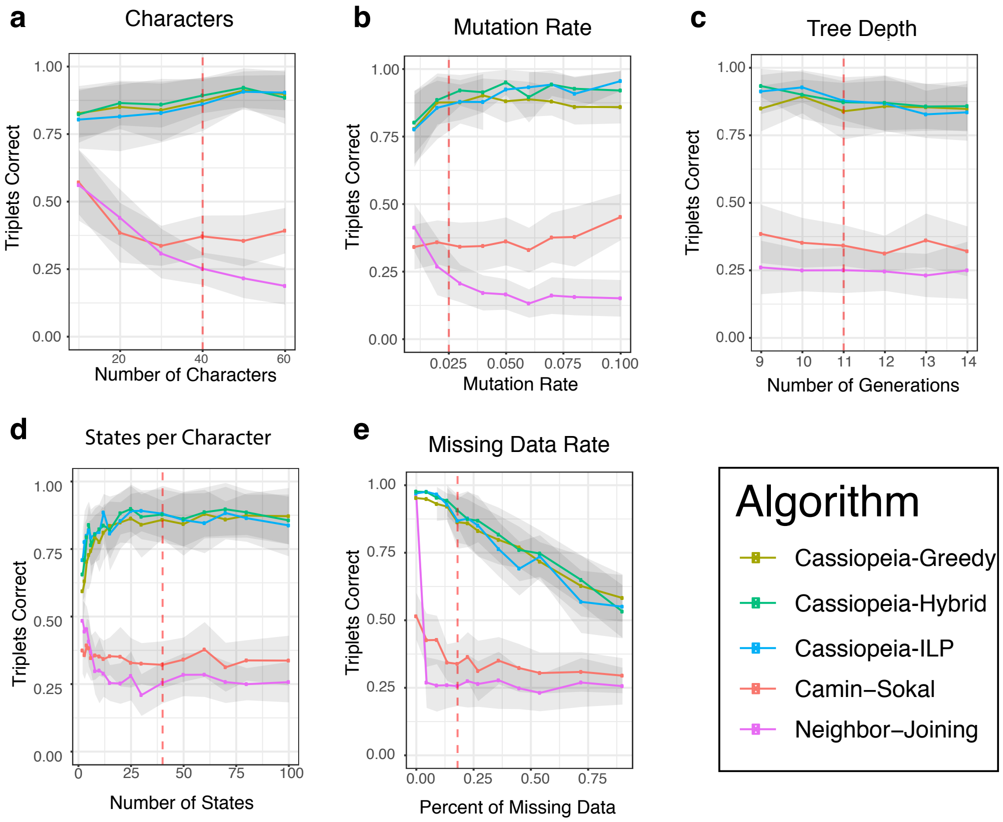

Fig. S8. Unthresholded Triplets Correct. The Triplets Correct statistic reported for synthetic benchmarks presented in Figure 2 without removing triplets whose LCA-depth was sampled deeply enough (by default, a given triplet at depth D is only considered if a sufficient number of triplets at depth D is observed). Here, the effective threshold is 0.

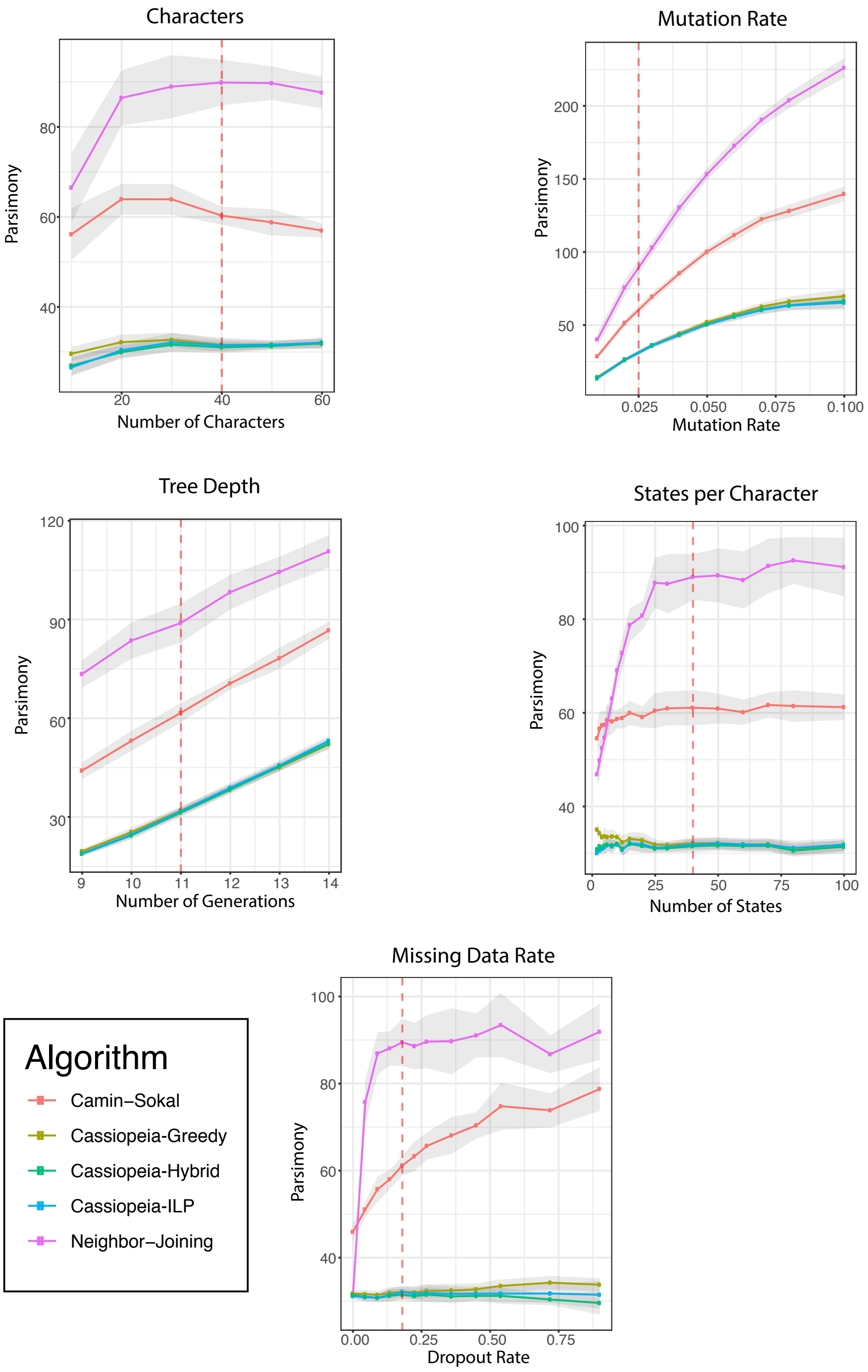

Fig. S9. Parsimony of reconstructed trees of 400 cell simulated datasets. Parsimony scores (or number of evolutionary events) for each reconstructed network presented in Figure 2 were calculated and compared across phylogeny reconstruction methods. Results are presented for the number of characters, the mutation rate, tree depth, number of states and dropout rate for all five algorithms used in this study. Standard error is represented by shaded area.

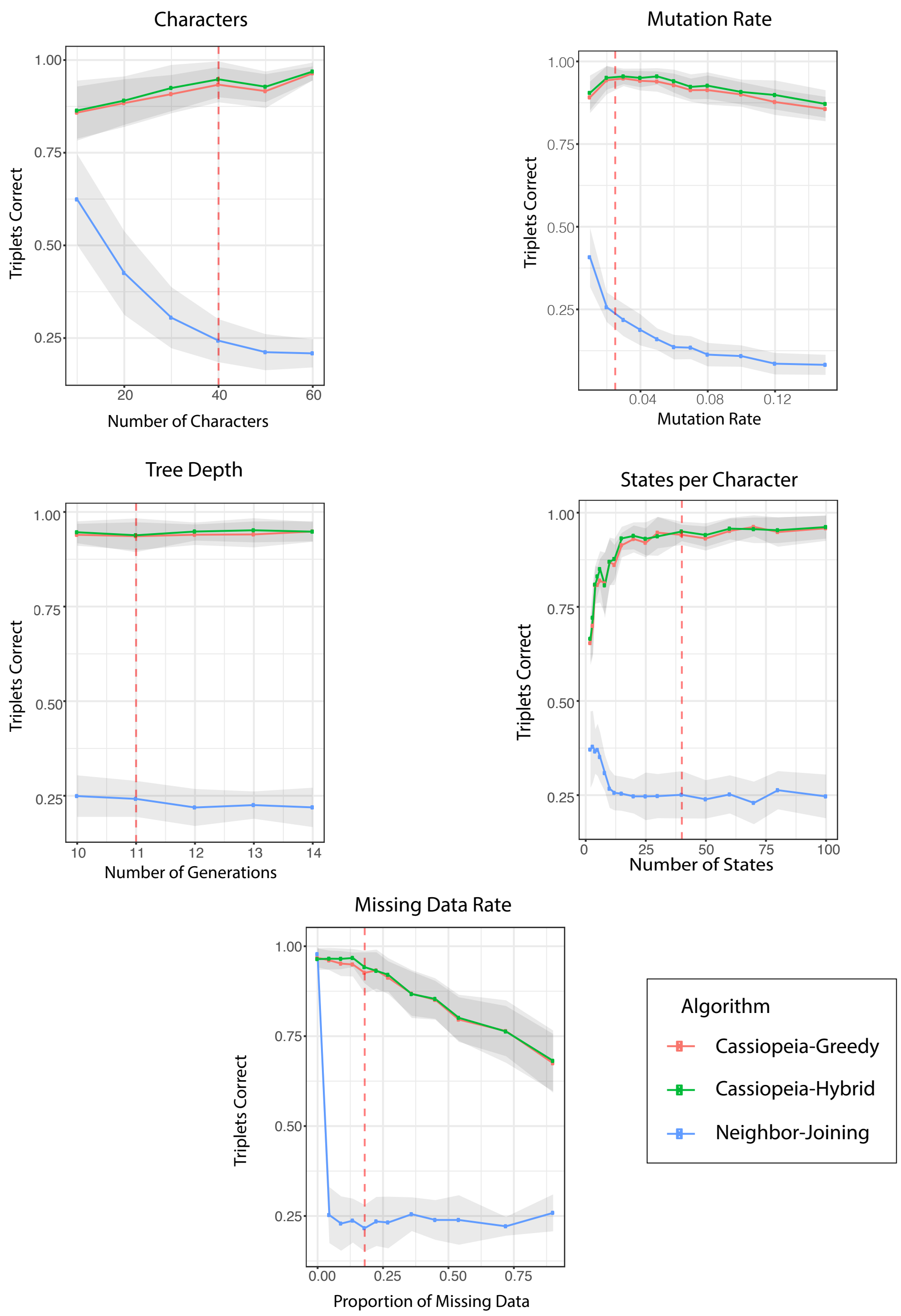

Fig. S10. Benchmarking of lineage tracing algorithms on 1000 cell synthetic datasets. Phylogeny reconstruction algorithms were benchmarked on simulated trees consisting of 1,000 cells. The number of characters, character-wise mutation rate, length of experiment or tree depth, number of states, and dropout rate were tested. Due to scalability issues, only greedy, hybrid, and neighbor-joining were tested. Standard error is represented by shaded area.

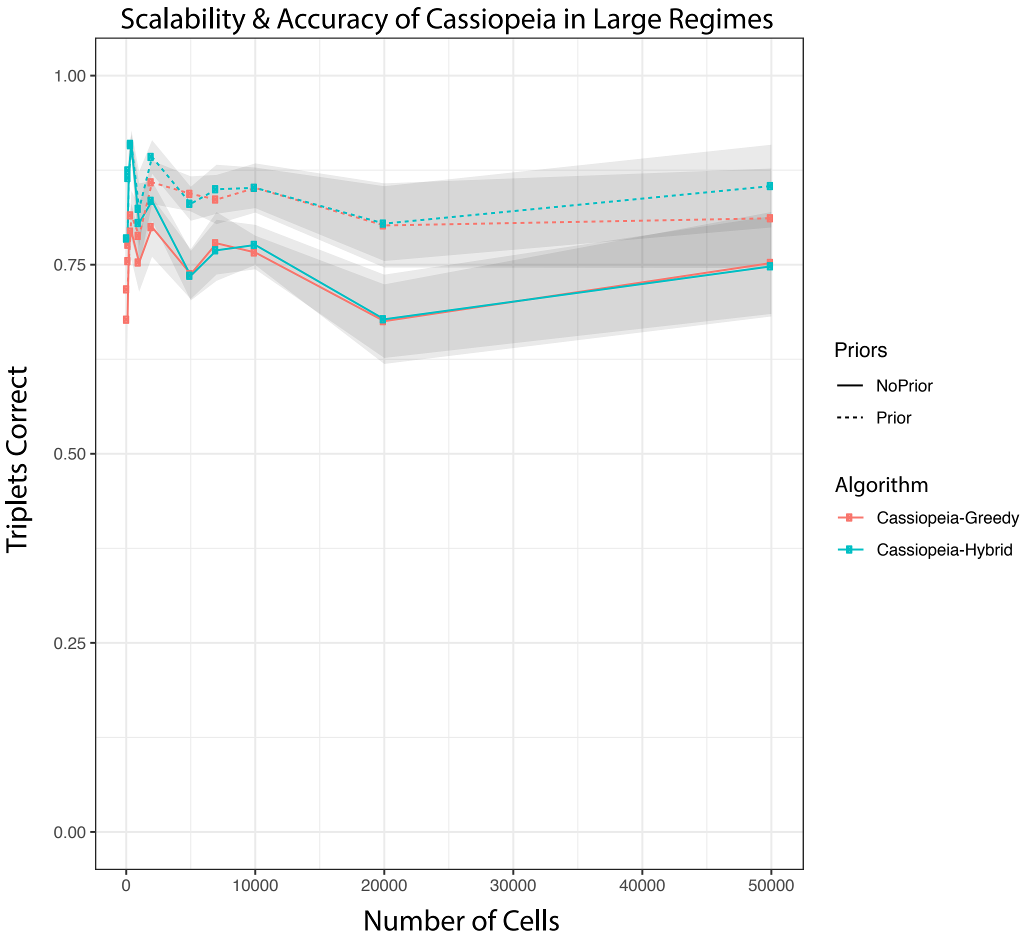

Fig. S11. Benchmarking of greedy and hybrid algorithms on large experiments. Triplets correct is used to measure the accuracy of reconstructions using both hybrid and greedy algorithms on large trees (up to $50,000$ cells). Of note, hybrid and greedy have comparable results on larger trees, which remain accurate even in these massive regimes. In addition, the knowledge of prior probabilities of particular states confers a large increase in accuracy.

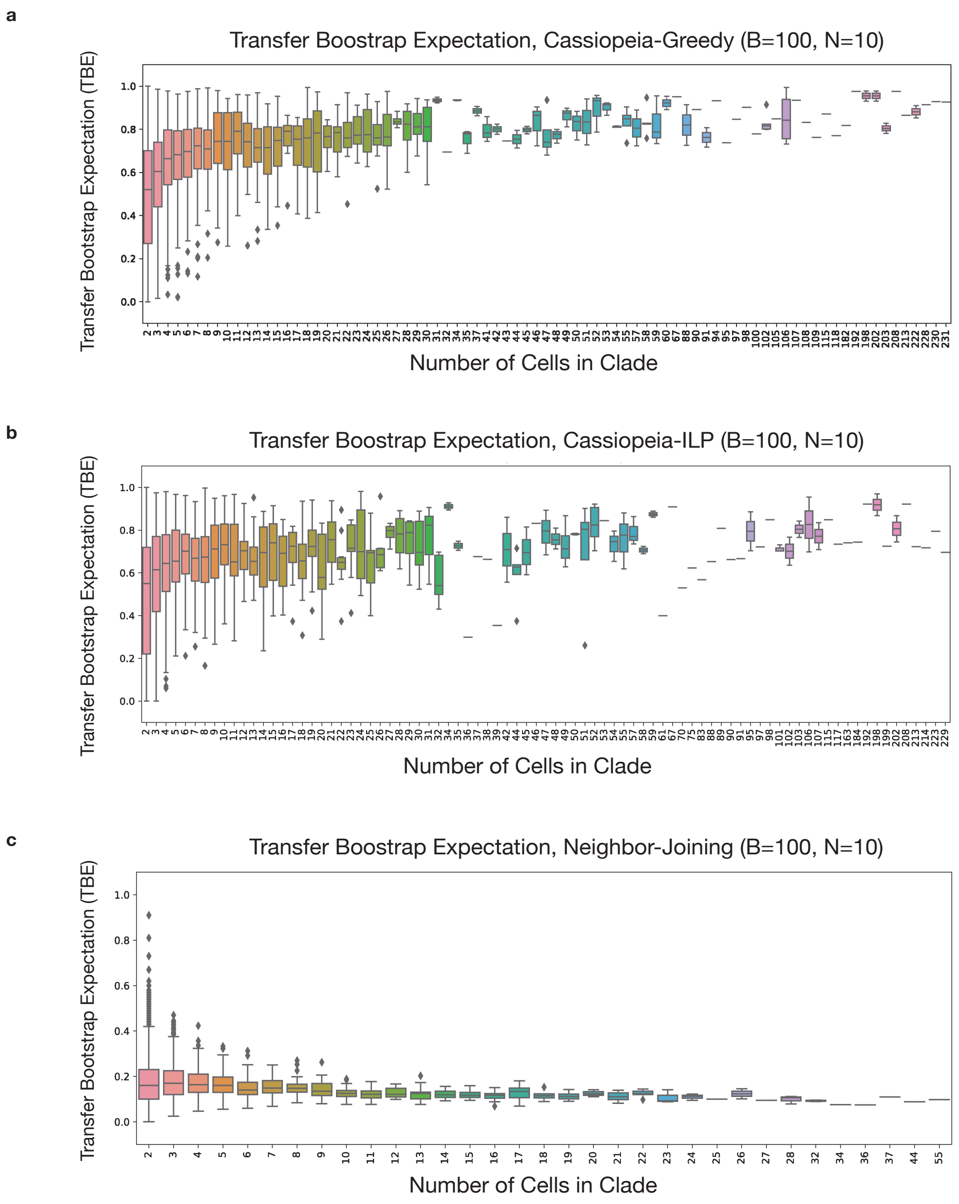

Fig. S12. Bootstrapping analysis of Cassiopeia and Neighbor-Joining with the Transfer Bootstrap Expectation statistic. Bootstrap analysis of robustness for Cassiopeia-Greedy (a) , -ILP (b) , and Neighbor-Joining (c). 100 bootstrap samples ($B = 100$) were taken for 10 simulated trees ($N = 10$) by sampling characters with replacement and each matrix was used for reconstruction by each of the tree algorithms. The Booster software [61] was used to assess robustness of each clade in the original reconstruction, as measured with the Transfer Bootstrap Expectation (TBE) statistic. The distribution of TBE's is shown for each algorithm as a function of the size of the clade (i.e. a clade with two leaves underneath it will be of size 2

.

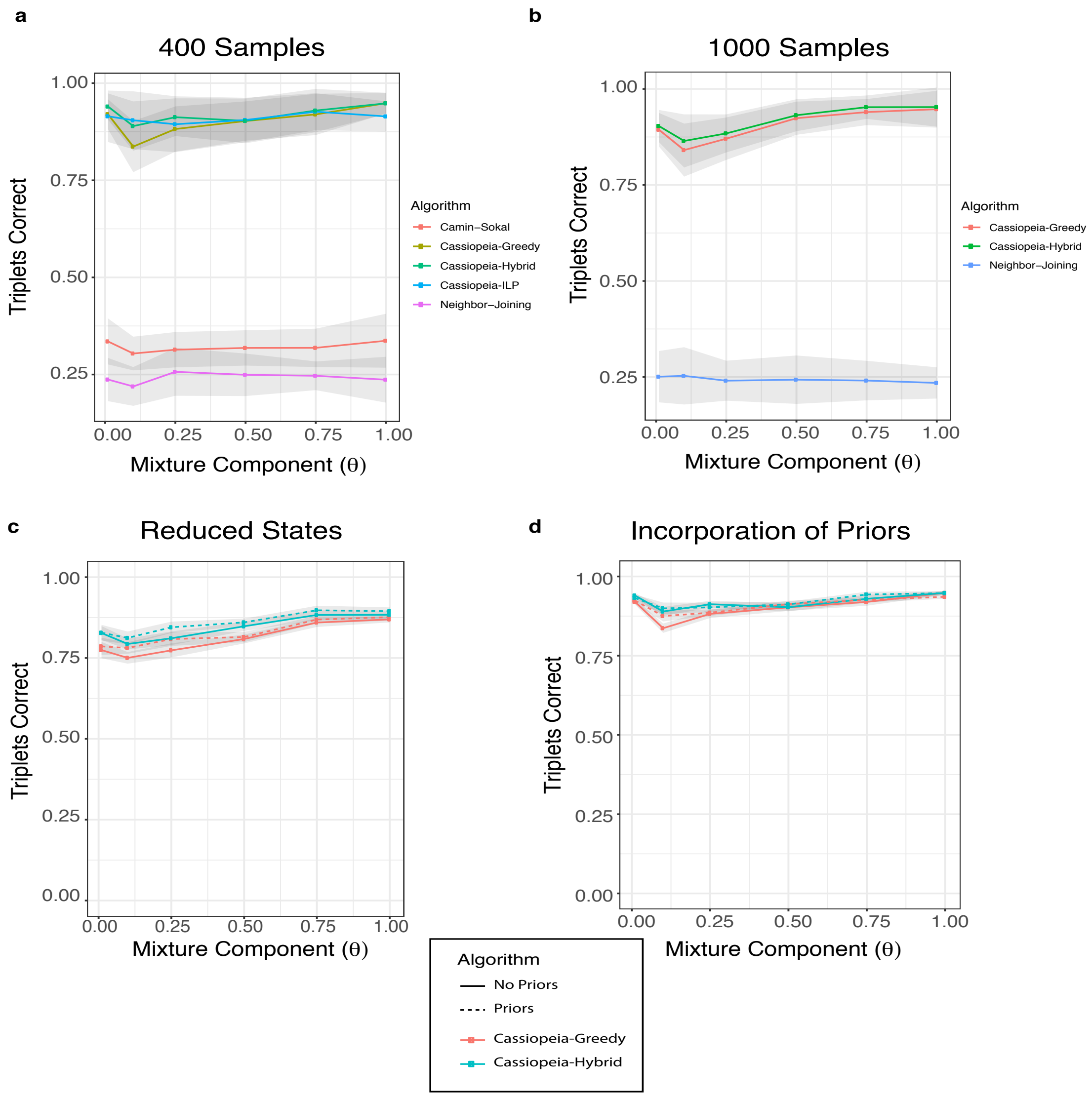

Fig. S13. Reconstruction accuracy under over-dispersed state distributions. The effect of the indel distribution (i.e. the relative propensity for a given indel outcome) was explored in various regimes using a mixture model. Here, the mixture model consisted of mixing the inferred indel distribution with a uniform distribution between 0 and 1.0 with some probability θ (i.e. when $\theta = 1.0$, the indel distribution was uniform). In all simulations, we used default parameters for the simulated trees unless stated otherwise (40 characters, 40 states, depth of 11, median dropout rate of 17%, and a character mutation rate of 2.5%). (a) displays the results of all five algorithms over 400 samples. (b) displays results for simulations over 1000 samples for hybrid, greedy, and neighbor-joining methods. (c) Simulations for 400 samples using 10 states rather than 40 states per character. Dashed lines represent reconstructions performed with priors. (d) Simulations over 400 samples and 40 states, comparing results with and without priors. Dashed lines represent reconstructions performed with priors.

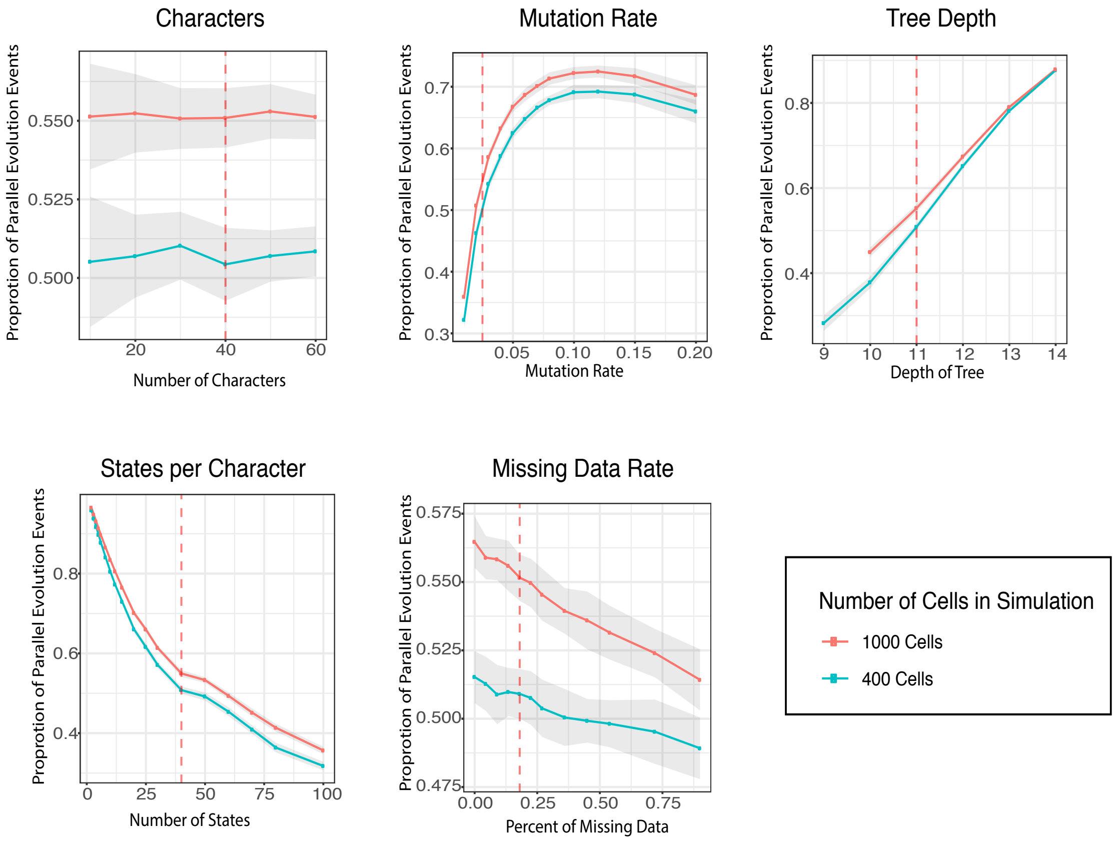

Fig. S14. Observed Proportion of Parallel Evolution in Simulations. Inferred proportion of parallel evolution, as defined by teh proportion of mutations that are observed more than once in a given tree, for the simulations presented in Figure 2 and Fig S10.

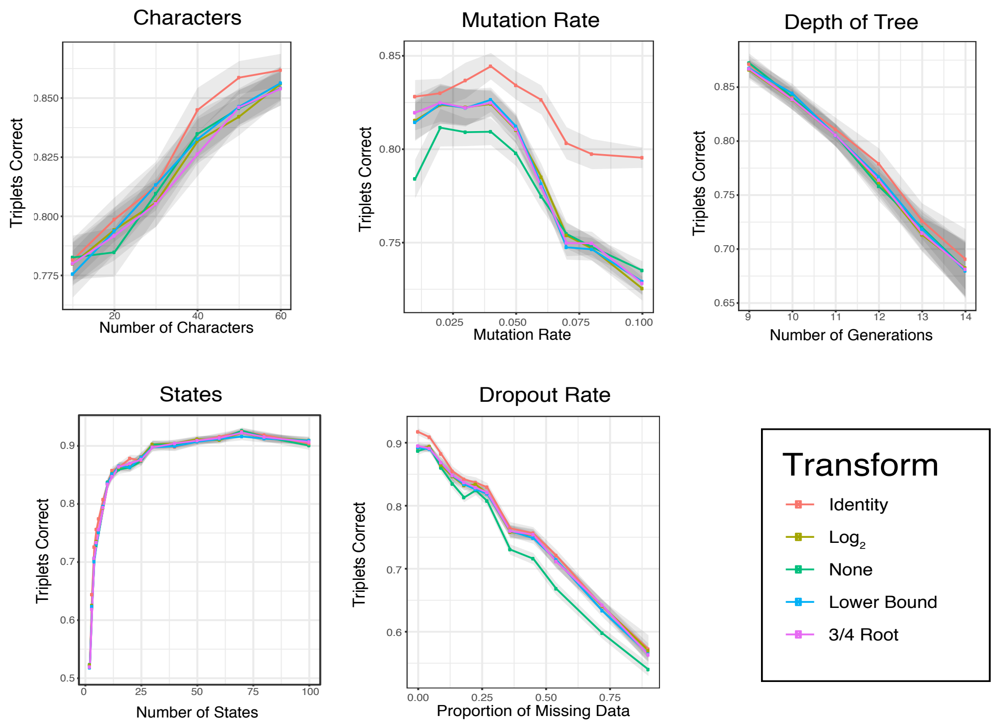

Fig. S15. Determination of the indel prior transformation function. The effect of incorporating the prior probabilities of mutation events into the greedy algorithm is explored using synthetic datasets. The exact mutation probabilities used for simulations are used during reconstruction (i.e. the mutations drawn during simulation). Five possible transformations $f(n_{i,j})$, representing an approximation of the future penalty of not choosing this mutation (see methods) were tested for incorporation with the priors. The transformations were: (i) Identity ($f(n_{i,j}) = n_{i,j}$), (ii) Log2 ($f(n_{i,j}) = log_2(n_{i,j})$), (iii) None ($f(n_{i,j}) = 1$), (iv) Lower Bound ($f(n_{i,j}) = min(n_{i,j}, \frac{N}{20.0})$), and (v) $\frac{3}{4}$ root ($f(n_{i,j}) = (n_{i,j})^{\frac{3}{4}}$). $n_{i,j}$ denotes the number of cells which report the mutation j in character i and N is the total number of samples. To test these transformations, we evaluated the resulting tree accuracy via Triplets Correct. Standard error is represented by shaded area.

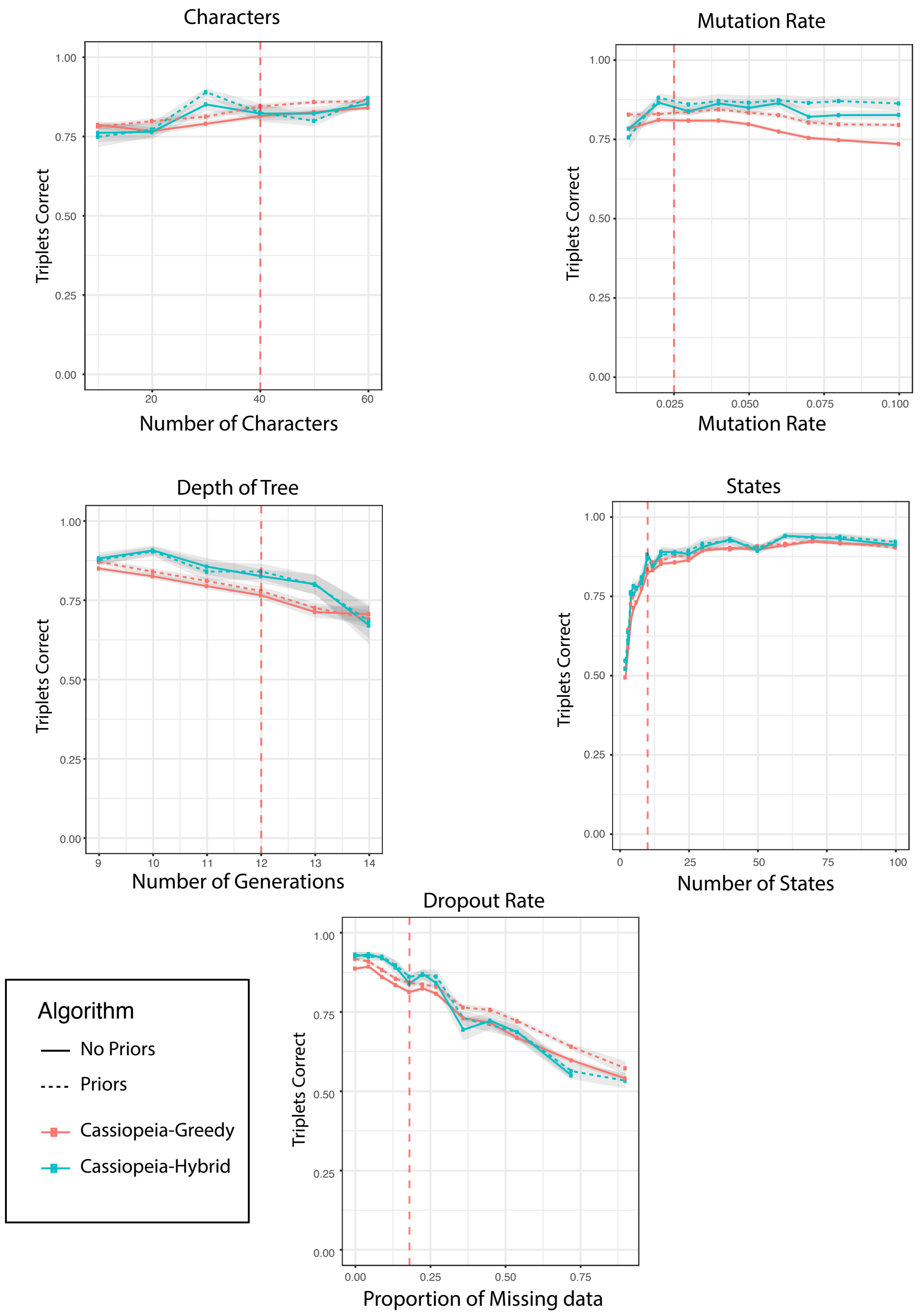

Fig. S16. Incorporation of priors into Cassiopeia. A comparison of tree accuracy when using priors for both the greedy-only method and Cassiopeia. We compared performance as we varied the number of characters per cell, the mutation rate per character, the length of the experiment, the number of states per character, and the amount of missing data. Standard error is represented by shaded area.

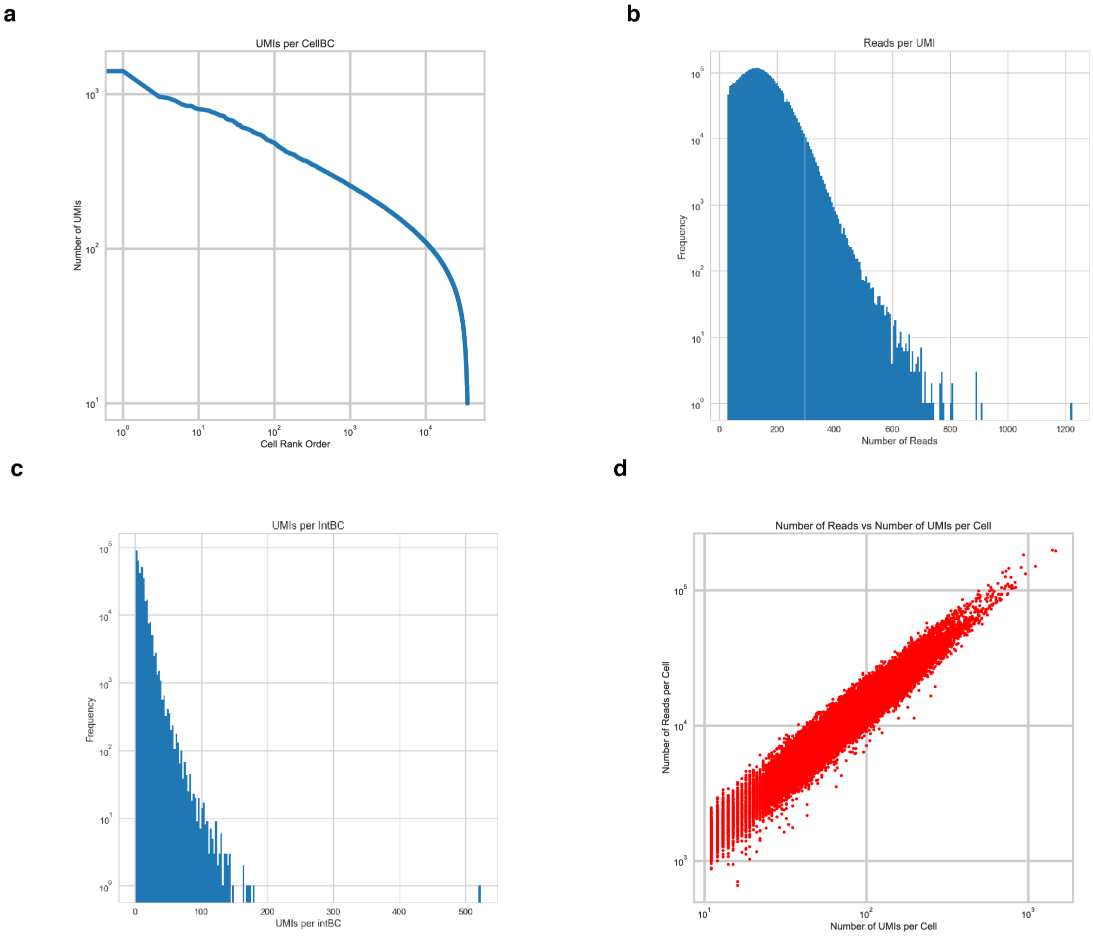

Fig. S17. Quality control metrics for the target site sequencing library processing pipeline. (a-d) present quality control metrics after the processing pipeline. (a) Cells are ranked by the number of UMIs they contain, showing a median of 76; (b) The number of reads per UMI after UMI error correction and collaping, showing a median of 137; (c) The number of UMIs per integration barcode (intBC), showing a median of 7; (d) is the concordance between reads per cellBC and UMIs per cellBC, showing a pearson correlation of 0.96

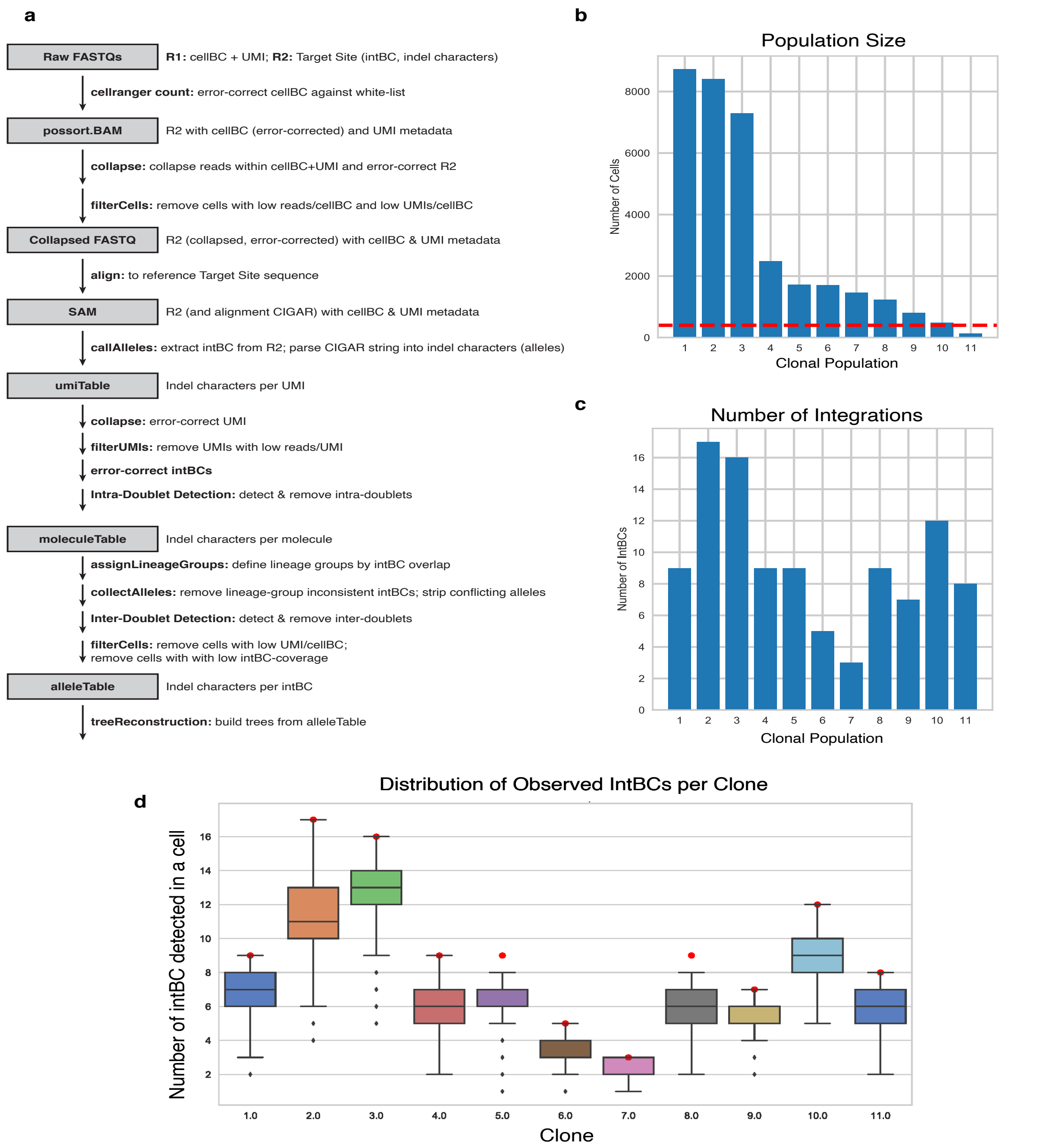

Fig. S18. Processing Pipeline for the *in vitro* dataset. (a) shows a more in-depth flowchart of the Cassiopeia processing pipeline taking as input the raw FASTQs from a sequencing run and converting the observed reads into final trees. Cellranger “count” is used to map reads to dummy transcriptome (junk sequence that nothing will align to), filter cells, and read off the 10x cell barcodes and UMIs. The resulting BAM file is then passed through a series of cell filtering, UMI error correction, and allele mapping before becoming the final allele table that can be converted to character matrices for clone reconstruction. See methods for more detailed information for each step. (b-d) present additional summary statistics for the final allele table. (b) displays the number of cells per clone; (c) shows the median number of intBCs observed in each clone; (d) shows the distribution of the number of intBCs observed in each cell (red points are references to indicate the number of intBCs used to reconstruct the particular clone).

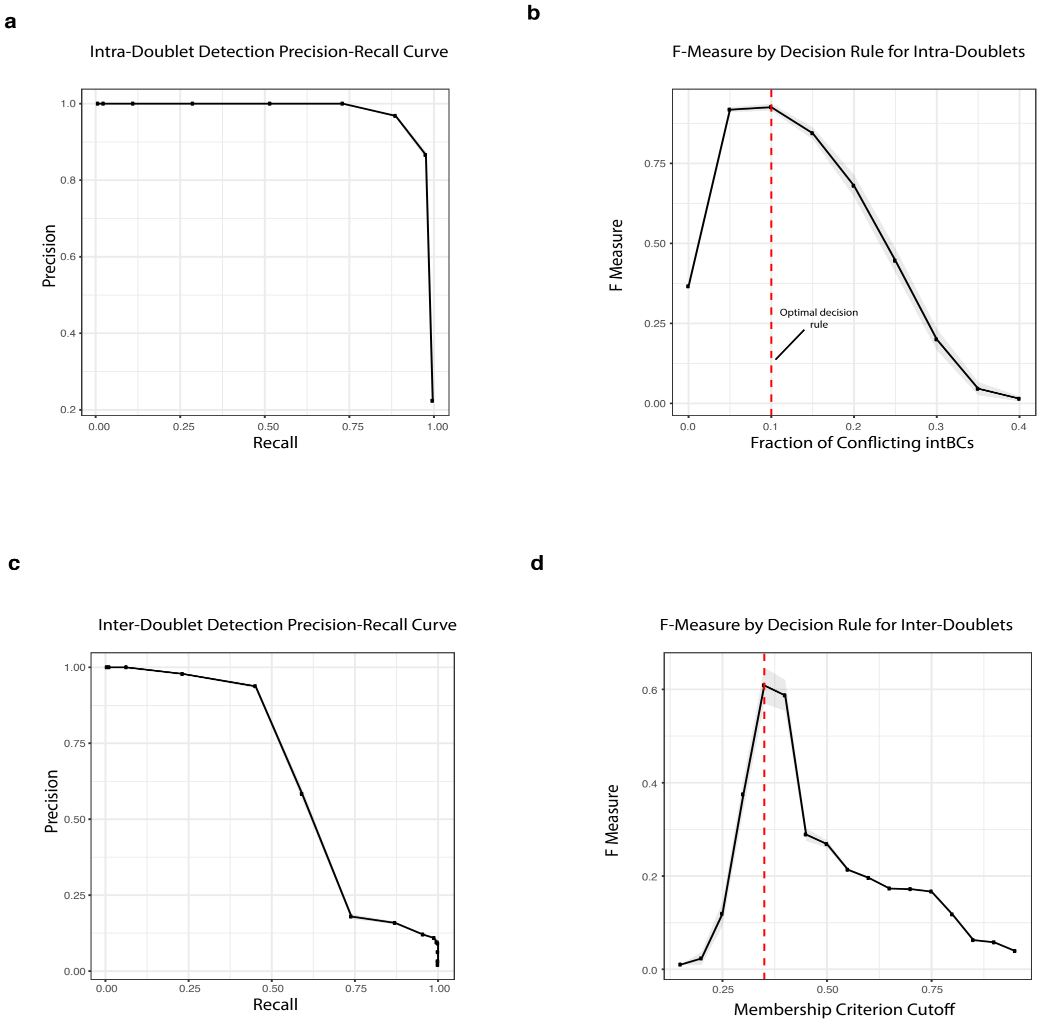

Fig. S19. Identification of doublets using intBCs. IntBCs are used to identify doublets. (a-b) report the ability to identify doublets arising from the same clone, referred to as "intra"-doublets; (c-d) report the ability to identify doublets arising from different clones, referred to as "inter"-doublets. Doublets were simulated using the final allele table and 200 "intra"- and "inter"-doublets were created in each of 20 replicates. Precision-recall curves for intra- and inter-doublet detection methods are presented in (a) and (b), respectively. (c) and (d) present the F-measure (defined as the weighted harmonic mean between precision and recall) of detection methods for intra- and inter-doublets, respectively. Red-dashed lines denote the optimal decision rule for doublet detection. Standard error is represented by shaded area.

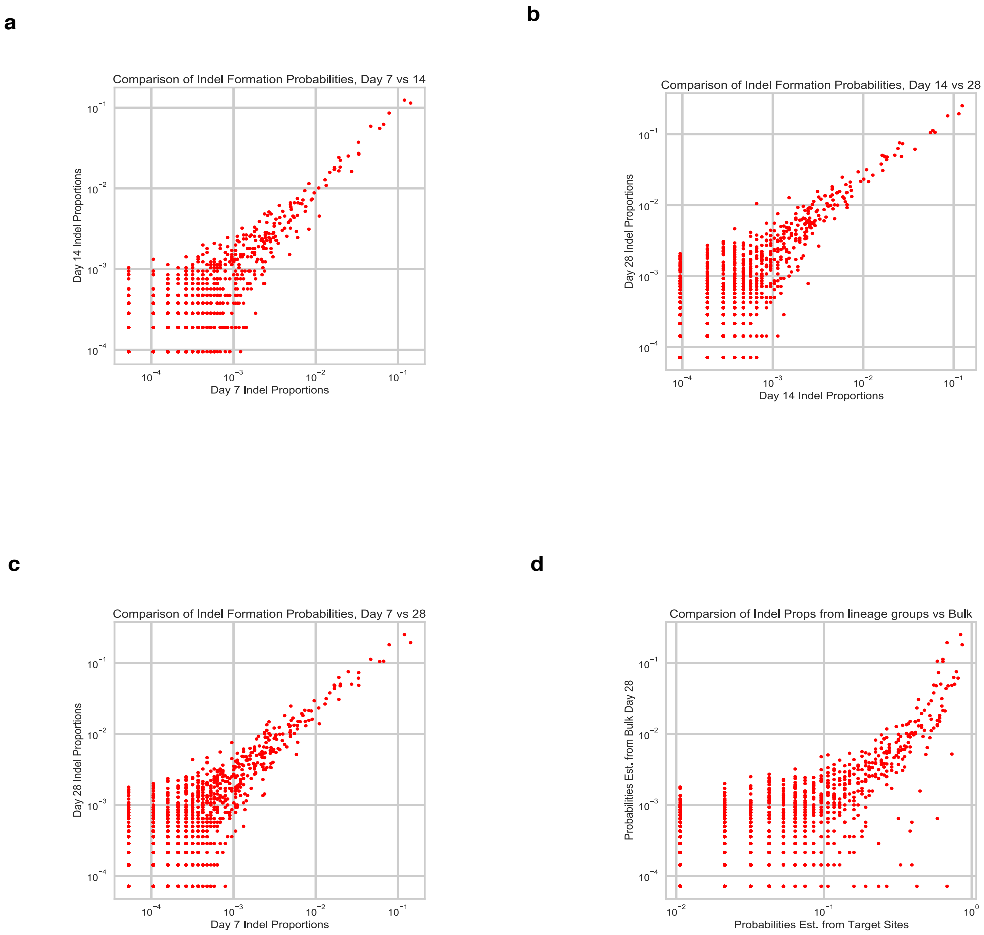

Fig. S20. Estimation of Prior Probabilities for Tree Reconstruction. Prior probabilities to be used during tree reconstruction can be determined from both a bulk assay and independent clonal populations. Prior probabilities of mutations were determined by calculating the proportion of unique intBCs that report a particular indel (see methods). The bulk assay consisted of several independent clones with non-overlapping intBCs grown over the course of 28 days. (a-c) report the correlation of indel formation probabilities between various time points in the bulk experiment. A strong correlation is observed between all time points: 7 and 14 (a), 14 and 28 (b) and 7 and 28 (c). Indel formation probabilities can also be calculated using the intBCs from each clone as independent measurements. Using this method, (d) reports the correlation between this lineage-group specific probability calculation and the last time point of the bulk assay.

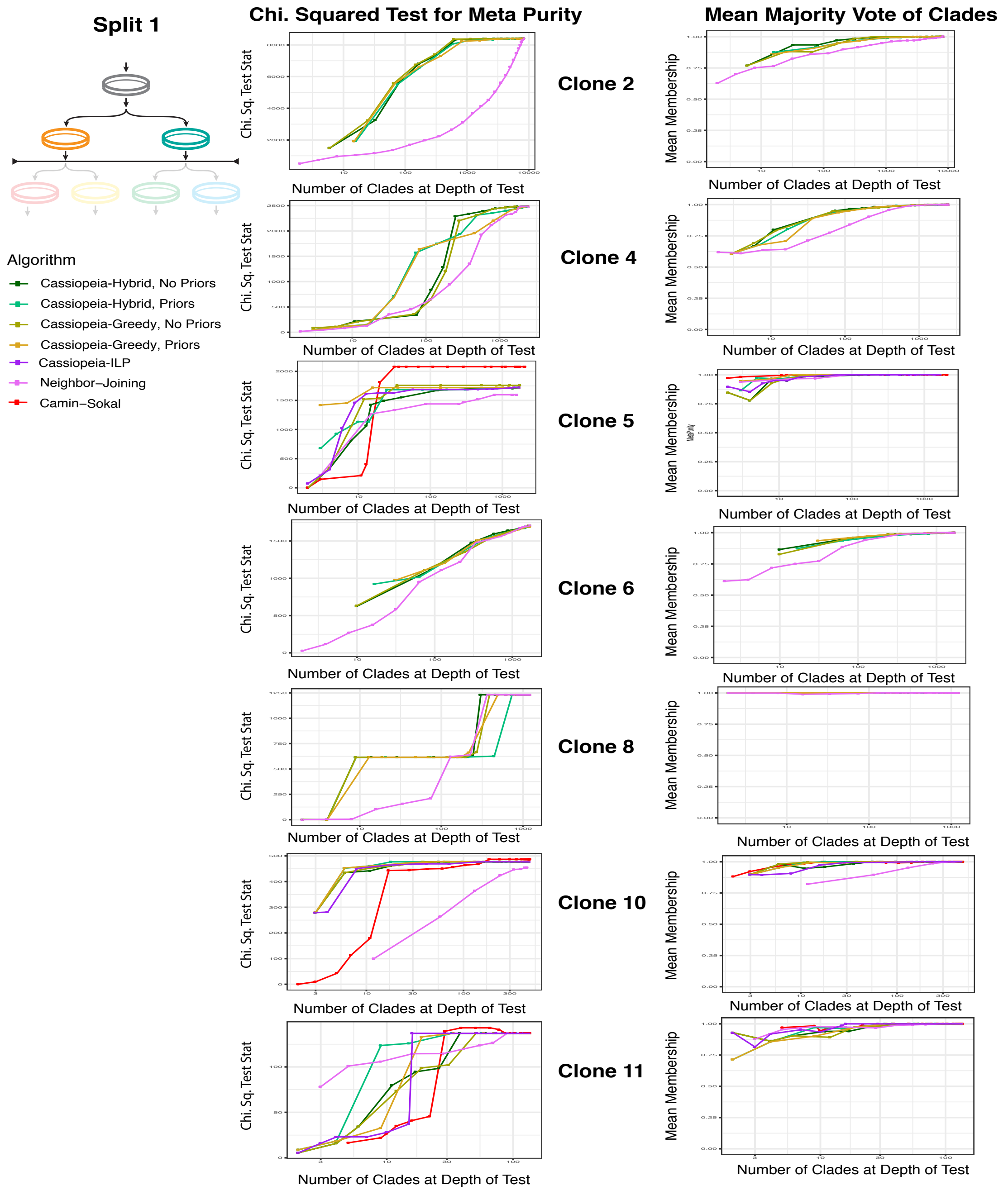

Fig. S21. Evaluation of algorithms on *in vitro* lineage tracing clones, First Split. Trees were reconstructed for the remaining clones in the *in vitro* dataset that consisted of more than 500 unique cell states. LG2, LG4, LG6, and LG8 passed this threshold and were reconstructed with Cassiopeia (with and without priors), greedy-only (with and without priors) and Neighbor-Joining. The statistics provided were taken with respect to the first split ID (see methods). For both Cassiopeia with and without priors, we used a cutoff of 200 cells and each instance of the ILP was allowed 5000s to converge on a maximum neighborhood size of 6000. For example, for Clone 5 it is difficult to pinpoint a single reason for the observed variability other than the fact it has a very small proportion of unique cells, namely that every leaf represents multiple cells (which can come from different plates), thus potentially making the performance criteria less robust.

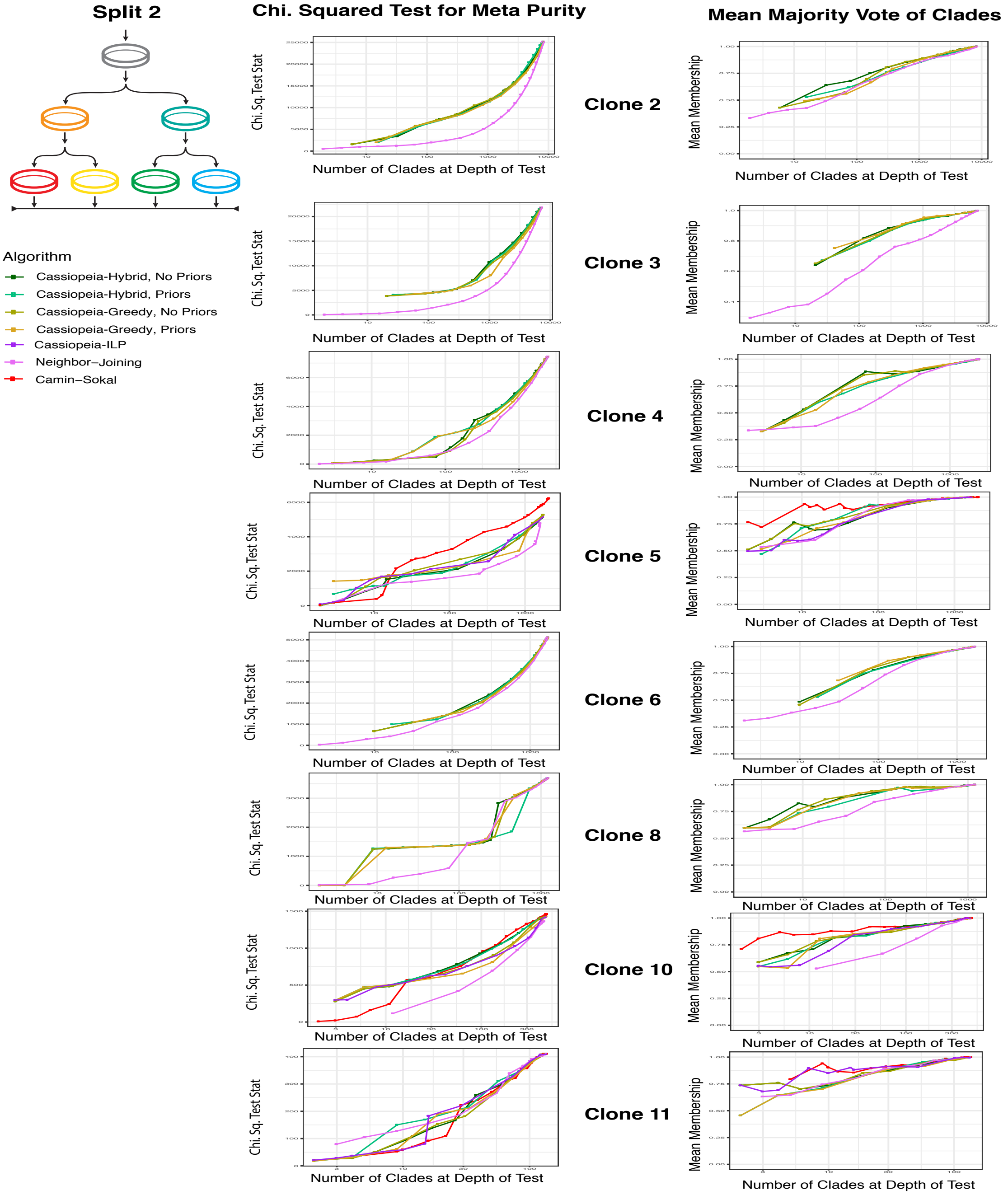

Fig. S22. Evaluation of algorithms on *in vitro* lineage tracing clones, Second Split. Trees were reconstructed for the remaining clones in the *in vitro* dataset that consisted of more than 500 unique cell states. LG2, LG4, LG6, and LG8 passed this threshold and were reconstructed with Cassiopeia (with and without priors), greedy-only (with and without priors) and Neighbor-Joining. The statistics provided were taken with respect to the second split ID (see methods). For both Cassiopeia with and without priors, we used a cutoff of 200 cells and each instance of the ILP was allowed 5000s to converge on a maximum neighborhood size of 6000.

a

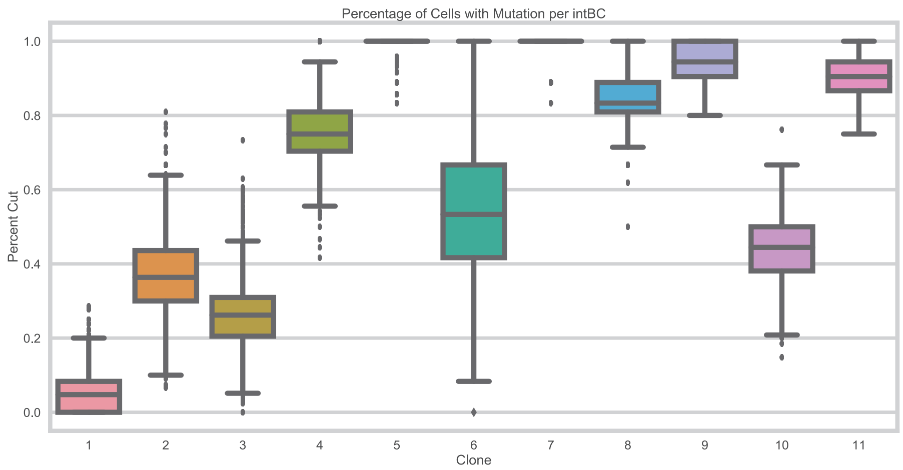

b

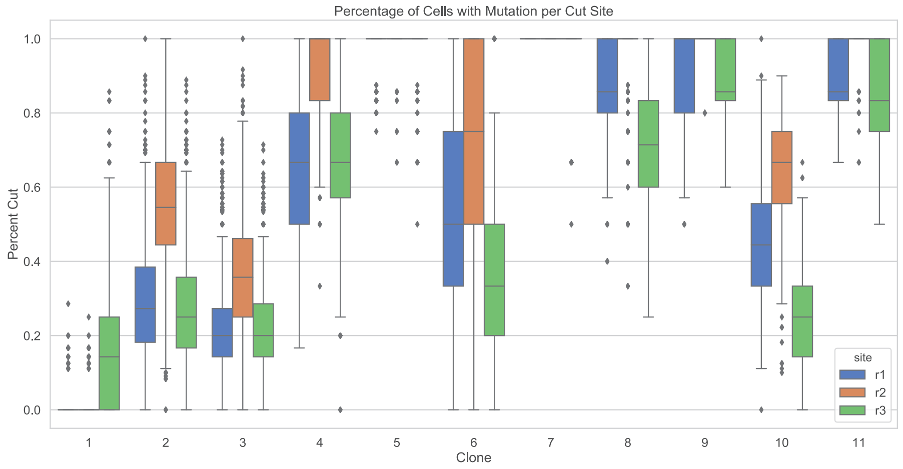

Fig. S23. Exhaustion of Target Sites across Clones. Target site exhaustion for each clone, as measured by the proportion of sites observed as edited after the experiment. (a) presents the percentage of mutated cells across all cut sites per clone. (b) details the distribution of mutated cells per cut site in each clone.

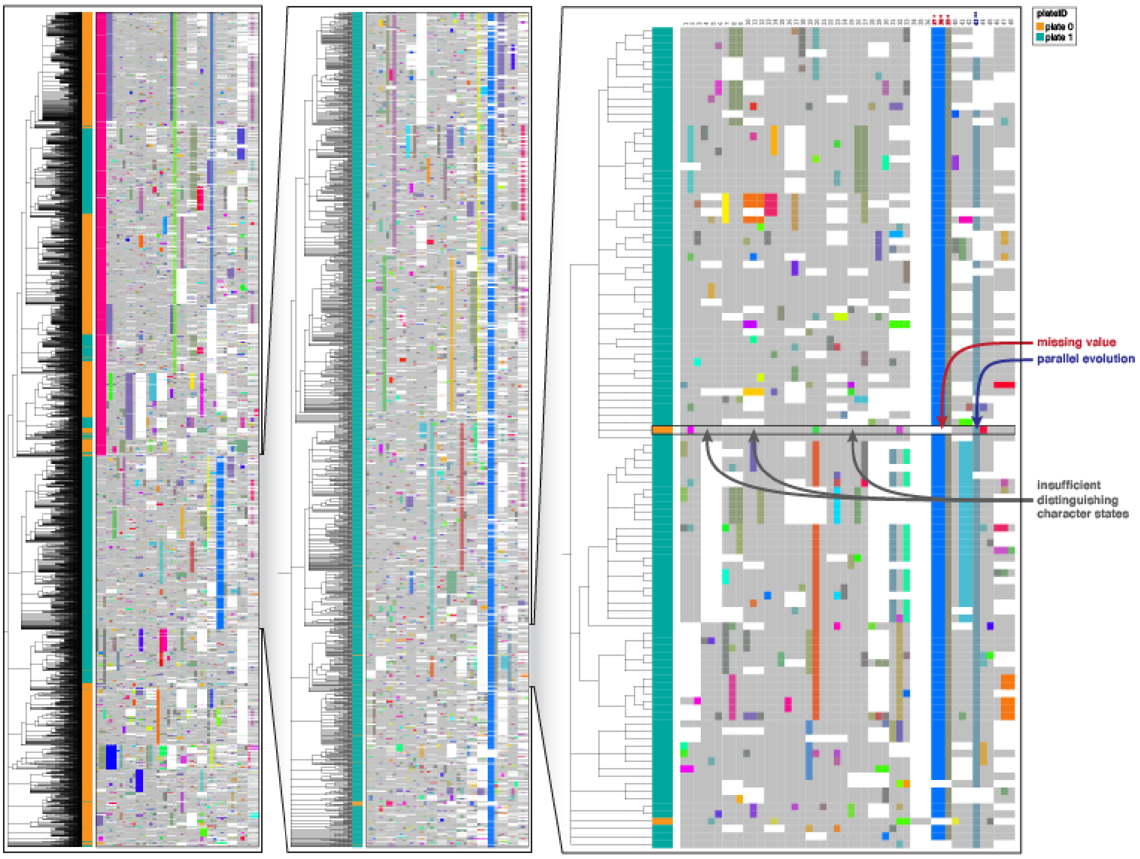

Fig. S24. Vignette of Inferential Mistakes for Clone 3. An example from the reconstruction of Clone 3 with Cassiopeia-Hybrid where a cell has been misplaced in the tree due to several factors. In this case, it is clear that the cell was placed where it is due to an instance of parallel evolution of the state in character 43 (as annotated in the figure). Because the cell contained this state, it was grouped with cells of a different plate also containing this mutation. Furthermore, the cell contains few distinguishing mutations thus making it difficult to infer the true value of the missing values located in characters 37-39.

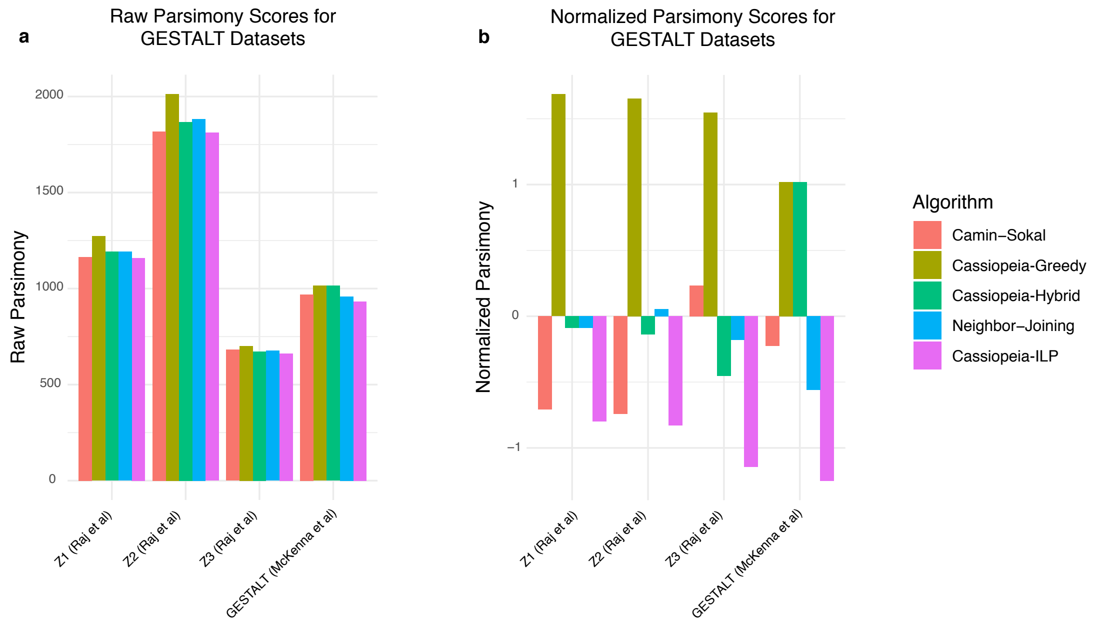

Fig. S25. Parsimony scores from reconstructions of the GESTALT datasets. (a) Raw and (b) normalized parsimony scores for the parsimony scores from the GESTALT datasets. Camin-Sokal, Neighbor-Joining, Cassipeia-Greedy, -Hybrid, and -ILP were run on datasets from Raj et al [6] and McKenna et al [3]. Raw parsimony scores are calculated as the number mutations present in a phylogeny (summing over the mutations along every edge of the tree). The normalized scores correspond to z-scores for each dataset.

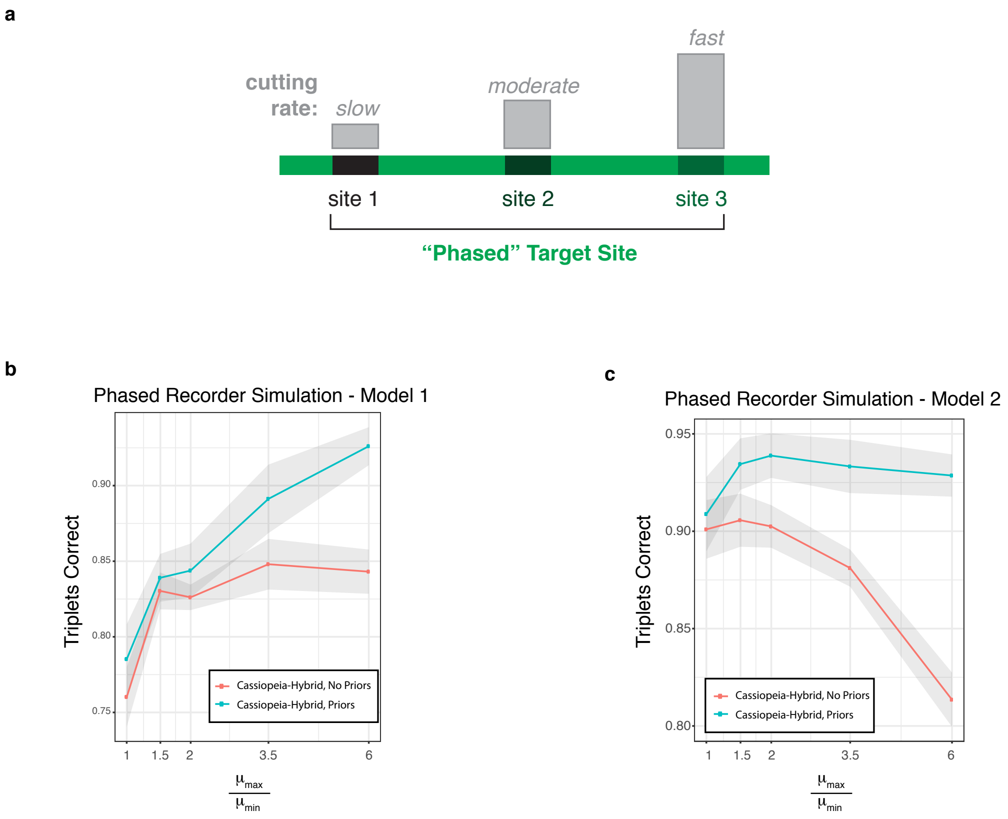

Fig. S26. “Phased Recorder” leverages variability across target sites. (a) Design concept of the “Phased Recorder.” (a) We simulated a “phased” editor, where each character is mutated at variable rates. (b-c) We varied the amount each character could very across 5 different experiments and simulated using two different indel formation rate models. Each cell had 50 characters with 10 states per character and a mean dropout of 10%. The amount of mutation variability is described with the ratio between the maximum and minimum mutation rates ($\frac{\mu_{max}}{\mu_{min}}$). Standard error is represented by shaded area. (b) Model 1 consists of drawing indels from a negative binomial distribution $NB(5, 0.5)$ where there are few “rare” indels. (c) Model 2 consists of drawing indels from the splined distribution of the empirical dataset’s indel formation rates, as used in other synthetic benchmarks.
