# Supplementary material for: Inference of single-cell phylogenies from lineage tracing data using Cassiopeia
Source: Genome Biol. 2020 Apr 14;21:92. doi: 10.1186/s13059-020-02000-8 (PMC7155257; doi:10.1186/s13059-020-02000-8)
Supplement: Supplementary file 2 — Additional file 2 Review history. [file 13059_2020_2000_MOESM2_ESM.docx]

Review History

**First round of review**

**Reviewer 1**

**Were you able to assess all statistics in the manuscript, including the appropriateness of statistical tests used?**

No

**Were you able to directly test the methods?**

Yes

**Comments to author:**

Jones, Khodaverdian, and Quinn et al. detail their Cassiopeia approach to lineage tree building, as well as detail a new experimental resource to test lineage reconstruction. Both the method and the new data set will be of great interest and use to the emerging single-cell lineage tracing field. The introduction does a nice job laying out many of the challenges that come from lineage reconstruction using CRISPR, and options for addressing these issues. The paper is extensive and explains various choices (and details them in the methods) well. I have a point below about widening the discussion section to include more recent developments in the field but overall, I am extremely enthusiastic about the impact of this paper.

- Aaron McKenna

Major points:

1. The future directions should include a note about parsimony serving as the basis for further tree refinement. Two examples are branch-length estimation and integration with single-cell transcriptional data. We're interested in branch-length estimation on cell lineage trees, as cell division times certainly vary depending on the cell type, tissue, stage of development, etc. You should consider citing Jean Feng's paper which aims to address this issue (https://www.biorxiv.org/content/10.1101/595215v1, self-serving of me). Additional it worth mentioning that there are interesting opportunities to incorporate single-cell state into lineage reconstruction, as the Bar-Joseph group recently showed (https://www.biorxiv.org/content/10.1101/630814v1).

2. The take-away from figure 4 is really vague. You say "Interestingly, we find that certain indels indeed span the different plates, thus indicating that Cassiopeia-Greedy chooses early indels to serve as splits. Moreover, the character matrix and the nested dissection of the tree demonstrate the abundant lineage information encoded in this clone - 96% of the 7,289 observed cells are marked uniquely." This all feels a bit squishy (now I'm being vague). The first point could be an early event, or although unlikely, homoplasy. The second point is nice, but maybe could say something about the relationship between the number of captured cell divisions (depth of the tree) and the suspected cell division count for this clone? Or something about that number in general with 96% of cells having lineage information? The figure is pretty (also figure 1 is beautiful btw), but it should be more informative.

Minor points:

1. I would probably drop the 'theoretically grounded' line from the abstract. To me it implies that other approaches aren't grounded, and I've heard from others that they had the same reaction. Certainly, there's been some more ad-hoc methods being used in this space, but the contrast in the paper is against methods with pretty well-established theoretical grounding (you even mention this when choosing maximum parsimony, ~ line 50, page 3).

2. Supplementary Figure 1 In the text you say "First, traditional algorithms were developed for the cases of few samples (in this case cells) and thus scalability is a major limitation (Sup- plementary Figure 1)." The figure is about the maximum neighbor size parameter, which doesn't really speak to the text?

3. Some scientific verbiage could be cut down. For instance, page 9, lines ~20, you could just say you estimated indel frequencies and their priors based on the data. The second half of this sentence doesn't make sense.

4. Figure 5A could be more informative. It doesn't really add anything as is, and it's actually a bit confusing as you label different points split one in figure 3A and 5A (they're the same plate, just before and after seeding). Maybe also a good place to explain the rest of the figure is clade 3

5. Could you add scores (or z-scores) to figure 6a. It would help understand the magnitude of differences here, as the color intensity is a bit hard to sort out.

**Reviewer 2**

**Were you able to assess all statistics in the manuscript, including the appropriateness of statistical tests used?**

Yes: The supplementary data and methods were extensive

**Were you able to directly test the methods?**

Yes

**Comments to author:**

Here, Jones et al., present Cassiopeia, an elegant set of maximum parsimony approaches for lineage tree reconstruction. Recently, several approaches to reconstruct lineage using accrued CRISPR/Cas9-based gene edits have emerged, with more tools on the horizon. A current gap in these advances has been represented by the paucity of computational tools to infer phylogenetic relationships accurately. Cassiopeia seeks to fill this gap, and here, the authors also provide simulation tools and experimental lineage tracing dataset, which incorporates ground truth to benchmark these phylogenetic inference approaches. Cassiopeia itself is elegant, and the authors make considerable effort to demonstrate its broad application. However, in this current presentation, these demonstrations (while helpful) detract from the core of the manuscript; in order to fully realize this tool and data set as a reference for future use, the data described in the paper should be analyzed/characterized more thoroughly. We have the following specific comments:

Major comments:

- A major concern with current CRISPR based lineage tracing techniques is parallel evolution. Could the authors explicitly incorporate this into their simulations? We realize the authors have included a parameter for the mutation rate of Cas9, but could the authors include or designate a specific number/percentage of alleles that evolved in parallel? This would then allow the authors to investigate the robustness of their methods concerning parallel evolution.

- Related to the above point about parallel evolution, while the experimental dataset the authors present is an elegant approach, it is not sufficiently characterized. For example, the authors state that "the large majority of cells are uniquely marked (71%)" How frequently do two independent cells acquire the same edits, making them impossible to distinguish from each other. Is this 71% before or after qc, error correction, and filtering? What is the labeling and detection rate, and are cell 'barcodes' silenced over time? This is important information that will help fuel future benchmarking efforts and aid experimental design.

- The authors developed their own elegant, simple methods for doublet (two types) and indel estimation; it would be helpful if they provided an explanation for why these algorithms for data processing are more appropriate or accurate than existing methods for detection of both of these phenomena. Is the prior probability or classifier more accurate than other strategies?

- The authors evaluate the accuracy of lineage reconstruction using "Meta Purity" and "Mean Majority Vote". It would be helpful to see a more thorough clone-to-clone comparison of their lineage reconstruction accuracy. For example, there appears to be a lot of variability in the "Meta Purity" estimates of Clones 5 and 11 from method to method. Is there an identifiable reason for this or specific characteristics of the clones which lead to this? This information could also be extremely for future benchmarking approaches using this dataset as there is clear clone-to-clone diversity that the authors could better leverage here.

- Related to the above point, more thorough comparison of the quality/accuracy of the lineage reconstructions from the original data could highlight the strengths/weaknesses of certain approaches depending on the characteristics of the clone/lineage.

- A better explanation of the range of parameters used during the simulations should be included. In Figure 3 the authors illustrate the ranges of each parameter tested during simulations. This is appreciated but based on the authors own data it seems that maybe the ranges of these parameters should be expanded as there are datapoints which fall outside of these ranges.

- It would be helpful if the authors expanded on when and what situations each of the methods should be used in terms of high mutation rate, large number of cells, etc. Including general guidelines on when to use one approach or the other would assist readers and end-users.

- In applying Cassiopeia to GESTALT data, the authors use parsimony to assess the performance of Cassiopeia. Indeed, these assessments are challenging in the absence of ground truth data. However, the authors should provide some description of these results in terms of whether it agrees with the known biology reported using this established lineage tracing approach.

- The base editor simulation was run for 400 cells - what is the scalability of this?

Minor comments:

- Panel D of figure S16 is not described in the caption for figure S16.

- On page 8, the authors state, "Furthermore, these simulations grant insight into how Cassiopeia's performance is modulated by various experimental parameters, suggesting design principles that can be optimized to bolster reconstruction accuracy." It would be helpful for the reader if the authors briefly expand on some of these design principles at this point in the text.

- The authors might consider including details of the cell line used for their in vitro experiment in the main text, not only in the methods section.

**Reviewer 3:**

**Were you able to assess all statistics in the manuscript, including the appropriateness of statistical tests used?**

Yes: There is not enough robusntess analysis here - see detailed comments

**Were you able to directly test the methods?**

No

**Comments to author:**

The paper by Jones et al follow up on an important and nice paper from Yosef, Meissner and Weismann groups introducing and using an improved lineage recorder for understanding differentiation and fate decisions in mouse embryos. In the present manuscript the authors' main theme is simulation and calibration of the phylogenetic reconstruction algorithm used for assembling recorder scarring traces into lineage trees. The authors introduce two algorithmic strategies (and a combined version of the two) and use simulation to support their use over naïve bottom-up neighbor joining and Camin-Sokal reconstructions. They also perform a calibration experiment involving two population splits to monitor performance on a known (albeit very simple) gold standard.

Overall all the authors work should be commended and it involves the development of a complex pipeline that identifies clones, cleans UMIs and doublets, filters bad characters and faulty clones and eventually applying a phylogenetic reconstruction approach to provide interpretable results as described in the original Nature paper. I'm however not sure the current paper is written in a way that maximize the utility and impact of the authors' work however. Some hopefully constructive comments for consideration are below. In addition, I think it is critical to make this manuscript more focused (e.g. on the pipeline) and less repetitive (e.g. can introduce the benchmark dataset, synthetic and experimental, and describe comparisons on them together rather than repeatedly, and more).

Major comments:

1) algorithmic discussion and analysis. Let's face it - molecular recording of scars in exponentially growing population is designed to make the life of reconstruction algorithms super simple. Indeed, Cassiopeia-greedy is working very well by applying an extremely simple top-down heuristics that only perform repeated splits on the next most informative allele. This is great - shouldn't be hidden behind algorithmic masks. I don't believe I was able to find in the paper's results and simulation support for using the more sophisticated Steiner strategy - it is generally only as good as the greedy approach in all, or almost all analyses. What are the problems that the more extensive optimizer is trying to solve? Is it sorting out more carefully, noisier, lower cardinality subtrees? If yes it should be shown explicitly and explain which tradeoffs, and what non-monotonicity prevent the greedy heuristic from performing well in the lower subtrees. If not, maybe the whole ILP strategy can be avoided? The authors can do more comparisons to std phylogenetic strategies instead.

2) using triplets statistics and comparisons of algorithmic success. It can be taken for granted (and/or demonstrated directly) that the first splits are well reconstructed. These splits dominate the %triplet correct statistics and reduce its resolution severely. We are left with very little insight into the real success of the reconstruction task. The authors can instead show data on the inferred and simulated tree-distance between nodes,

3) The authors should zoom on cells that are inferred to be located on a tree position that is very far from the ground truth and try to infer why? (errors? Missing data). The should provide more extensive statistics on the linkage between distance reconstruction accuracy and properties of the alleles, and more. This is where the simulation is supposed to be really helpful. I was quite disappointed with the conclusions that only bash the noise-prone NJ strategy but does not do much more. Even NJ can be improved with some feature selection.

4) Providing insight into the low-level filtering stages. It looks like more than algorithmic choices on phylogenetic reconstruction, the heuristics going into the decisions on clones, alleles, doublets play a major role on the overall quality of the approach. In fact, I recommend putting more emphasis on this, e.g., describing explicitly these stages in figure 1, with a bit more information and recommendations on parameters, and making the whole pipeline more transparent.

5) Finally, and really importantly, I don't understand how a paper doing Max parsimony phylogenetic reconstruction in 2020, in any domain, can avoid the very routine analysis of robustness using the bootstrap. This is absolutely essential (and it is not hard!) and should have been a corner stone of the authors analysis here.

**Authors Response**

**Point-by-point responses to the reviewers’ comments:**

REVIEWER 1

Jones, Khodaverdian, and Quinn et al. detail their Cassiopeia approach to lineage tree building, as well as detail a new experimental resource to test lineage reconstruction. Both the method and the new data set will be of great interest and use to the emerging single-cell lineage tracing field. The introduction does a nice job laying out many of the challenges that come from lineage reconstruction using CRISPR, and options for addressing these issues. The paper is extensive and explains various choices (and details them in the methods) well. I have a point below about widening the discussion section to include more recent developments in the field but overall, I am extremely enthusiastic about the impact of this paper.

- Aaron McKenna

Major points:

1. The future directions should include a note about parsimony serving as the basis for further tree refinement. Two examples are branch-length estimation and integration with single-cell transcriptional data. We're interested in branch-length estimation on cell lineage trees, as cell division times certainly vary depending on the cell type, tissue, stage of development, etc. You should consider citing Jean Feng's paper which aims to address this issue (https://urldefense.proofpoint.com/v2/url?u=https-3A__www.biorxiv.org_content_10.1101_595215v1&d=DwIGaQ&c=iORugZls2LlYyCAZRB3XLg&r=mWUJeXz3Vbp-ONTVLz5VU-Te05gUh9-NIW8X2fRxvFQ&m=ItL3WvxcASFnGcKLMP_j8FuAVDKQa-greK3I3jF5G0M&s=WocKp_wGnKP4OFwMMd_LStPZkiG9c9Q8J_NCZVdoZ4A&e= , self-serving of me). Additional it worth mentioning that there are interesting opportunities to incorporate single-cell state into lineage reconstruction, as the Bar-Joseph group recently showed (https://urldefense.proofpoint.com/v2/url?u=https-3A__www.biorxiv.org_content_10.1101_630814v1&d=DwIGaQ&c=iORugZls2LlYyCAZRB3XLg&r=mWUJeXz3Vbp-ONTVLz5VU-Te05gUh9-NIW8X2fRxvFQ&m=ItL3WvxcASFnGcKLMP_j8FuAVDKQa-greK3I3jF5G0M&s=2NxmG3ngo-j-QEtEFJRn1zQ3fw5D7LTMXjG6yyuYElg&e= ).

*Thank you for pointing us towards these excellent new developments in this field, we have now added these references to our discussion on p. 14.*

2. The take-away from figure 4 is really vague. You say "Interestingly, we find that certain indels indeed span the different plates, thus indicating that Cassiopeia-Greedy chooses early indels to serve as splits. Moreover, the character matrix and the nested dissection of the tree demonstrate the abundant lineage information encoded in this clone - 96% of the 7,289 observed cells are marked uniquely." This all feels a bit squishy (now I'm being vague). The first point could be an early event, or although unlikely, homoplasy. The second point is nice, but maybe could say something about the relationship between the number of captured cell divisions (depth of the tree) and the suspected cell division count for this clone? Or something about that number in general with 96% of cells having lineage information? The figure is pretty (also figure 1 is beautiful btw), but it should be more informative.

*We agree that the interpretation of this figure should be more precise, and have made additions to the text and analysis to supplement this figure. Firstly, we have amended the text to better clarify our point and to reflect alternative reasons for why indels may be shared across the plates (i.e., homoplasy, as you suggested - see p.10). Secondly, we now provide additional statistics from our tree reconstruction in Figure 4 - specifically, the depth of the tree for all the leaves (Figure 4d) and the agreement between mutation edit distance and the phylogenetic distance of all leaves (Figure 4e). Our discussion around this can be found on p.10-11.*

Minor points:

1. I would probably drop the 'theoretically grounded' line from the abstract. To me it implies that other approaches aren't grounded, and I've heard from others that they had the same reaction. Certainly, there's been some more ad-hoc methods being used in this space, but the contrast in the paper is against methods with pretty well-established theoretical grounding (you even mention this when choosing maximum parsimony, ~ line 50, page 3).

*We agree, and have removed this point from our abstract. To clarify, our intention was not to criticize the theoretical grounding of traditional approaches, but rather to highlight that Cassiopeia is also theoretically grounded.*

2. Supplementary Figure 1 In the text you say "First, traditional algorithms were developed for the cases of few samples (in this case cells) and thus scalability is a major limitation (Supplementary Figure 1)." The figure is about the maximum neighbor size parameter, which doesn't really speak to the text?

*Thank you for catching this. The figure reference has been corrected.*

3. Some scientific verbiage could be cut down. For instance, page 9, lines ~20, you could just say you estimated indel frequencies and their priors based on the data. The second half of this sentence doesn't make sense.

*Thank you for this suggestion. We have addressed this concern in re-writing portions of the text, including the above example (p.10).*

4. Figure 5A could be more informative. It doesn't really add anything as is, and it's actually a bit confusing as you label different points split one in figure 3A and 5A (they're the same plate, just before and after seeding). Maybe also a good place to explain the rest of the figure is clade

*In the interest of clarity, we have now removed this figure panel and have appended clarifying details to the legend of Figure 3a.*

5. Could you add scores (or z-scores) to figure 6a. It would help understand the magnitude of differences here, as the color intensity is a bit hard to sort out.

*We agree, and have added Z-scores to Fig. 6a.*

REVIEWER 2

Here, Jones et al., present Cassiopeia, an elegant set of maximum parsimony approaches for lineage tree reconstruction. Recently, several approaches to reconstruct lineage using accrued CRISPR/Cas9-based gene edits have emerged, with more tools on the horizon. A current gap in these advances has been represented by the paucity of computational tools to infer phylogenetic relationships accurately. Cassiopeia seeks to fill this gap, and here, the authors also provide simulation tools and experimental lineage tracing dataset, which incorporates ground truth to benchmark these phylogenetic inference approaches. Cassiopeia itself is elegant, and the authors make considerable effort to demonstrate its broad application. However, in this current presentation, these demonstrations (while helpful) detract from the core of the manuscript; in order to fully realize this tool and data set as a reference for future use, the data described in the paper should be analyzed/characterized more thoroughly. We have the following specific comments:

Major comments:

- A major concern with current CRISPR based lineage tracing techniques is parallel evolution. Could the authors explicitly incorporate this into their simulations? We realize the authors have included a parameter for the mutation rate of Cas9, but could the authors include or designate a specific number/percentage of alleles that evolved in parallel? This would then allow the authors to investigate the robustness of their methods concerning parallel evolution.

*We fully agree that parallel evolution in CRISPR-based lineage tracing experiments poses an important challenge for subsequent tree reconstruction. As such, we explicitly simulated parallel evolution events (Supp. Figure 4) and discuss their effects on tree reconstruction (p. 7-8). Specifically, we provided a series of in silico experiments exploring this in which we directly modulate the number of parallel evolution events and the depth at which they occur (Supp. Figure 4) and then evaluate the precision of greedy splits in these cases. We reason that parallel evolution most heavily affects the greedy heuristic, and thus focus this particular analysis on Cassiopeia-Greedy.*

*Also, as noted by the reviewer, we implicitly evaluate the effects of parallel evolution on all algorithms in Figure 2, in particular while simulating low-information capacity regimes with high Cas9 mutation rates or with few states per character. We discuss the effects of these settings on the performance of the greedy algorithm (compared to Cassiopeia-ILP or -Hybrid) on p. 7. As suggested by the reviewer, we now explicitly highlight how parallel evolution in particular dominates in these regimes through an evaluation of the proportion of parallel evolution events in each simulation regime (Supp. Figure 12).*

- Related to the above point about parallel evolution, while the experimental dataset the authors present is an elegant approach, it is not sufficiently characterized. For example, the authors state that "the large majority of cells are uniquely marked (71%)" How frequently do two independent cells acquire the same edits, making them impossible to distinguish from each other. Is this 71% before or after qc, error correction, and filtering? What is the labeling and detection rate, and are cell 'barcodes' silenced over time? This is important information that will help fuel future benchmarking efforts and aid experimental design.

*Regarding the unique labeling of cells via the state of cut sites. The 71% referenced in the main text of our manuscript refers to the number of unique labelings after all QC (which encompasses error correction of UMIs, the filtering of cells on the basis of UMIs & reads, and mapping of mutations to integration barcodes detailed in our Methods p. 19-22). Importantly, in the cases where we see two cells with identical character states it is unlikely due to parallel evolution but rather due to cutting rates that are too fast (i.e. a single character state was fixed early in the experiment) or too slow (i.e. no new mutations occurred to distinguish between sister cells). This is because the number of total possible character states in this experiment is so vast (in particular, assuming 40 characters and at least 20 states per character, we have 20^40 possible mutation states) that it is extremely unlikely that duplicate character states arose independently. We have now clarified this point in the main text (p.10). We have also added figures that characterize the percentage of unique states in each clone (Figure 3f) and the proportion of cut sites that have indels (Supplementary Figure 23). Indeed, here we find that the clones that had significantly lower proportion of unique states (i.e. where most cells have the same mutations) are also associated with “exhausted” characters (i.e. a majority of target sites have been cut). This observation suggests that the cutting rate in these clones was very high, and thus the cuts occurred very early in the process and throughout all sites (this is especially problematic with Clone 7 where there are only 3 captured intBCs - see Supp. Figure 18c), leaving the subsequent progeny identical in terms of their target sites. We discuss how these features affect lineage information content in the main text (p.11).*

*Regarding the issue of quality control, we now grant more attention to the processing pipeline in our manuscript. In particular, we have added a new panel to Figure 1 highlighting the main modules of the Cassiopeia processing pipeline and a new section in the Methods on guidelines for successful processing of the sequencing data (see: “Guidelines for Final Quality Control”).*

*By “labeling rate” we assume that the reviewer means the rate in which mutations occur in each site. Our evaluation of these rates is described in our Method (p. 22), and the resulting estimated rates (which we used to guide our simulations) are presented in Figure 3d, and are also explored via new analyses of target site exhaustion in Supp. Figure 23.*

*By “detection rate” we assume the reviewer refers to the % of intBC that are observed in each cell (out of the set of inBCs in its respective clone). A distribution of “detection rates” is presented in Supp. new Figure 18d, as well as Figure 3e. It is important to note though that it is challenging to estimate which failures to detect an intBC (i.e. “dropout”) result from heritable (e.g., large genomic resections, “unsequenceable” Target Site amplicons, or epigenetic silencing) vs. non-heritable source (i.e., technical reasons like limited sensitivity of the 10x platform). We note in the Discussion that more targeted experiments are necessary to deduce how often intBCs are being silenced biologically (as opposed to technically) and what factors are predominantly causing this silencing).*

- The authors developed their own elegant, simple methods for doublet (two types) and indel estimation; it would be helpful if they provided an explanation for why these algorithms for data processing are more appropriate or accurate than existing methods for detection of both of these phenomena. Is the prior probability or classifier more accurate than other strategies?

*A unique doublet detection method was required for this dataset as we did not have transcriptomic information ([1], [2]) nor multiplexing barcodes ([3],[4]). Instead, we are using the intBCs and the cuts themselves as a surrogate for multiplexed barcodes, and use a similar approach (as in [3]; see supplementary methods p. 24-25) to detect doublets given noisy barcode information. Indeed, we demonstrate in Supp. Figure 19 that we could use the intBCs in each “cell” to identify “inter-doublets” (the respective cells in the doublet come from different clones), in a manner akin to identifying doublets that come from two or more donors (due to genetic diversity, as in [8]). Generally, it is difficult to identify doublets from within the same sample, unless there is accompanying transcriptomic data. Here we address this by identifying conflicting character states (i.e., two or more possible indel outcomes at the same location in a given cell-barcode) which allow us to find “intra-doublets” that are likely within a clone. As this is an important point, we have added a more in depth description of the doublet detection strategy to the main text (p. 10) and noted it in Figure 1b.*

*The problem of indel prior estimation is of fundamental importance in the field of CRISPR engineering. While there exist approaches for predicting Cas9-induced indels, these can be confounded by biological and technical effects (e.g. cell type, cell cycle stage, sequence context, etc.), precluding one from generalizing well to unseen datasets. In light of this, we took an empirical approach to estimating indel priors. As in our previous work [5], here we found this approach suitable as it agrees well with larger pooled experiments to characterize indel frequencies (Supp. Figure 20).*

- The authors evaluate the accuracy of lineage reconstruction using "Meta Purity" and "Mean Majority Vote". It would be helpful to see a more thorough clone-to-clone comparison of their lineage reconstruction accuracy. For example, there appears to be a lot of variability in the "Meta Purity" estimates of Clones 5 and 11 from method to method. Is there an identifiable reason for this or specific characteristics of the clones which lead to this? This information could also be extremely for future benchmarking approaches using this dataset as there is clear clone-to-clone diversity that the authors could better leverage here.

*We suspect that the reason for so much clone-to-clone variability stems from the variable parameters of each clone, in particular the observed number of cells, number of unique lineage states, and estimated Cas9 mutation rate (see Fig. 3, Supp. Figures 18 and 23). For example, for Clone 5 it is difficult to pinpoint a single reason for the observed variability other than the fact it has a very small proportion of unique cells, namely that every leaf represents multiple cells (which can come from different plates), thus potentially making the performance criteria less robust. We relate to this point on p. 11 and on the legend of Supp. Figure 21.*

- Related to the above point, more thorough comparison of the quality/accuracy of the lineage reconstructions from the original data could highlight the strengths/weaknesses of certain approaches depending on the characteristics of the clone/lineage.

*To address this, we have added several figures, which provide more information on each clone (i.e., # observed intBC [Supp. Figure 18d], % mutated sites [Supp. Figure 23], and % uniquely labeled cells [Figure 3f]; in addition to other properties [e.g., clone size] previously included in Fig 3). These figures can be considered along with the performance of the different algorithms (Supp. Figures 21 and 22), thus better mirroring the simulation analysis. We now also relate to this point of how empirical parameters related to different clones affect the reconstructions in the text (see p. 11).*

- A better explanation of the range of parameters used during the simulations should be included. In Figure 3 the authors illustrate the ranges of each parameter tested during simulations. This is appreciated but based on the authors own data it seems that maybe the ranges of these parameters should be expanded as there are datapoints which fall outside of these ranges.

*We have extended the simulation parameter ranges, particularly with the mutation rate to fully represent the observed parameter ranges (Figure 2). We opted to stop benchmarking the number of states as the trend becomes quite clear after 100 states per character.*

- It would be helpful if the authors expanded on when and what situations each of the methods should be used in terms of high mutation rate, large number of cells, etc. Including general guidelines on when to use one approach or the other would assist readers and end-users.

*We thank the reviewer for this suggestion and have expanded our discussion around the advantages of each algorithm from our simulation study (p. 7 - for example, suggesting how Neighbor-Joining and Camin-Sokal may be appropriate algorithms in regimes of low-dropout and few characters); From the simulations we also describe more in depth which parameters should be optimized for future lineage recorders in light of how the algorithms perform (see p.8-9). Furthermore, we have added a section to our Discussion describing our recommendations on when to use each of Cassoipeia’s modules on p.14.*

- In applying Cassiopeia to GESTALT data, the authors use parsimony to assess the performance of Cassiopeia. Indeed, these assessments are challenging in the absence of ground truth data. However, the authors should provide some description of these results in terms of whether it agrees with the known biology reported using this established lineage tracing approach.

*We agree with the reviewer that this would further support the generalizability of Cassiopeia. To this end, we have added a new analysis building on our application of Cassiopeia to GESTALT-type data: in Figure 6b, we now show the functional enrichment of tissue-type (as annotated in Raj et al [5]) of trees for each zebrafish built with ILP and Camin-Sokal, measured with our mean majority vote statistic. Here, we demonstrate that Cassiopeia finds more parsimonious solutions (Figure 6a) and that the tissue-type enrichment below nodes in the tree is conserved, as found in the original study. Our reference to this result can be found on p.12.*

- The base editor simulation was run for 400 cells - what is the scalability of this?

*We used the same simulation engine and algorithmic implementation to investigate the base editor’s potential as the one used in the scalability analysis on Supp. Figure 5 and did not observe that Cassiopeia-Hybrid was slower in the base-editor analysis. We have added this observation on p. 13.*

Minor comments:

- Panel D of figure S16 is not described in the caption for figure S16.

*We thank the reviewer for catching this mistake and have fixed it.*

- On page 8, the authors state, "Furthermore, these simulations grant insight into how Cassiopeia's performance is modulated by various experimental parameters, suggesting design principles that can be optimized to bolster reconstruction accuracy." It would be helpful for the reader if the authors briefly expand on some of these design principles at this point in the text.

*We thank the reviewer for this suggestion and have added a sentence to our conclusions detailing these design principles on p.8-9*

- The authors might consider including details of the cell line used for their in vitro experiment in the main text, not only in the methods section.

*We agree, and now include a brief description of the cell line in the introduction to the in vitro experiment (p.9).*

REVIEWER 3

The paper by Jones et al follow up on an important and nice paper from Yosef, Meissner and Weismann groups introducing and using an improved lineage recorder for understanding differentiation and fate decisions in mouse embryos. In the present manuscript the authors' main theme is simulation and calibration of the phylogenetic reconstruction algorithm used for assembling recorder scarring traces into lineage trees. The authors introduce two algorithmic strategies (and a combined version of the two) and use simulation to support their use over naïve bottom-up neighbor joining and Camin-Sokal reconstructions. They also perform a calibration experiment involving two population splits to monitor performance on a known (albeit very simple) gold standard.

Overall all the authors work should be commended and it involves the development of a complex pipeline that identifies clones, cleans UMIs and doublets, filters bad characters and faulty clones and eventually applying a phylogenetic reconstruction approach to provide interpretable results as described in the original Nature paper. I'm however not sure the current paper is written in a way that maximize the utility and impact of the authors' work however. Some hopefully constructive comments for consideration are below. In addition, I think it is critical to make this manuscript more focused (e.g. on the pipeline) and less repetitive (e.g. can introduce the benchmark dataset, synthetic and experimental, and describe comparisons on them together rather than repeatedly, and more).

Major comments:

1) algorithmic discussion and analysis. Let's face it - molecular recording of scars in exponentially growing population is designed to make the life of reconstruction algorithms super simple. Indeed, Cassiopeia-greedy is working very well by applying an extremely simple top-down heuristics that only perform repeated splits on the next most informative allele. This is great - shouldn't be hidden behind algorithmic masks. I don't believe I was able to find in the paper's results and simulation support for using the more sophisticated Steiner strategy - it is generally only as good as the greedy approach in all, or almost all analyses. What are the problems that the more extensive optimizer is trying to solve? Is it sorting out more carefully, noisier, lower cardinality subtrees? If yes it should be shown explicitly and explain which tradeoffs, and what non-monotonicity prevent the greedy heuristic from performing well in the lower subtrees. If not, maybe the whole ILP strategy can be avoided? The authors can do more comparisons to std phylogenetic strategies instead.

*We agree with the reviewer that it is encouraging that Cassiopeia-Greedy is often able to reconstruct accurate trees. However, the Cassiopeia-Greedy heuristic becomes less accurate when there is increased parallel evolution (see Supp. Figure 4). Specifically, the datasets that we observe from both in vitro and in vivo experiments are noisy, and complex in the sense that they violate assumptions of perfect phylogeny (see Supp. Figure 24 for example). Having no restriction on the extent of parallel evolution makes the problem of phylogenetic reconstruction NP-Hard, and thus there is no guarantee for the greedy approach to be sufficient. For these reasons, we believe that there is merit in putting forth a method that comes with better theoretical guarantees (i.e. Cassiopeia-ILP), and we evaluate its benefits and disadvantages compared with the greedy approach. Finally, it is worth mentioning the complexity of the problem in real-world instances motivated a recent DREAM challenge put forth by the Allen Institute (https://www.synapse.org/#!Synapse:syn20692755/wiki/595096).*

*Close inspection of our data indicates that Cassiopeia-ILP outperforms Cassiopeia-Greedy in several important cases. Specifically, when the number of states per character is small (~2-10) or the mutation rate is high (>10%), Cassiopeia-ILP (and to a less extent Cassiopeia-hybrid) clearly outperforms Cassiopeia-Greedy (Figure 2 & Supp. Figure 10), likely because these are regimes in which parallel evolution is most predominant (see p. 7-8). To clarify this point, we have added Supp. Fig. 12, in which we evaluated the extent of parallel evolution in each simulation scenario.*

*Furthermore, it is important to note that Cassiopeia-Greedy in theory cannot surpass Cassiopeia-ILP in terms of parsimony, in cases where the ILP procedure fully converges (which is the case for small instances). Indeed, it is clear from the GESTALT analysis that Cassiopeia-ILP finds by far the most parsimonious solution in smaller datasets (Figure 6 & Supp. Figure 25).*

*In our updated manuscript, we have extended the simulations to include higher mutation rates where Cassiopeia-ILP performs noticeably better (Figure 2b). We have also added observations on p.7-8 focusing in on regimes of small number of states per character where Cassiopeia-ILP outperforms Cassiopeia-Greedy. To more clearly motivate the use-cases of Cassopeia’s modules, we have also added a section to the Discussion suggesting in which cases to use Cassiopeia-Greedy, -Hybrid, and -ILP on p.14.*

2) using triplets statistics and comparisons of algorithmic success. It can be taken for granted (and/or demonstrated directly) that the first splits are well reconstructed. These splits dominate the %triplet correct statistics and reduce its resolution severely. We are left with very little insight into the real success of the reconstruction task. The authors can instead show data on the inferred and simulated tree-distance between nodes,

*We appreciate the concerns about using the triplets correct statistic in assessing phylogenetic accuracy. We have designed the calculation of the triplets correct score (used in the original submission) to avoid this concern. Specifically, we stratify the (randomly sampled) triplets of cells in accordance with the lowest common ancestor (LCA) of the three cells in the ground truth tree. We then report the average success over all LCA depths. This way, triplets whose LCA is near the root and whose accuracy likely rellies on the first inferred splits (which are indeed the majority of triplets) will not dominate the score. To better address this concern, we emphasize this aspect of the triplets in our caption for Supp. Figure 7 (in addition to the discussion in the text, p. 6).*

*To further address the reviewer’s concern as to whether the triplets correct statistic truly captures node-to-node distances between trees, we now present an additional panel in Supp. Figure 7, accompanying our introduction of the triplet correct statistic. Here, we demonstrate that the correlation between pairwise node distances between a reconstruction and the true tree is highly correlated to the triplet correct metric (with a Pearson’s correlation of 0.96). For the reasons listed on p.6, however, we opt to use the triplets correct statistic as opposed to phylogenetic distance (though there would be little practical difference).*

3) The authors should zoom on cells that are inferred to be located on a tree position that is very far from the ground truth and try to infer why? (errors? Missing data). The should provide more extensive statistics on the linkage between distance reconstruction accuracy and properties of the alleles, and more. This is where the simulation is supposed to be really helpful. I was quite disappointed with the conclusions that only bash the noise-prone NJ strategy but does not do much more. Even NJ can be improved with some feature selection.

*We thank the reviewer for this insight and agree it is important for users to understand the points of failure for phylogenetic reconstruction in Cassiopeia and beyond. In this paper we have opted to focus on the relationship between global properties of performance (triplets correct, plate purity, parsimony) and global properties of the tree (e.g., # leaves) or dataset (e.g., % missing entries). This is because it is not very clear how to systematically define “local” properties of performance (i.e., associate an error level with each leaf node). Instead, we opted to provide a detailed exposition of an individual case through a vignette from our real data demonstrating certain pathologies in the data that can cause mistakes in the reconstruction (see Supp. 24 & p.11). We also provide a new figures, evaluating the distance reconstruction accuracy more directly by comparing true vs. inferred tree distances (Supp. Figure 7b); as well as inferred tree distances vs. allelic distances (Fig, 4e).*

*Furthermore, we did not intend to criticize unfairly the Neighbor-Joining strategy but rather observe relative performances. We have now expanded the discussion to the failure points of Cassiopeia where we highlighted examples where we feel Neighbor-Joining and Camin-Sokal are suitable approaches, such as on p.6 and p.11. We have also noted that Neighbor-Joining can be improved with certain approaches on p.7.*

4) Providing insight into the low-level filtering stages. It looks like more than algorithmic choices on phylogenetic reconstruction, the heuristics going into the decisions on clones, alleles, doublets play a major role on the overall quality of the approach. In fact, I recommend putting more emphasis on this, e.g., describing explicitly these stages in figure 1, with a bit more information and recommendations on parameters, and making the whole pipeline more transparent.

*We thank the reviewer for this suggestion and agree that this facet of the approach was overlooked in the original manuscript and have decided to further emphasize the Cassiopeia processing pipeline in the updated text and figures. We have made efforts to make this more transparent: firstly, we have redesigned Figure 1 with a focus on the processing pipeline. In our figure caption, we are sure to include descriptions of when key steps occur: such as doublet detection, cell filtering, indel calling, and clonal assignment. Secondly, we have inserted in the main text a more specific reference to the methods on p. 9 where we discuss the heuristics we used for low-level filtering and pre-processing. Thirdly, we have added a section to the Methods detailing guidelines for processing the sequencing data (entitled “Guidelines for Final Quality Control”).*

5) Finally, and really importantly, I don't understand how a paper doing Max parsimony phylogenetic reconstruction in 2020, in any domain, can avoid the very routine analysis of robustness using the bootstrap. This is absolutely essential (and it is not hard!) and should have been a corner stone of the authors analysis here.

*We agree with the reviewer that this is an important analysis for phylogeny studies. We have found difficulty applying Felsenstein’s bootstrapping approach to our datasets in general mostly due to the strictness with which a clade is considered reproducible across samples, an insight consistent with recent work from the Gascuel group (Lemoine et al. Nature 2018 [6]). To address this, we have employed the approach described in Lemoine et al [6] which suggests using a transfer metric to define the reproducibility of clades across samples. Using this approach, we now provide a robustness analysis of our simulated trees using Cassiopeia-Greedy, -ILP, and Neighbor-Joining in Supp. Figure 14 which indicates that Cassiopeia’s modules are robust to this data type (see p. 7).*

*To enable future efforts assessing internal branch support for Cassiopeia’s reconstructions, we now provide new functions in our software codebase supporting bootstrapping & consensus tree finding:*

*In cassiopeia.TreeSolver.data_pipeline we provide the following:*

*create_bootstrap(..): a function that takes in a character matrix and creates several bootstrapped samples of this character matrix.*

*leave_one_out(..): a function that takes in an allele table and creates character matrices that are missing a single integration barcode.*

*In cassiopeia.TreeSolver.utilities we provide a function:*

*find_consensus_tree(..): a function that takes in a list of trees, inferred using from the bootstrap or leave_one_out character matrices, and returns a consensus tree (the support cutoff is also input by the user, allowing one to create a majority vote consensus tree or a greedy consensus tree).*

*REFERENCES:*

*1 McGinnis et al., “DoubletFinder: Doublet Detection in Single-Cell RNA Sequencing Data Using Artificial Nearest Neighbors”. Cell Systems (2019)*

*2 Wolock et al, “Scrublet: Computational Identification of Cell Doublets in Single-Cell Transcriptomic Data”. Cell Systems (2019)*

*3 Stoeckius et al., “Cell Hashing with barcoded antibodies enables multiplexing and doublet detection for single cell genomics”. Genome Biology (2018)*

*4 McGinnis, Patterson, et al., “MULTI-seq: sample multiplexing for single-cell RNA sequencing using lipid-tagged indices”. Nature Methods (2019)*

*5 Chan, Smith, et a., “Molecular recording of mammalian embryogenesis”. Nature (2019)*

*6 Lemoine, et al, “Renewing Felsenstein’s phylogenetic bootstrap in the era of big data”. Nature (2018)*

*7 Raj et al, “Simultaneous single-cell profiling of lineages and cell types in the vertebrate brain.” Nature Biotechnology (2018)*

*8 Kang et al, “Multiplexed droplet single-cell RNA-sequencing using natural genetic variation.” Nature Biotechnology (2018)*

**Second round of review**

**Reviewer 2**

The authors have addressed all my previous points and concerns.

**Reviewer 3**

The authors have addressed my concerns - I recommend publication.
